# A Highly Luminescent Nitrogen-Doped Nanographene as an Acid- and Metal-Sensitive Fluorophore for Optical Imaging

**DOI:** 10.1021/jacs.1c04880

**Published:** 2021-07-05

**Authors:** Enquan Jin, Qiqi Yang, Cheng-Wei Ju, Qiang Chen, Katharina Landfester, Mischa Bonn, Klaus Müllen, Xiaomin Liu, Akimitsu Narita

**Affiliations:** † Max Planck Institute for Polymer Research, Mainz 55128, Germany; ⊥ Institute of Physical Chemistry, Johannes Gutenberg-University, Duesbergweg 10-14, Mainz 55128, Germany; § Organic and Carbon Nanomaterials Unit, Okinawa Institute of Science and Technology Graduate University, Kunigami-gun, Okinawa 904-0495, Japan; ‡ College of Chemistry, Nankai University, Tianjin 300071, China

## Abstract

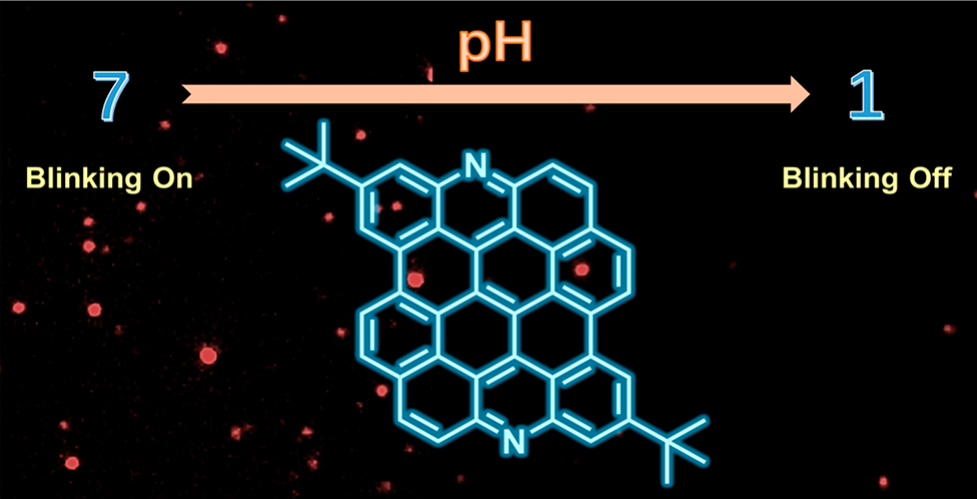

Dibenzo[*hi*,*st*]ovalene (DBOV) has excellent photophysical properties, including strong fluorescence and high ambient stability. Moreover, the optical blinking properties of DBOV have enabled optical super-resolution single-molecule localization microscopy with an imaging resolution beyond the diffraction limit. Various organic and inorganic fluorescent probes have been developed for super-resolution imaging, but those sensitive to pH and/or metal ions have remained elusive. Here, we report a diaza-derivative of DBOV (N-DBOV), synthesized in eight steps with a total yield of 15%. Nitrogen (N)-bearing zigzag edges were formed through oxidative cyclization of amino groups in the last step. UV–vis and fluorescence spectroscopy of N-DBOV revealed its promising optical properties comparable to those of the parent DBOV, while cyclic voltammetry and density functional theory calculations highlighted its lower orbital energy levels and potential *n*-type semiconductor character. Notably, in contrast to that of the parent DBOV, the strong luminescence of N-DBOV is dependent on pH and the presence of heavy metal ions, indicating the potential of N-DBOV in sensing applications. N-DBOV also exhibited pH-responsive blinking, which enables pH-sensitive super-resolution imaging. Therefore, N-DBOV appears to be a highly promising candidate for fluorescence sensing in biology and environmental analytics.

## Introduction

The ability to locally, and with high precision, determine pH and/or the presence of specific metal ions using fluorescent molecules is important for several disciplines, including biology^1−4^ and environmental monitoring.^5−7^ Detection of transition metal ions is crucial to the diagnosis and/or prevention of diseases caused by dysregulation of metal-ion homeostasis, such as anemia and Alzheimer’s disease.^4^ In biology, protons play a critical role in living cells to support various biological activities.^8^ The currently available pH- and/or metal ion-sensitive fluorophores consist of two classes: organic dyes^9^ and nanomaterial-based probes,^10^ e.g., carbon dots^11,12^ and semiconductor quantum dots,^13,14^ which have been intensively investigated in the past decade. Organic dyes, such as rhodamines and cyanine derivatives, can detect systems with high specificity and brightness.^2,9^ On the other hand, carbon dots and quantum dots can serve as low-cost probes with high chemical stability.^15^ However, the limited photostability, resulting in fast photobleaching, remains a common bottleneck for most existing probes.^16^ Different approaches, such as excitation light dose engineering^17^ and use of anti-fading agents,^16^ have been proposed to suppress the photobleaching, but such methods still typically increase the complexity of detection and narrow the range of applications.

On the other hand, pH-sensitive fluorescence probes, especially for optical super-resolution nanoscale imaging/detection beyond the diffraction limit of conventional light microscopy, remain rare.^2^ Optical super-resolution microscopy includes two groups of methods: stimulated emission depletion (STED) microscopy^18^ and single-molecule localization microscopy (SMLM).^19^

For STED microscopy, the fluorophores need to exhibit a significant amount of emission at the depletion wavelength of the used fluorophore, as this determines the efficiency of stimulated emission. Meanwhile, high brightness and photostability under the given imaging conditions are also prerequisites. Currently, only super-resolution pH indicator molecules based on the two-fluorophore design have been used as pH probes in STED microscopy, with a limited dynamic pH detection range of 5–7.^2^ For SMLM, photoblinking fluorophores are needed,^20^ and no pH-sensitive probe suitable for SMLM imaging has been reported, to the best of our knowledge.

Similar challenges exist for metal-ion sensing: most of the existing fluorophores are less suited for probing applications, including bioimaging, due to chemical or photophysical limitations.^21−23^ In particular, to avoid possible photoinduced biosample damage, excitation light in the longer visible and near-infrared wavelength is preferred.^24^ However, many reported metal-sensing dyes need higher photon energy for excitation, such as blue or even UV light, which is out of the biowindow.^24−27^

Clearly, new fluorescence probes with enhanced photostability and a wider dynamic range of pH detection and metal-ion probing, especially suitable for super-resolution microscopy, are in high demand.

Nanographenes, nanoscale polycyclic aromatic hydrocarbons (PAHs), have enormous promise because of their unique optical, electronic, and magnetic properties and potential applications in photonics, optoelectronics, and optical imaging.^28−34^ Among the various reported nanographenes,^35−40^ those with zigzag edges provide a particularly promising platform to explore electronic features such as narrow energy gaps and an open-shell biradical character.^41−44^ However, nanographenes with zigzag edges are often unstable under ambient conditions.^45,46^ Recently, Wu and co-workers reported a series of nanographenes with four zigzag edges and their integration into laser devices.^47^ We recently synthesized dibenzo[*hi*,*st*]ovalene (DBOV) derivatives with both zigzag and armchair edges, which exhibited high stability and strong red luminescence with fluorescence quantum yields of up to 97%.^48−54^ DBOV also displayed exceptional photophysical characteristics, such as intrinsic blinking in very different environments, superior fluorescence recovery, and stability over several months.^55^ These features make DBOV a unique fluorophore for optical super-resolution nanoscale imaging,^55^ although its hydrocarbon structure prohibits its use in sensing applications. Nanographene with some of its carbon atoms replaced by nitrogen atoms would be an excellent potential candidate for pH and metal-ion sensing, given the possibility of protonation of the nitrogen atom and its metal-ion chelating potential. Both are expected to modify the electronic structure and thereby the fluorescence properties of nanographene.^56,57^ According to the literature, the most probable fluorescence quenching mechanism of fluorophores with a nitrogen-incorporated structure is through protonation of the nitrogen atom and, for metal ions, photoinduced electron transfer (PET)^58^ upon coordination directly to metal ions. For a nitrogen-substituted nanographene, one may expect high proton/ion sensitivity owing to the nitrogen atom being part of the fluorescent aromatic core.

Nitrogen (N)-incorporation into PAHs has been extensively studied to tune their orbital energy levels, redox properties, and chemical reactivity.^59−67^ For example, aza derivatives of perylene,^59,60^ pyrene,^61^ coronene,^62,63^ and dibenzoperylene^64^ have been reported, demonstrating their *n*-type semiconductor character, acid-sensitive optical responses, and coordination to metal ions. N-containing heteroacenes with N-bearing zigzag edges have attracted considerable attention as active components of organic electronics, with repeated attempts to achieve higher N-heteroacenes.^65−67^ N incorporation into large PAHs, namely, nanographenes, has also been explored, thus providing models of N-doped graphene with increasing relevance to fundamental research and applications.^56,68−74^ For example, an electron-deficient N-containing hexa-*peri*-hexabenzocoronene (HBC) with fused pyridine rings,^56^ electron-rich hexapyrrolohexaazacoronenes,^73^ and an antiaromatic pyrazine-embedding HBC^74^ were reported, although the latter could be obtained only on a metal surface under ultra-high-vacuum conditions. However, nitrogen-doped nanographenes, especially those with N-bearing zigzag edges, are rare.^67^

In this work, we introduce nitrogen atoms into the zigzag edges of DBOV to establish 6,14-diazadibenzo[*hi*,*st*]ovalene (N-DBOV **10**) as a new N-doped nanographene. N-DBOV **10** presents unique opportunities for pH- and metal-ion sensing by fluorescence microscopy. N-DBOV **10** could be synthesized in eight steps with a total yield of 15% from commercially available starting material **1**. N-DBOV **10** displayed high photostability and lowered orbital energy levels, as revealed by cyclic voltammetry and density functional theory (DFT) calculations. Furthermore, spectroscopic characterizations demonstrate the pH- and metal-ion-sensitive behavior of N-DBOV **10**, showing quick quenching of the strong luminescence upon addition of acid or metal ions, such as Cu^2+^ and Fe^2+^. pH-dependent fluorescence blinking was also observed for N-DBOV **10**, indicating potential applications in nanoscale pH measurements in biological, environmental, and material research.

## Results and Discussion

### Synthesis of N-DBOV **10**

**Scheme 1:**
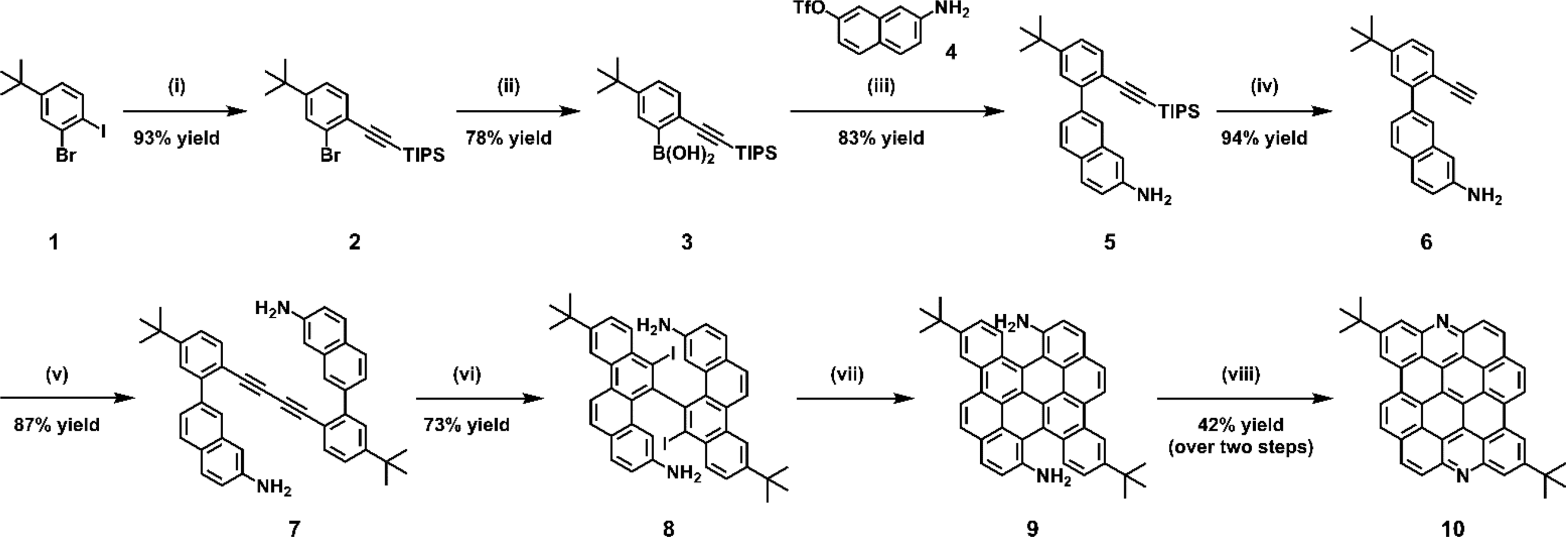
Synthesis of N-DBOV **10** Reagents and conditions: (i) Pd(PPh_3_)_2_Cl_2_ (0.02 equiv), CuI (0.04 equiv), THF/TEA (1:1), RT, 12 h; (ii) *n*-BuLi (1.2 equiv), THF, −78 °C, 2 h, B(O*i*Pr)_3_ (2.0 equiv), RT, overnight; (iii) Pd(PPh_3_)_4_ (0.05 equiv), K_2_CO_3_ (6.0 equiv), toluene/EtOH/H_2_O (4:1:1), 80 °C, overnight; (iv) TBAF (1.2 equiv), THF, RT, 3 h; (v) Cu(OAc)_2_ (1.0 equiv), MeOH/pyridine (1:1), 80 °C, overnight; (vi) ICl (2.1 equiv), DCM, −78 °C, 2 h; (vii) TEA (excess), toluene, hυ, 2 h; (viii) TBHP (2.5 equiv), KI (0.1 equiv), CH_3_CN/H_2_O, 60 °C. THF: tetrahydrofuran, TEA: trimethylamine, DCM: dichloromethane, TBHP: *tert*-butyl hydroperoxide.

The synthesis of N-DBOV **10** was carried out as shown in Scheme 1. The key intermediate **9**, with two amino groups, was prepared by adapting our previous procedure for another derivative with two formyl groups.^48^ 3-Bromo-4-triisopropylsilyl (TIPS)-ethynyl-*tert*-butylbenzene (**2**) was obtained though the Sonogashira reaction of 3-bromo-4-iodo-*tert*-butylbenzene (**1**) and TIPS-acetylene in 93% yield. Bromide **2** was lithiated with *n*-butyllithium (*n*-BuLi) and then reacted with triisopropyl borate to give 5-*tert*-butyl-2-(TIPS-ethynyl)phenylboronic acid (**3**) in 78% yield, which was subsequently subjected to Suzuki coupling with naphthyl triflate **4** to afford 7-{5-*tert*-butyl-2-(TIPS-ethynyl)phenyl}-2-naphthylamine (**5**) in 83% yield. After deprotection of **5** with tetra-*n*-butylammonium fluoride (TBAF) to provide **6** in 94% yield, a Cu-mediated Glaser coupling of **6** provided diaryldiacetylene **7** in 87% yield. Subsequently, iodination-benzannulation^75,76^ of **7** by treatment with ICl gave diiodobichrysenyl **8** in 73% yield. Photochemical cyclodehydroiodination^77^ of **8** in the presence of triethyl amine (TEA) provided fused product **9**, which was directly used for the next step. For the formation of the N-bearing zigzag edges, the use of the Cadogan reaction conditions^78^ was initially considered. When the oxidation of the amino groups of **9** to the nitro groups was attempted by treatment with *tert*-butyl hydroperoxide (TBHP) as the oxidant and KI as the catalyst,^79^ we found that N-DBOV **10** was directly formed instead in 42% yield over two steps. We assume that the amino group of **9** is activated by the *t*BuO radical, which is generated through the decomposition of TBHP mediated by KI,^80,81^ followed by intramolecular cyclization instead of further oxidation to the nitro group (see Scheme S1 for a possible mechanism). To the best of our knowledge, this is the first example of the direct oxidative cyclization of the amino group to form an N-incorporated PAH,^70,82,83^ which can potentially be useful for the synthesis of a wider variety of aza-PAHs.

**Figure 1:**
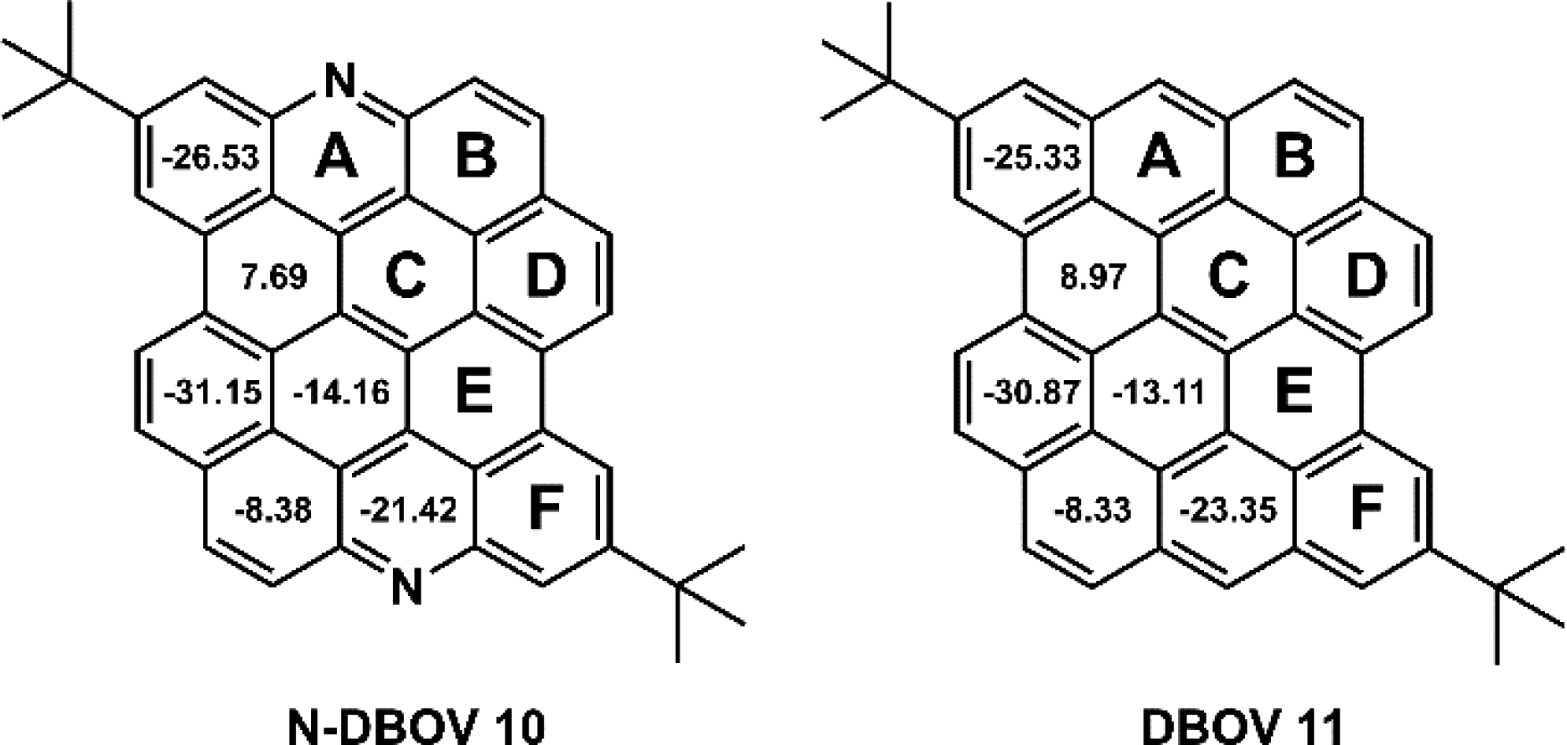
Chemical structures of N-DBOV **10** and DBOV **11**. The numbers inside the six-membered rings indicate NICS(1)_*zz*_ values.

Electron ionization high-resolution mass spectrometry (EI-HRMS) analysis of N-DBOV **10** revealed an intense signal at *m*/*z* = 587.2481, in agreement with the calculated mass of *m*/*z* = 587.2482 ([M + H]^+^). Limited solubility and strong intermolecular interactions of N-DBOV **10** hindered its NMR characterizations in its neutral form, while its ^1^H NMR spectrum could be recorded in trifluoroacetic acid (TFA)-*d* at 298 K as a dideuterated form, N-DBOV-2D^+^ (Figure S12), and all the proton resonances were assigned on the basis of their ^1^H,^1^H-correlation spectroscopy (COSY) and ^1^H,^1^H-nuclear Overhauser effect spectroscopy (NOESY) spectra (see the SI, Figures S1–S22). To determine the effect of N incorporation on the optical and electronic properties of the DBOV core, excluding the possible effects of alkyl or aryl substituents on the zigzag edges of previously reported DBOVs, a new DBOV derivative **11** was designed as a reference compound with bare zigzag edges and two *t*Bu groups in the same positions as those in **10** (Figure 1). The synthesis of **11** was carried out by adapting our previous procedure reported for other DBOV derivatives (see the SI for details).^48^

### Optical and Electronic Properties

**Figure 2:**
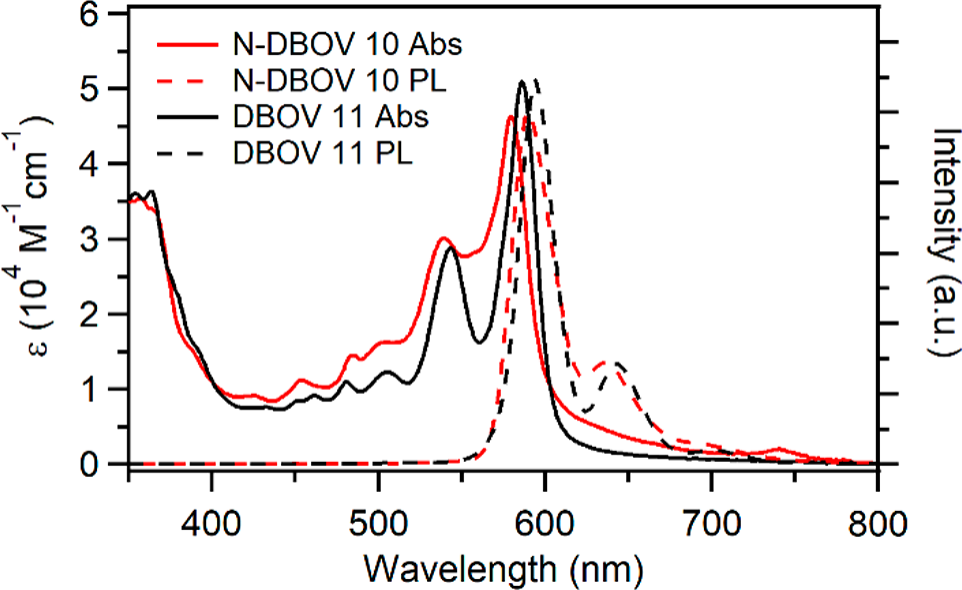
UV–vis absorption and photoluminescence (PL) spectra of 10^–6^ M solutions of N-DBOV **10** (red) and DBOV **11** (black) in THF measured at room temperature.

The UV–vis absorption spectra of N-DBOV **10** and DBOV **11** in tetrahydrofuran (THF) were very similar, exhibiting absorption maxima at 580 and 586 nm, respectively, with a vibronic progression at shorter wavelengths (Figure 2). These absorption bands were assigned to the HOMO–LUMO transitions based on time-dependent density functional theory (TD-DFT) calculations at the B3LYP/6-31G(d) level of theory (Figure S23 and Table 1).

**Table 1:** Optical and Electrochemical Properties of N-DBOV **10** and DBOV **11**

compound	λ_max_ (nm)	λ_em_ (nm)	ϕ^α^	τ (ns)	*E*_g_(opt) (eV)^a^	*E*_g_(cal) (eV)^b^	HOMO(cal) (eV)^b^	LUMO(cal) (eV)^b^	HOMO(CV) (eV)^c^
N-DBOV **10**	580	591	0.76	6.0	2.07	2.20	–4.99	–2.79	–4.83
DBOV **11**	586	594	0.80	7.5	2.05	2.13	–4.48	–2.35	–4.75

a Optical gaps were estimated based on the wavelengths at which the normalized absorption and fluorescence spectra cross each other.

b DFT calculations were performed at the B3LYP/6-31G(d) level of theory with the Gaussian 16 calculation package.^85^

c The HOMO energy levels were estimated by utilizing the onset of the first oxidation potential of CV calibrated with Fc/Fc^+^.

Similar to previously reported DBOV derivatives, N-DBOV **10** demonstrated strong red emission.^48,49^ Fluorescence spectra of N-DBOV **10** and DBOV **11** showed maxima at 588 and 594 nm, respectively, with Stokes shifts of 235 and 230 cm^–1^, respectively (Figure 2). The UV–vis and fluorescence spectra of **10** and **11** displayed more significant broadening compared with a previously reported DBOV derivative with two mesityl groups (Figure S24), which could be ascribed to the higher aggregation tendency of **10** and **11**. Nevertheless, shifts of their absorption and emission maxima were negligible in the concentration range of 10^–4^ to 10^–7^ M. The optical gaps of **10** and **11** were estimated to be 2.12 and 2.10 eV, respectively, from the wavelengths at which their normalized absorption and fluorescence spectra cross each other (Table 1). The fluorescence quantum yields of **10** and **11** were assessed using Nile blue A perchlorate as a standard to be 76% and 80%, respectively (Table 1). The absorption and fluorescence spectra of **10** displayed no significant variations (peak shifts below 8 nm) in different solvents with varying polarities, including toluene, THF, and dimethylformamide (Figure S25). These results indicated that N incorporation on the zigzag edges had a negligible effect on the static optical properties of the DBOV core. On the other hand, the fluorescence lifetime of N-DBOV **10** was determined to be 6.0 ns, which was slightly shorter than the value of 7.5 ns measured for DBOV **11** (Figure S26).

The electrochemical properties of N-DBOV **10** and DBOV **11** were characterized by cyclic voltammetry (CV) in anhydrous THF solutions at room temperature (Figure S27). In the CV curves of **10** and **11**, the onset of the first oxidation potentials occurred at 0.03 and −0.05 V, respectively, against Fc/Fc^+^, corresponding to HOMO energy levels of −4.83 and −4.75 eV, respectively, according to the equation HOMO = −(4.8 + *E*_ox_^onset^) (Table 1). This result indicated that N incorporation lowered the orbital energy level without significantly affecting the static optical properties discussed above, in line with previous reports for other N-bearing PAHs.^84^

**Figure 3:**
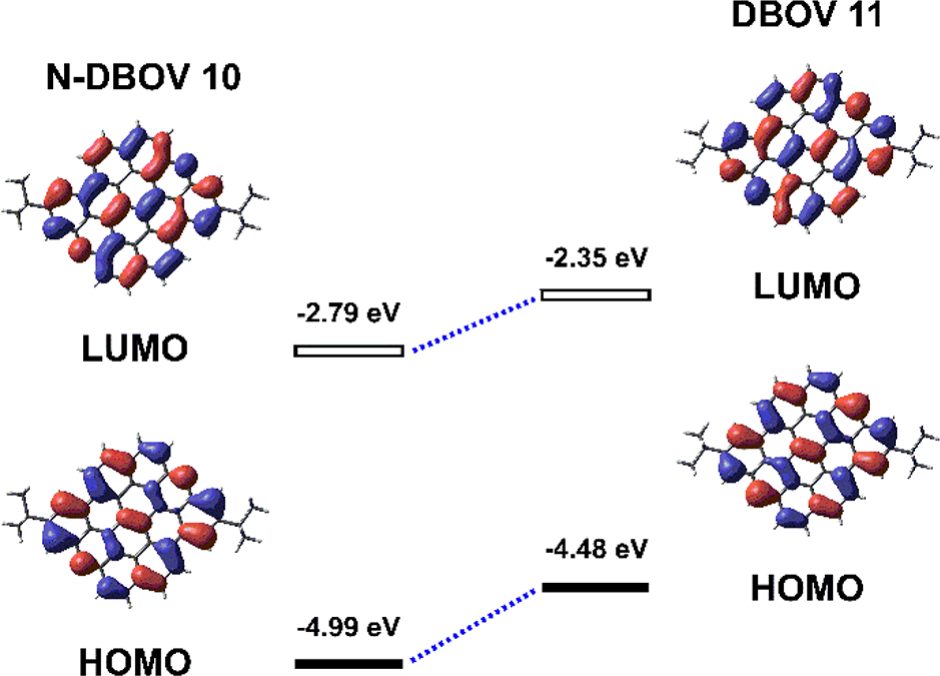
HOMOs and LUMOs of N-DBOV **10** and DBOV **11** calculated by DFT at the B3LYP/6-31G(d) level.

DFT calculations also revealed that N-DBOV **10** possesses lower-lying HOMO and LUMO energy levels (−4.99 and −2.79 eV) than DBOV **11** (−4.48 and −2.35 eV) but similar HOMO–LUMO energy gaps of 2.20 and 2.13 eV, respectively (Figure 3 and Table 1).

To examine the influence of N incorporation on the aromaticity of the DBOV core, nucleus-independent chemical shift (NICS) calculations were performed for N-DBOV **10** and DBOV **11** at the GIAO-B3LYP/6-31G(d) level of theory (Figure 1).^86,87^ The results for N-DBOV **10** and DBOV **11** were similar, indicating strong aromaticity in rings A, D, and F with negative NICS(1)_*zz*_ values ranging from −31.15 to −21.42. The anisotropies of the induced current density (ACID)^88^ plots of N-DBOV **10** and DBOV **11** were almost identical, displaying a clockwise (diatropic) ring current at an isosurface value of 0.05 (Figure S28).^51^ The NICS(1)_*zz*_ and ACID results indicate that N incorporation in the zigzag edges does not significantly affect the DBOV core aromaticity.

### Fluorescence Properties of N-DBOV

#### Fluorescence Stability

**Figure 4:**
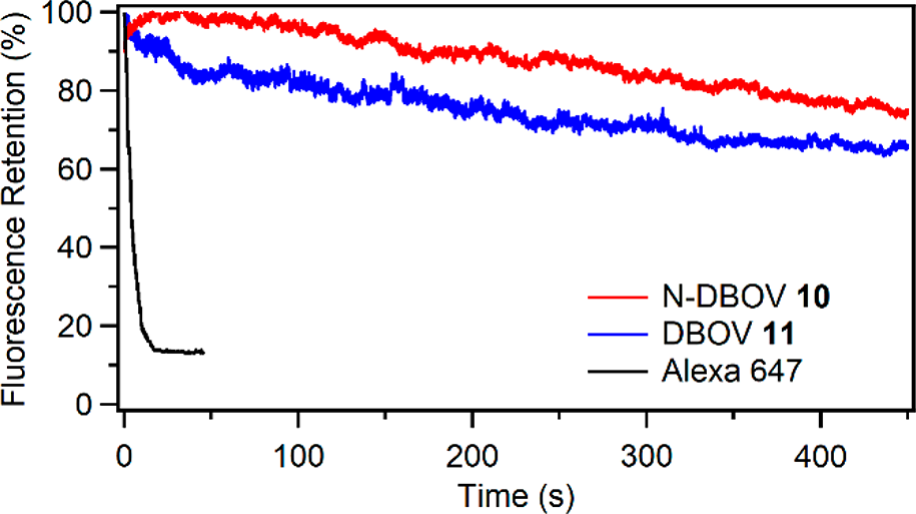
Photobleaching properties of N-DBOV **10** (red), DBOV **11** (blue), and Alexa 647 (black) as a function of the imaging time.

To examine the suitability of N-DBOV **10** for fluorescence imaging, we first compared the photostability of N-DBOV **10** with that of the commonly used organic dyes Alexa 647 and DBOV **11** deposited on glass coverslips (Figure 4).

To our delight, under the same continuous 532 nm laser excitation conditions in air, N-DBOV **10** maintained 74% of its fluorescence intensity after irradiation for up to 450 s, while Alexa 647 was quickly photobleached within 45 s (see the SI and Figure S29 for more details). DBOV **11** retained ∼66% of its fluorescence intensity for 450 s. Other dyes typically require antifading agents^21,36^ to improve their photostability. The high stability of N-DBOV allows its use in imaging applications with few constraints on its environment.

#### Protonation of N-DBOV **10**

**Figure 5:**
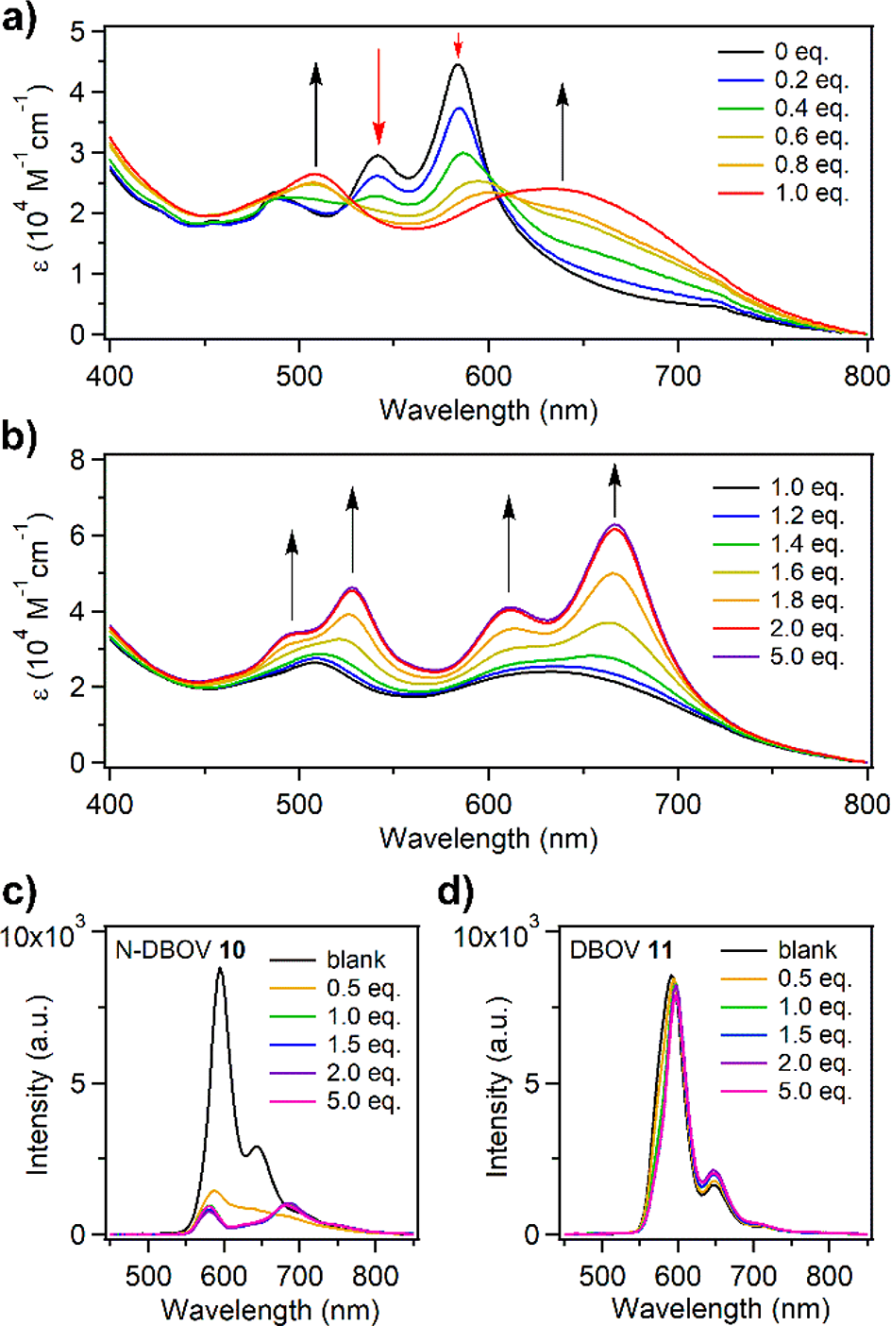
(a, b) Changes in the UV–vis absorption spectrum upon titration of N-DBOV **10** (2 × 10^–5^ M in DCM) with TFA, monitoring the protonation of (a) N-DBOV **10** to N-DBOV-H^+^ and (b) N-DBOV-H^+^ to N-DBOV-2H^+^. The arrows indicate the changes in the intensity of each peak. (c, d) Changes in the fluorescence spectra of (c) N-DBOV **10** and (d) DBOV **11** in DCM solution (2 × 10^–5^ M in DCM) upon successive addition of TFA, recorded at room temperature.

The acid sensitivity of N-DBOV **10** was investigated by monitoring the changes in UV–vis absorption while adding TFA. During the addition of up to 1.0 equiv of TFA to a solution of N-DBOV **10** in DCM, new broad absorption bands centered at 507 and 630 nm replaced the original peaks at 541 and 584 nm, resulting in isosbestic points at ∼526 and 609 nm (Figure 5a). The disappearance of the original N-DBOV absorption upon the addition of 1.0 equiv of TFA indicates complete conversion to N-DBOV-H^+^. Further addition of TFA up to 2.0 equiv increased the absorbance, with the appearance of well-resolved peaks at 498, 528, 612, and 667 nm (Figure 5b). An excess (5.0 equiv) of TFA did not change the spectrum, indicating that the formation of N-DBOV-2H^+^ was complete with 2.0 equiv of TFA. The acquired absorption spectra of N-DBOV-2H^+^ were consistent with those obtained from TD-DFT calculations (Figure S30). Protonation of N-DBOV **10** also strongly affects its fluorescent response, leading to the very efficient fluorescence quenching. The fluorescence of N-DBOV **10** could be largely quenched intensity, which was not observed for DBOV **11** by 0.5 equiv of TFA, while DBOV **11** did not exhibit any quenching behavior (Figure 5c and d). Protonation of N-DBOV **10** with HCl also significantly decreased the fluorescence of **11** (see Figures S31 and S32). The fluorescence of N-DBOV **10** could be fully recovered by subsequently adding triethylamine (Figure S33), indicating the proton-sensing ability of N-DBOV **10**. Based on the TD-DFT calculation, the fluorescence quenching of N-DBOV **10** under acidic conditions is attributed to a decrease in the radiative decay rate (Table S2).

#### Fluorescence Properties at the Single-Molecule Level

**Figure 6:**
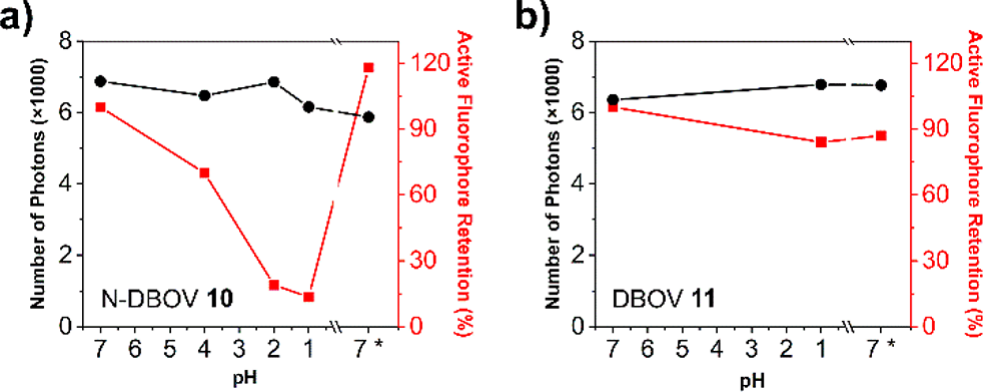
Results of single-molecule fluorescence measurements of (a) N-DBOV **10** and (b) DBOV **11** exposed to aqueous solutions with different pH values. Note the reversible pH dependence of the number of active emitters (red line) in only (a), while the fluorescence intensity for each active emitter (number of photons, black line) is retained.

The pH-dependent blinking properties of N-DBOV **10** and DBOV **11** were further analyzed at the single-molecule level (Figure 6 and Figure S34; see the SI for details). The number of active fluorophores and their numbers of emitted photons were determined as a function of pH. For these measurements, N-DBOV **10** molecules were first immobilized on a polystyrene-coated coverslip and then exposed to aqueous solutions with different pH values. The single-molecule fluorescence data are fully consistent with the ensemble measurements shown in Figure 5. Such single-molecule pH-dependent fluorescence could be used for super-resolution pH measurements.

As presented in Figure 6a, over 87% of the excited N-DBOV **10** molecules were quenched when the pH was decreased from 7 to 1, while the remaining molecules showed almost constant brightness. Meanwhile, DBOV **11** showed no significant pH-dependent fluorescence changes (Figure 6b).

After neutralizing the pH 1 solution by adding the same volume of an aqueous NaOH solution (0.1 M), the fluorescence of the quenched N-DBOV **10** molecules recovered, in agreement with the ensemble experiments. Therefore, N-DBOV **10** is stable under acidic conditions and sensitive to pH, making it attractive as a pH sensor with a low detection limit.

### Optical Imaging Applications

#### 3D Confocal Imaging

**Figure 7:**
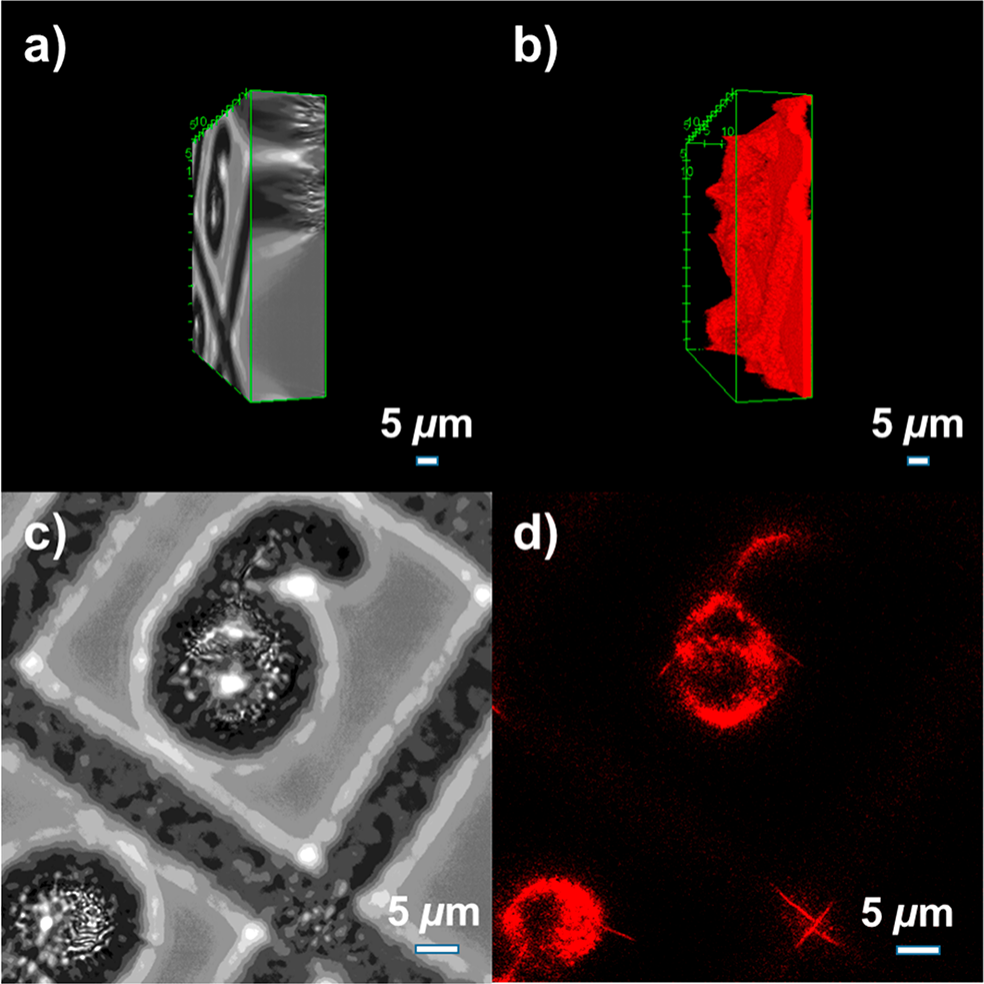
3D bright-field and confocal microscopy images of gridded structures in a glass substrate with N-DBOV **10**. Reconstructions of the (a) bright-field 3D image and (b) confocal fluorescence 3D image. (c) Bright-field image and (d) confocal fluorescence image, both at an imaging depth of 11.83 μm relative to the surface of the glass substrate.

To further assess the potential imaging applications of N-DBOV **10**, a 3D confocal imaging experiment was carried out. As shown in Figure 7, gridded structures in a glass substrate with a width and depth of 5 μm each (Ibidi, gridded glass coverslips, Grid-50) were imaged after deposition of N-DBOV **10** as the fluorescence probe. The sample was first imaged by conventional bright-field microscopy and then with confocal microscopy at each *z*-position. Along the *z*-direction, a series of 2D images on the *xy* plane were acquired with a step size of 0.13 μm.

Finally, 3D volumes were reconstructed with the image processing software ImageJ. From our 3D confocal microscopy imaging (Figure 7b and d), micro- and nanostructures with a depth up to 17 μm could be imaged, which are difficult to visualize with other techniques, such as bright-field microscopy (Figure 7a and c). N-DBOV **10** demonstrated excellent photostability during the whole process. All images were reconstructed from two averaged line scans, and two full 3D images were recorded sequentially, taking 16 min in total. The two 3D images showed that the fluorescence intensity was very stable, and no photobleaching was observed (see the SI, Figures S35–S36, Table S3, and Supporting Videos 1–4 for more details). We note here that realistic 3D confocal imaging can be achieved using a much shorter exposure time. The longer exposure time used here mainly served to demonstrate the photostability of N-DBOV **10**.

#### Optical Super-Resolution SMLM Imaging

For optical super-resolution SMLM imaging, blinking fluorescence with high photon numbers (detected photons per switching event) and low on–off duty cycle (fraction of time a molecule resides in its fluorescent state) are preferred.^23^ High photon numbers provide high imaging resolution, while a low on–off duty cycle could improve both the imaging accuracy and labeling density by decreasing the probability of two fluorophores fluorescing simultaneously within the diffraction-limited imaging area.^23^ However, the current gold-standard SMLM fluorophores, i.e., organic dyes in blinking buffer, typically degrade within hours.^89,90^ These boundary conditions increase the imaging complexity and limit the imaging environment and imaging time. Similar to its parent DBOV,^54^ the nanographenes presented here exhibited intrinsic blinking properties, independent of environment.

**Figure 8:**
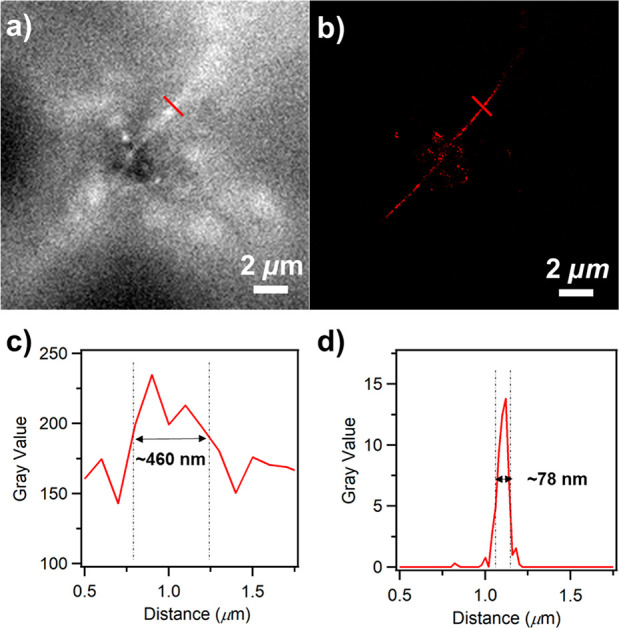
Optical fluorescence imaging of nanometer-sized crevices in a glass substrate with N-DBOV **10**. (a) Wide-field image and (b) super-resolution SMLM image. Intensity profiles of (c) the wide-field image and (d) the super-resolution SMLM image indicated using the red line shown in the images of (a) and (b), respectively.

Investigation of N-DBOV **10** by single-molecule fluorescence analysis^61^ demonstrated excellent blinking features, including high photon numbers of >6000 and low on–off-duty cycles of ∼10^–3^ with a blinking time of approximately 100 ms, in different environments, including in air, embedded in a polystyrene (PS) film, and in water (see the SI and Figure S37 for details about sample preparation and measurements). These results indicated the excellent suitability of N-DBOV **10** for optical super-resolution SMLM imaging. As a proof of concept, we performed an SMLM experiment using N-DBOV **10** to image nanoscale crevices in a glass substrate. Figure 8 shows a direct comparison of the imaging results from conventional wide-field (WF) microscopy (Figure 8a) and the SMLM method (Figure 8b).

In the SMLM imaging, N-DBOV **10** molecules could be localized with an average localization precision of 21 nm. Compared to that of the WF image, the resolution of the SMLM image was significantly enhanced, with the SMLM method yielding an ∼6-fold reduction in the measured crevice width (Figure 8c and d). We note here that unlike other fluorophores commonly used for the SMLM method, which always require blinking buffer,^23^ N-DBOV **10** enabled SMLM imaging in air without blinking buffer (see the SI for more details about the sample preparation and SMLM imaging analysis).

#### Metal-Ion Sensitivity

**Figure 9:**
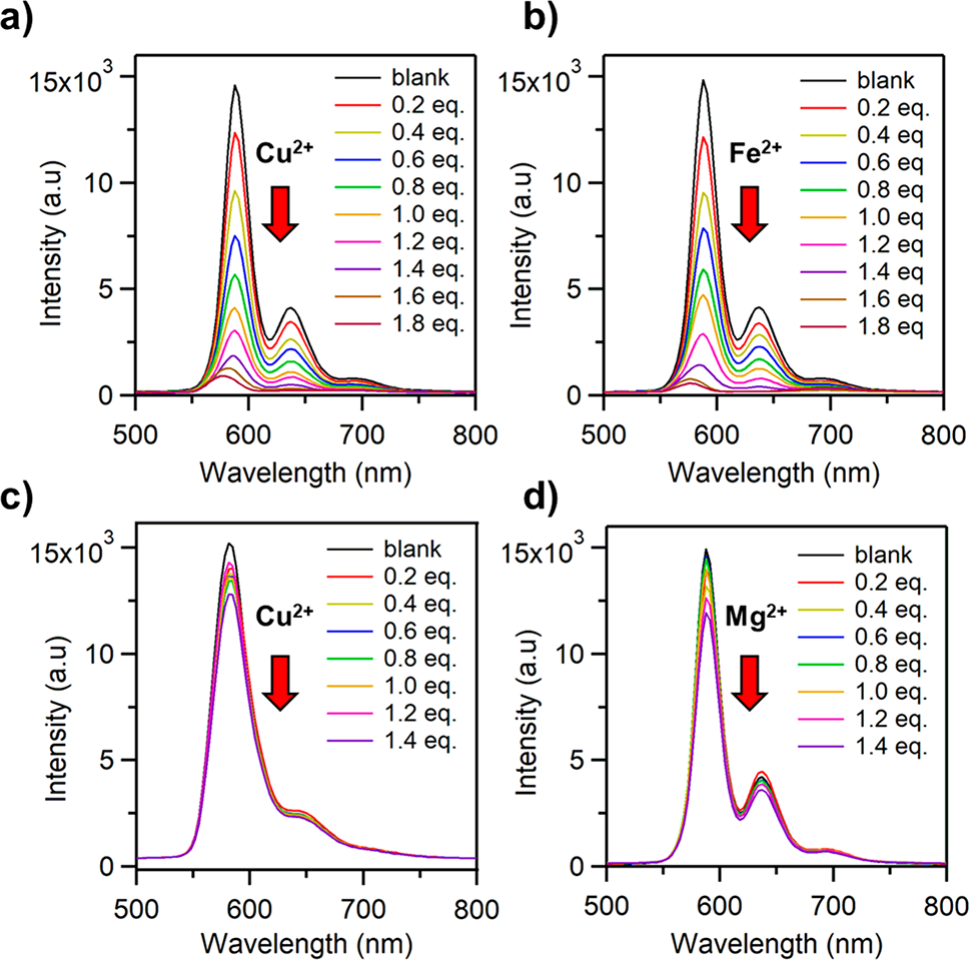
Changes in the fluorescence spectrum of 5 × 10^–5^ M N-DBOV **10** measured in THF solution at room temperature upon successive addition of (a) Cu^2+^, (b) Fe^2+^, and (d) Mg^2+^ ions; changes in the fluorescence spectrum of DBOV **11** upon gradual addition of (c) Cu^2+^ ions.

To investigate the ion-sensing capability of N-DBOV **10**, its fluorescence response to different metal ions was examined. To this end, 9 × 10^–4^ M Cu^2+^, Fe^2+^, and Mg^2+^ solutions were prepared by dissolving Cu(ClO_4_)_2_·6H_2_O, Fe(ClO_4_)_2_, and Mg(ClO_4_)_2_, respectively, in THF. The resulting metal-ion solutions were gradually added to 5 × 10^–5^ M solutions of N-DBOV **10** or DBOV **11** in THF. Figure 9a and b reveal a significant decrease in the fluorescence intensity of N-DBOV **10** upon successive addition of Cu^2+^ (0–1.8 equiv) and Fe^2+^ (0–1.8 equiv) ions at room temperature. Indeed, N-DBOV **10** is highly sensitive to Cu^2+^ and Fe^2+^ ions, with a detection limit of 1 × 10^–5^ M, while DBOV **11** shows a much lower sensitivity (Figure 9c). According to the Stern–Volmer plot (Figure S38),^91,92^ the quenching constant (*K*_SV_) of N-DBOV **10** was determined to be 3.4× 10^6^ M^–1^ for Cu^2+^ detection and 3.3 × 10^6^ M^–1^ for Fe^2+^ detection. In contrast to Cu^2+^ and Fe^2+^, the addition of Mg^2+^ ions did not significantly decrease the fluorescence intensity of N-DBOV **10** (Figure 9d), consistent with the quenching being caused by the interaction of metal ions with the N atoms of N-DBOV **10** through intermolecular charge transfer.^93^ Therefore, N-DBOV **10** promises to be a selective sensor material for heavy metal ions with unique fluorescence properties that enable single-molecule imaging.

## Conclusion

In summary, we have introduced N-DBOV **10** as a novel nanographene with nitrogen atoms incorporated into the zigzag edges; N-DBOV **10** exhibited a narrow energy gap and brilliant red luminescence with a high photoluminescence quantum yield of 76%. By virtue of nitrogen incorporation, N-DBOV **10** sensitively responded to protons as well as Cu^2+^ and Fe^2+^ ions, as indicated by clear changes in its absorption and/or fluorescence spectra. Moreover, N-DBOV **10** has excellent photophysical properties, such as high photostability and intrinsic blinking, which are beneficial for long-term 3D fluorescence imaging and super-resolution SMLM imaging, respectively. The combination of SMLM with the pH-dependent blinking properties of N-DBOV **10** can provide a way to determine pH differences at the nanoscale. Moreover, introduction of hydrophilic groups at the peripheral positions can potentially make N-DBOV **10** water-soluble^33,94^ and thus provide very promising dyes not only in material analysis but also in biological systems. As such, purpose-oriented derivatization of DBOVs, for example, installment of binding functionality for biotargeting, can lead to new opportunities in modern imaging applications, which is currently pursued in our laboratories.

## Supporting Information Available

Experimental details, synthesis and characterizations of new compounds, NMR spectra, and computational details (PDF)
Movie S1 (AVI)
Movie S2 (AVI)
Movie S3 (AVI)
Movie S4 (AVI)

## Supplementary Material

ja1c04880_si_001.pdf

ja1c04880_si_002.avi

ja1c04880_si_003.avi

ja1c04880_si_004.avi

ja1c04880_si_005.avi
