# Supplementary material for: A Highly Luminescent Nitrogen-Doped Nanographene as an Acid- and Metal-Sensitive Fluorophore for Optical Imaging
Source: J Am Chem Soc. 2021 Jul 5;143(27):10403–12. doi: 10.1021/jacs.1c04880 (PMC8283754; doi:10.1021/jacs.1c04880)
Supplement: Supplementary file 1 — ja1c04880_si_001.pdf [file ja1c04880_si_001.pdf]

Supplementary Information

for

A Highly Luminescent Nitrogen-Doped Nanographene as an Acid- and Metal-Sensitive Fluorophore for Optical Imaging

Enquan Jin,[+,†] Qiqi Yang,[+,†] Cheng-Wei Ju,[†,⊥] Qiang Chen,[†] Katharina Landfester,[†] Mischa Bonn,[*,†] Klaus Müllen,[*,†,‡] Xiaomin Liu,[*,†]Akimitsu Narita[*,†,§]

[†]Max Planck Institute for Polymer Research, Ackermannweg 10, Mainz 55128, Germany

[‡]Institute of Physical Chemistry, Johannes Gutenberg-University, Duesbergweg 10-14, Mainz 55128, Germany

[§]Organic and Carbon Nanomaterials Unit, Okinawa Institute of Science and Technology Graduate University, Okinawa 904-0495, Japan

[⊥]College of Chemistry, Nankai University, Tianjin 300071, China

[+]These authors contributed equally to this work.

Correspondence and requests for materials should be addressed to M.B. (email: bonn@mpip-mainz.mpg.de), K.M. (email: muellen@mpip-mainz.mpg.de), X.L. (liuxiaomin@mpip-mainz.mpg.de) and A.N. (email: akimitsu.narita@oist.jp).

Table of Content

General Methods.. S3

Synthetic Details.. S4

Imaging and Sensor Details.. S14

Supplementary Figures and Tables.. S17

Appendix.. S38

References.. S43

General Methods

All reactions working with air- or moisture- sensitive chemicals were performed under Ar atmosphere with Schlenk line techniques. Unless otherwise noted, all reagents (including MICRO 90® concentrated solution) were purchased from commercial sources and used without further purification. Thin layer chromatography (TLC) was done on silica gel coated aluminum sheets with F254 indicator and column chromatography separation was performed with silica gel (particle size 0.063–0.200 mm). Nuclear Magnetic Resonance (NMR) spectra were recorded using Bruker DPX 300, and Bruker DRX 500 MHz NMR spectrometers. Chemical shifts (δ) were expressed in ppm relative to the residual of solvents (CD_2Cl_2, ^{1}H: 5.32 ppm, ^{13}C: 53.84 ppm; $C_2D_2Cl_4$, ^{1}H: 6.00 ppm, ^{13}C: 73.78 ppm; tetrahydrofuran (THF)-d_8, ^{1}H: 3.58 ppm, ^{13}C: 67.57 ppm; *o*-dichlorobenzene (*o*-DCB)-d_4, ^{1}H: 7.20 ppm, ^{13}C: 132.35 ppm). Coupling constants (J) were recorded in Hertz. High-resolution mass spectrometry (HR MS) was performed on a SYNAPT G2-Si high resolution time-of-flight mass spectrometer (Waters Corp., Manchester, UK) by matrix-assisted laser desorption/ionization (MALDI) using 7,7,8,8-tetracyanoquinodimethane (TCNQ) as matrix calibrated against poly(ethylene glycol) and electron ionization (Bruker MicroTOF-QII LCMS). UV-vis absorption spectra were recorded on a Perkin-Elmer Lambda 900 spectrometer at room temperature using a 10 mm quartz cell. Photoluminescence spectra were recorded on a J&MTIDAS spectrofluorometer. The fluorescence quantum yield (Φ) was measured using nile blue A perchlorate (in ethanol under air, Φ = 0.27) as a reference.[1] Cyclic voltammetry (CV) measurements were performed on a GSTAT-12 in a three-electrode cell in *o*-DCB solution of *n*-Bu_4NPF_6 (0.1 M) at a scan rate of 50 mV/s at room temperature. A silver wire, a Pt wire and a glassy carbon electrode were used as the reference electrode, the counter electrode, and the working electrode, respectively. Density functional theory (DFT) calculations were conducted with using Gaussian 16 software package.[2] Geometry optimizations at ground state were performed wtih the B3LYP functional and 6-31G(d) basis set. All the optimized structures were verified as energy minima via frequency calculations. Theoretical UV-vis absorption spectra were calculated through time-dependent density functional theory (TD-DFT) method at B3LYP/6-31G(d) level. The excited state geometries were optimized at the TD-M06-2X/6-31G(d)/PCM(THF) level of theory to simulate the fluorescence spectra**.**

The anisotropy of the induced current density (ACID) plots were obtained using Herges' method.[3] Multiwfn[4] was used to perform topological analysis to find the atoms-in-molecules

(AIM) ring critical points (RCPs) and then the spatial positions where the nucleus-independent chemical shift (NICS) calculations were carried out.[5, 6] The NICS(1)$_{ZZ}$ values were obtained using the standard gauge invariant atomic orbital (GIAO) method at the B3LYP/6-31G(d) level of theory.

Synthetic Details

The starting compounds **1** and **4** were prepared using previously reported methods.[7, 8]

3-bromo-4-[(triisopropylsilyl)ethynyl]-*tert*-butylbenzene (**2**)

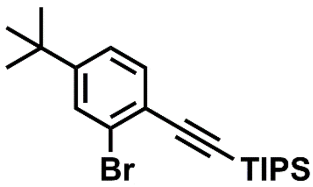

To a degassed solution of 3-bromo-4-iodo-*tert*-butylbenzene (**1**) (15.0 g, 44.3 mmol) and CuI (337 mg, 1.77 mmol) in THF (120 mL) and triethylamine (TEA) (120 mL) was added $Pd(PPh_3)_2Cl_2$ (621 mg, 0.885 mmol). Then, (triisopropylsilyl)acetylene (8.88 g, 48.7 mmol) was injected via a syringe. The reaction mixture was stirred at room temperature overnight. Then the reaction mixture was diluted with diethyl ether (200 mL), washed with water (100 mL) and brine (100 mL) and dried over with $MgSO_4$. The solvent was removed under reduced pressure and the residue was purified by column chromatography over silica gel (*n*-hexane) to give compound **2** (16.2 g, 93% yield) as colorless oil. TLC R_f = 0.8 (*n*-hexane); ^{1}H NMR (300 MHz, CD_2Cl_2, 25 °C, ppm) δ 7.64 (d, J = 1.9 Hz, 1H), 7.48 (d, J = 8.2 Hz, 1H), 7.33 (dd, J = 8.2, 1.9 Hz, 1H), 1.33 (s, 9H), 1.22 – 1.09 (m, 21H). ^{13}C NMR (75 MHz, CD_2Cl_2, 25 °C, ppm) δ 154.0, 133.7, 129.9, 125.7, 124.7, 122.9, 105.3, 95.5, 35.2, 31.1, 18.8, 11.7; HR MS (MALDI-TOF): *m/z* Calcd for $C_{21}H_{33}BrSi$: 392.1535 $[M]^+$, found: 392.1523.

[5-*tert*-butyl-2-(triisopropylsilylethynyl)phenyl]boronic acid (**3**)

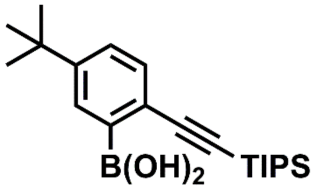

To a solution of 3-bromo-4-[(triisopropylsilyl)ethynyl]-*tert*-butylbenzene (16.0 g, 40.7 mmol) in THF (200 mL) was added dropwise *n*-BuLi (30.5 mL, 1.6 M in hexane) at –78 °C. After

stirring at –78 °C for 2 h, triisopropyl borate (14.5 g, 77.3 mmol) was added via a syringe. The mixture was gradually warmed to room temperature and was quenched with 1 N HCl (100 mL) and stirred for 0.5 h. The mixture was extracted with diethyl ether (200 mL) for 3 times. The organic layers were combined, washed with water (100 mL) and dried over $MgSO_4$. The solvent was removed under reduced pressure and the residue was purified by column chromatography over silica gel (*n*-hexane/ethyl acetate (EA) = 10 : 1) to give compound **3** (11.4 g, 78% yield) as colorless oil. TLC R_f = 0.2 (*n*-hexane/EA = 10 : 1); ^{1}H NMR (250 MHz, CD_2Cl_2, 25 °C, ppm) δ 8.04 (s, 1H), 7.55 – 7.48 (m, 2H), 5.94 (s, 2H), 1.37 (s, 9H), 1.21 – 1.16 (m, 21H); ^{13}C NMR (125 MHz, CD_2Cl_2, 25 °C, ppm) δ 151.6, 132.9, 132.2, 127.9, 123.9, 108.6, 95.0, 34.7, 30.8, 18.3, 11.3; HR MS (MALDI-TOF): *m/z* Calcd for $C_{21}H_{35}BO_2Si$: 358.2499 $[M]^+$, found: 358.2488.

7-[5-*tert*-butyl-2-(triisopropylsilylethynyl)phenyl]-2-naphthylamine (**5**)

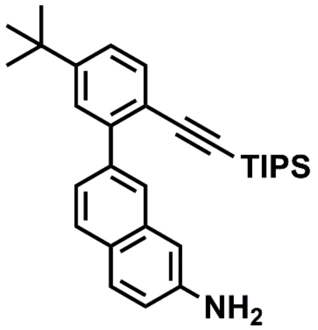

To a degassed solution of compound **3** (10.0 g, 27.9 mmol), compound **4** (8.13 g, 27.9 mmol) and K_2CO_3 (23.1 g, 167 mmol) in toluene/EtOH/H_2O (160 mL/40 mL/40 mL), $Pd(PPh_3)_4$ (1.61 g, 1.40 mmol) was added. The mixture was heated at 80 °C overnight. Then the reaction mixture was cooled to room temperature and extracted with diethyl ether (150 mL) for three times. The combined organic layers were dried over $MgSO_4$. The solvents were removed under reduced pressure and the residue was purified by column chromatography (*n*-hexane/EA = 5 : 1) to give compound **5** (10.6 g, 83% yield) as a white solid. TLC R_f = 0.3 (*n*-hexane/EA = 5 : 1); ^{1}H NMR (250 MHz, CD_2Cl_2, 25 °C, ppm) δ 7.74 (s, 1H), 7.65 (t, J = 8.1 Hz, 2H), 7.53 (d, J = 8.1 Hz, 1H), 7.46 (d, J = 8.7 Hz, 2H), 7.32 (d, J = 8.2 Hz, 1H), 6.96 (s, 1H), 6.93 (d, J = 8.6 Hz, 1H), 3.89 (s, 1H), 1.33 (s, 9H), 0.94 (s, 21H); ^{13}C NMR (75 MHz, CD_2Cl_2, 25 °C, ppm) δ 152.4, 145.0, 144.4, 139.4, 135.1, 133.8, 129.1, 127.5, 127.4, 127.2, 126.3, 124.7, 124.4, 119.4, 118.6, 108.9, 107.1, 93.4, 35.2, 31.3, 18.7, 11.6; HR MS (MALDI-TOF): *m/z* Calcd for $C_{31}H_{41}NSi$: 455.3008 $[M]^+$, found: 455.3005.

7-[5-(*tert*-butyl)-2-ethynylphenyl]-2-naphthylamine (**6**)

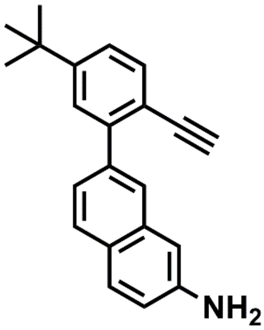

To a solution of compound **5** (9.00 g, 19.7 mmol) in anhydrous THF (150 mL) was added tetra-*n*-butylammonium fluoride (TBAF) (23.6 mmol, 23.6 mL, 1.00 M in THF) dropwise, and the reaction mixture was stirred at room temperature for 3 h. After completion of the reaction, MeOH (10 mL) was added followed by stirring for 30 min. Then the mixture was diluted with diethyl ether (200 mL), washed with water (150 mL) and dried over $MgSO_4$. The solvents were removed under reduced pressure and the residue was purified by silica gel column chromatography (*n*-hexane/EA = 5 : 1) to yield compound **6** (5.54 g, 94%) as white solid. TLC R_f = 0.3 (*n*-hexane/EA = 5 : 1); ^{1}H NMR (300 MHz, CD_2Cl_2) δ 7.79 (s, 1H), 7.76 (d, J = 8.5 Hz, 1H), 7.72 (d, J = 8.9 Hz, 1H), 7.60 (d, J = 8.2 Hz, 1H), 7.52 (s, 1H), 7.48 (d, J = 8.3 Hz, 1H), 7.41 (d, J = 8.1 Hz, 1H), 3.98 (s, 1H), 3.07 (s, 1H), 1.39 (s, 1H); ^{13}C NMR (75 MHz, CD_2Cl_2) δ 152.9, 145.3, 144.7, 139.1, 135.1, 133.9, 129.2, 127.5, 127.3, 126.4, 124.6, 124.4, 118.8, 118.6, 117.9, 108.7, 83.7, 79.6, 35.2, 31.3; HR MS (MALDI-TOF): *m/z* Calcd for $C_{22}H_{21}N$: 299.1674 $[M]^+$, found: 299.1676.

1,4-bis{2-(7-aminonaphthalen-2-yl)-4-*tert*-butylphenyl}buta1,3-diyne (**7**)

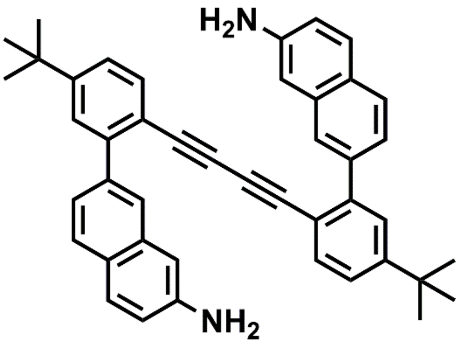

To a degassed solution of compound **6** (4.00 g, 13.4 mmol) in MeOH/pyridine (150 mL/150mL) was added $Cu(OAc)_2$ (2.43 g, 13.4 mmol). The reaction mixture was heated at 80 °C under argon overnight. After completion of the reaction, the reaction mixture was diluted with water (250 mL). After filtration, the residual solid was washed with water (100 mL) three

times and EtOH (100 mL) three times, and then dried under vaccum for 12 h. The solid mixture was further purified by column chromatography over silica gel (*n*-hexane/EA = 3 : 1) to give compound **7** (3.48 g, 87% yield) as a white solid. TLC R_f = 0.3 (*n*-hexane/EA = 4 : 1); ^{1}H NMR (300 MHz, CD_2Cl_2, 25 °C, ppm) δ 7.65 (s, 2H), 7.60 (d, J = 3.7 Hz, 2H), 7.57 (d, J = 3.2 Hz, 2H), 7.45 (d, J = 9.7 Hz, 2H), 7.40 (s, 2H), 7.34 (d, J = 10.4 Hz, 4H), 7.26 (d, J = 9.7 Hz, 2H), 6.90 (d, J = 6.9 Hz, 4H), 3.82 (s, 4H), 1.26 (s, 18H); ^{13}C NMR (75 MHz, $C_2D_2Cl_4$, 25 °C, ppm) δ 152.9, 144.6, 144.5, 138.5, 134.6, 134.2, 129.0, 127.4, 127.1, 127.0, 126.0, 124.4, 124.2, 118.7, 117.2, 108.9, 81.8, 76.6, 35.0, 31.2; HR MS (MALDI-TOF): *m/z* Calcd for $C_{44}H_{40}N_2$: 596.3191 [M]$^+$, found: 596.3185.

9,9'-di-*tert*-butyl-6,6'-diiodo-[5,5'-bichrysene]-3,3'-diamine (**8**)

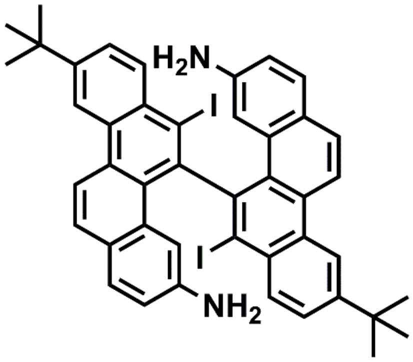

To a degassed solution of compound **7** (2.00 g, 3.35 mmol) dissolved in anhydrous dichloromethane (DCM) (200 mL) was added ICl (7.04 mL, 1.0 M in DCM) at −78 °C. Then the mixture was gradually warmed to room temperature. After stirring at room temperature overnight, excess ICl was quenched with a saturated aqueous solution of Na_2SO_3 (150 mL). The organic phase was extracted with DCM (150 mL) three times and dried over $MgSO_4$. The solvents were removed under reduced pressure. The residual solid was purified with column chromatography over silica gel (*n*-hexane/ethyl acetate (EA) = 3 : 1) to give compound **8** (2.08 g, 73% yield) as a red solid. TLC R_f = 0.3 (*n*-hexane/EA = 5 : 1); ^{1}H NMR (250 MHz, CD_2Cl_2, 25 °C, ppm) δ 9.34 (d, J = 9.5 Hz, 2H), 9.25 (s, 2H), 9.02 (s, 2H), 8.42 (d, J = 8.9 Hz, 2H), 8.34 (d, J = 9.2 Hz, 2H), 8.11 (d, J = 1.4 Hz, 4H), 7.93 (dd, J = 8.9, 1.9 Hz, 2H), 1.65 (s, 18H). Well-resolved ^{13}C NMR spectrum could not be recorded due to limited solubility. HR MS (MALDI-TOF): *m/z* Calcd for $C_{44}H_{38}I_2N_2$: 848.1124 [M]$^+$, found: 848.1118.

2,11-*tert*-butyl-5,14-diaminobenzo[*a*]dinaphtho[2,1,8-*cde*:1',2',3',4'-*ghi*]perylene (**9**)

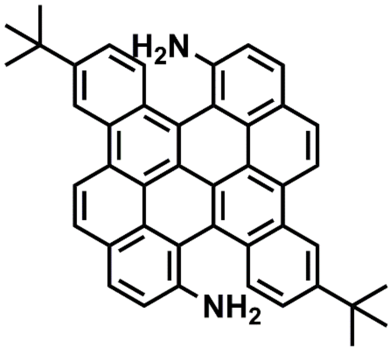

To a 3-L cylindrical quartz reactor, a solution of 9,9'-di-*tert*-butyl-6,6'-diiodo-[5,5'-bichrysene]-3,3'-diamine (**8**) (400 mg, 0.471 mmol) dissolved in a mixture of toluene (800 mL) and TEA (8 mL) was added. The solution was degassed by argon bubbling for 10 min, and vigorously stirred at room temperature in a photoreactor (RAYONET RPR-200 photochemical reactor) for 3 h under irradiation by sixteen UV lamps (300 nm, 14 W). After completion of the photoreaction, the solvents were removed in vacuo and the residue was purified by column chromatography over aluminum oxide (*n*-hexane:THF = 1 : 2) to obtain crude product of compound **9** as a red solid (221 mg), which was used for the next step without further purification. HR MS (MALDI-TOF): *m/z* Calcd for $C_{44}H_{36}N_2$: 592.2878 $[M]^+$, found: 592.2869.

6,14-diazadibenzo[*hi*,*st*]ovalene (**10**)

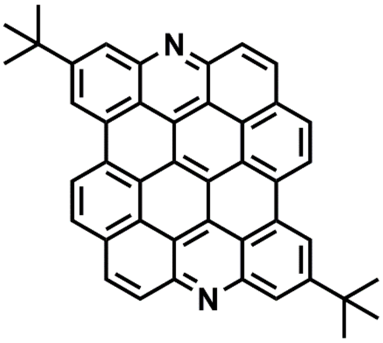

To a degassed solution of crude compound **7** (150 mg, 0.253 mmol) and KI (4.2 mg, 0.025 mmol) in CH_3CN (10 mL) was added *tert*-butyl hydroperoxide (70% solution in H_2O, 81.4 mg). Then the mixture was heated at 80 °C overnight. After cooling to room temperature, the mixture was quenched with a saturated aqueous solution of Na_2SO_3 (30 mL). The mixture was filtered and washed with water (20 mL) three times and MeOH (20 mL) three times. The residue was dried under vacuum to give analytically pure compound **10** (78.7 mg, 42% yield over two steps) as a purple solid. ^{1}H NMR (500 MHz, trifluoroacetic acid (TFA)-d_1 and inner small tube with THF-d_8 for lock) δ 10.23 (d, J = 8.2 Hz, 2H), 10.11 (s, 2H), 9.56 (d, J = 8.2 Hz, 2H), 9.50 (d, J

= 8.7 Hz, 2H), 9.17 (s, 2H), 9.02 (d, J = 8.9 Hz, 2H), 2.06 (s, 18H); Well-resolved ^{13}C NMR spectrum could not be recorded due to limited solubility. HR MS (EI): *m/z* Calcd for $C_{44}H_{30}N_2H$: 587.2482 $[M+H]^+$, found $[M+H]^+$: 587.2481.

Scheme S1. Proposed mechanism of oxidative step of compound **9.**

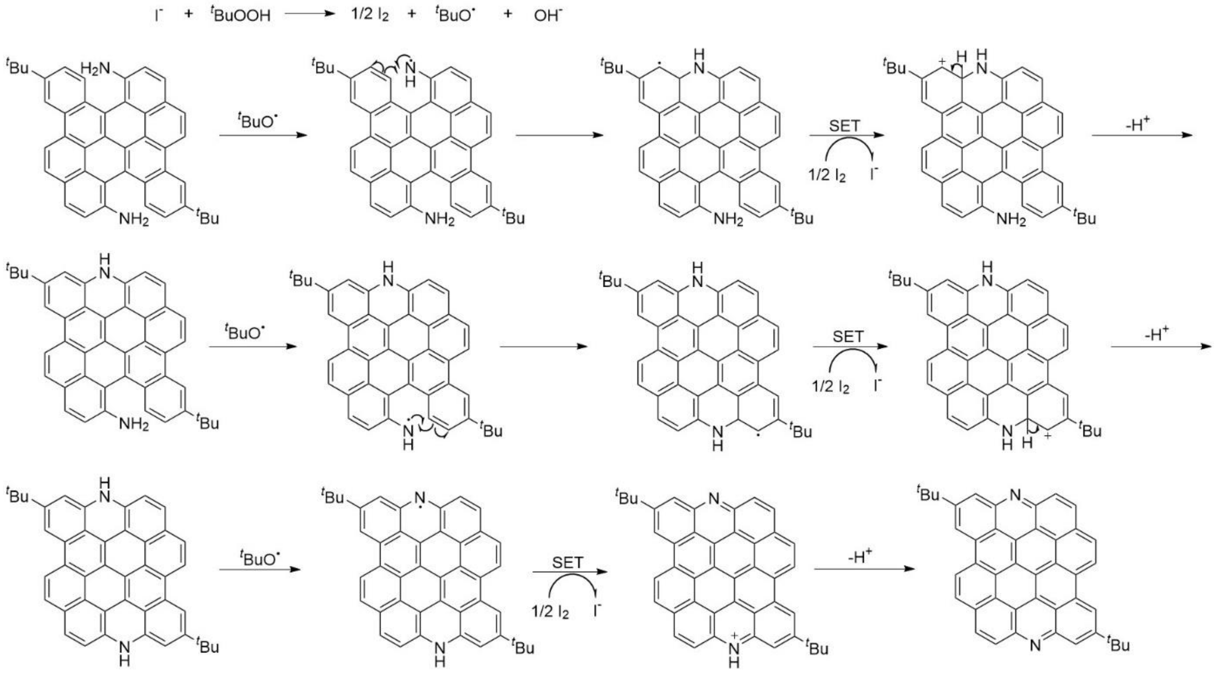

For the synthesis of DBOV **11**, precursor **S6** was prepared as shown in Scheme S1, adapting a procedure that we have previously reported.[9, 10] Photochemical reaction of **S6** in toluene directly provided DBOV **11**, presumably because HI generated upon the photochemical cyclodehydroiodination subsequently promoted the intramolecular Friedel–Crafts reaction. In our previous report, triethylamine was used as a co-solvent to trap the generated HI.[10] Although the low solubility and strong aggregation tendency of **11** did not allow its characterization by NMR analyses, agreement of its UV-vis and photoluminescence spectra with those of previously reported DBOV derivatives (see Figure S24), in addition to the high-resolution mass spectrometry analysis, validated the formation of **11**.

Scheme S2. Synthetic route towards DBOV **11.**

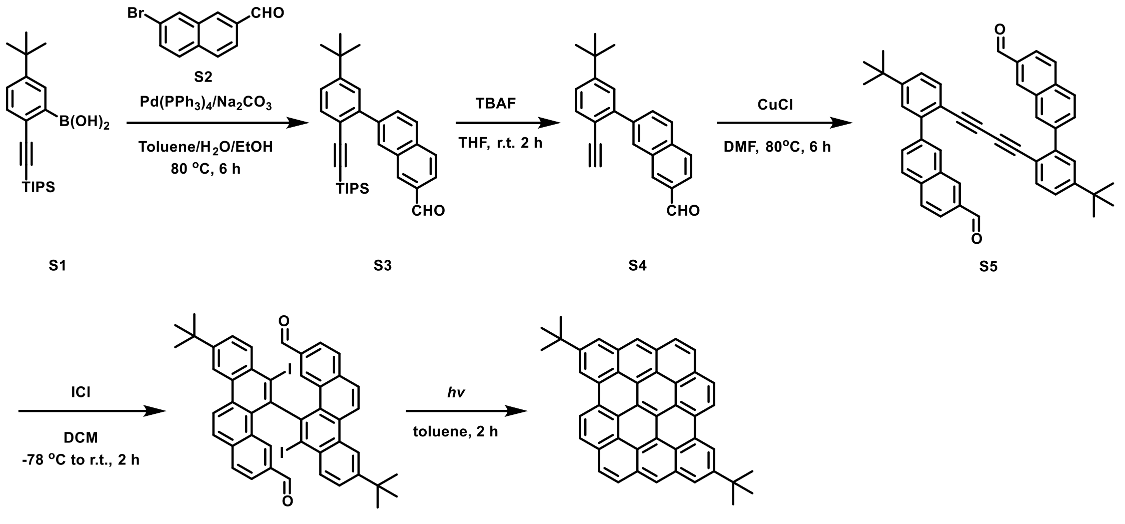

7-(5-*tert*-butyl-2-triisopropylsilylethynylphenyl)-2-naphthaldehyde (**S3**)

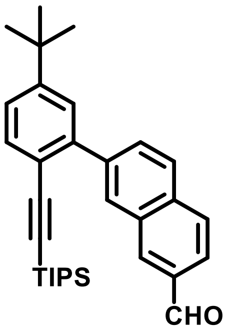

To a Schlenk flask were added (5-*tert*-butyl-2-triisopropylsilylethynylphenyl)boronic acid (**S1)** (13.1 g, 36.6 mmol), 7-bromo-2-naphthaldehyde **2** (8.17 g, 34.8 mmol), and Na_2CO_3 (19.4 g, 183 mmol). The flask was evacuated and backfilled with Ar for 3 times. Then a mixture of toluene, EtOH, and H_2O (280 mL/70 mL/70 mL) was added via a syringe. After degassing by bubbling with Ar for 15 min, $Pd(PPh_3)_4$ (2.12 g, 1.83 mmol) was added in one portion. The mixture was then heated at 80 °C for 6 h under Ar atmosphere. The reaction mixture was then cooled down to room temperature and extracted with EA (200 mL) for 3 times. The combined organic layers were washed with brine (200 mL), dried over Na_2SO_4, and evaporated. The residue was purified by silica gel column chromatography (*n*-hexane/EA = 10 : 1) to give the title compound (14.0g, 82% yield) as colorless oil. TLC R_f = 0.5 (*n*-hexane/EA = 10 : 1); ^{1}H NMR (250 MHz, CD_2Cl_2) δ 10.16 (s, 1H), 8.38 (s, 1H), 8.23 (s, 1H), 8.04 – 7.88 (m, 4H), 7.60 (d, J = 8.2 Hz, 1H), 7.51 (s, 1H), 7.41 (d, J = 8.1 Hz, 1H), 1.38 (s, 9H), 0.93 (s, 21H); ^{13}C NMR

(63 MHz, CD_2Cl_2) δ 192.46, 152.70, 143.39, 140.47, 135.99, 135.18, 134.83, 133.90, 132.91, 131.47, 130.06, 129.15, 127.97, 127.11, 125.09, 122.99, 119.64, 106.73, 94.00, 35.27, 31.34, 18.69, 11.65. HR MS (MALDI-TOF, positive): *m*/*z* Calcd for $C_{32}H_{41}OSi$: 468.2921 $[M+H]^+$, found: 468.2938

7-(5-*tert*-butyl-2-ethynylphenyl)-2-naphthaldehyde (**S4**)

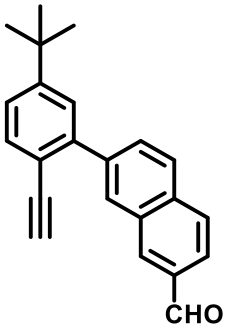

To a solution of 7-(5-*tert*-butyl-2-triisopropylsilylethynylphenyl)-2-naphthaldehyde (**S3**) (14.0 g, 29.8 mmol) in anhydrous THF (150 mL) was added TBAF (29.8 mmol, 29.8 mL, 1.00 M in THF) dropwise, and the mixture was stirred at room temperature for 2 h. After completion of the reaction was confirmed by TLC analysis, MeOH (30 mL) was added followed by stirring for another 30 min. Then the mixture was diluted with EA (200 mL), washed with water (100 mL) and brine (100 mL), dried over Na_2SO_4, and evaporated. The residue was purified by silica gel column chromatography (*n*-hexane/DCM = 2 : 1 to *n*-hexane/ DCM = 1 : 2) to yield the title compound (8.25 g, 88%) as white solid. TLC R_f = 0.3 (*n*-hexane/DCM = 2 : 1); ^{1}H NMR (250 MHz, $CDCl_3$) δ 10.19 (s, 1H), 8.41 (s, 1H), 8.20 (s, 1H), 8.03 – 7.87 (m, 4H), 7.62 (d, J = 8.1 Hz, 1H), 7.49 (s, 1H), 7.41 (d, J = 7.5 Hz, 1H), 2.99 (s, 1H), 1.38 (s, 9H); ^{13}C NMR (63 MHz, $CDCl_3$) δ 192.40, 152.78, 143.31, 139.88, 135.79, 134.95, 134.55, 133.93, 132.66, 131.11, 129.77, 129.04, 127.73, 126.97, 124.89, 123.15, 117.96, 83.18, 80.01, 35.12, 31.35. HR MS (MALDI-TOF, positive): *m*/*z* Calcd for $C_{23}H_{20}O$: 312.1514 $[M]^+$, found: 312.1523.

7,7'-{buta-1,3-diyne-1,4-diylbis(5-*tert*-butyl-2,1-phenylene)}bis(2-naphthaldehyde) (**S5**)

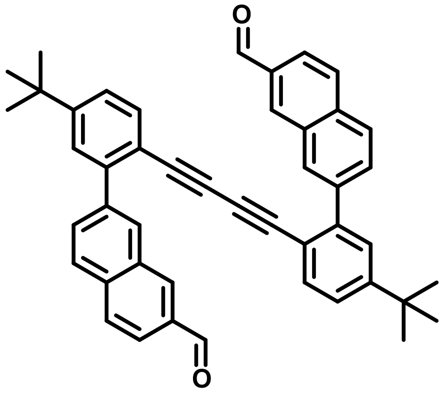

To a solution of 7-(5-*tert*-butyl-2-ethynylphenyl)-2-naphthaldehyde (**S4**) (6.00 g, 19.2 mmol) in dry *N*,*N*-dimethylformamide (DMF) (50 mL) was added CuCl (1.8 g, 18.2 mmol). The mixture was heated at 80 °C under air for 12 h. The mixture was then cooled down to room temperature, diluted with EA (300 mL), and washed with 1 N HCl (150 mL). The aqueous phase was extracted with EA (60 mL) for 3 times. The combined organic layers were washed with saturated solution of Na_2CO_3 (100 mL), brine (100 mL), dried over Na_2SO_4, and concentrated in vacuo. The residue was purified by silica gel column chromatography (*n*-hexane/DCM = 2 : 1 to *n*-hexane/DCM = 1 : 2) to yield the title compound (4.4 g, 74%) as white solid. TLC R_f = 0.3 (*n*-hexane/DCM = 2 : 1); ^{1}H NMR (250 MHz, CD_2Cl_2) δ 10.12 (s, 2H), 8.36 (s, 2H), 8.15 (s, 2H), 7.94 (s, 4H), 7.86 (s, 4H), 7.61 – 7.49 (m, 4H), 7.40 (d, J = 8.1 Hz, 2H), 1.36 (s, 18H); ^{13}C NMR (63 MHz, CD_2Cl_2) δ 192.39, 153.81, 144.14, 139.82, 135.98, 134.98, 134.95, 134.49, 132.99, 130.98, 129.85, 129.20, 127.97, 127.46, 125.36, 123.34, 117.63, 81.84, 76.60, 35.39, 31.25. HR MS (MALDI-TOF, positive): m/z Calcd for $C_{46}H_{38}O_2$: 622.2872 $[M]^+$, found: 622.2988.

9,9'-di-*tert*-butyl-6,6'-diiodo-[5,5'-bichrysene]-3,3'-dicarbaldehyde (**S6**)

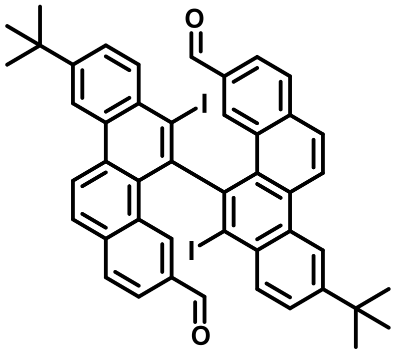

To a solution of compound **S5** (2.2 g, 3.53 mmol) dissolved in anhydrous DCM (250 mL) was added ICl (7.7 mL, 1.0 M in DCM) at –78 °C. After stirring at room temperature for 2 h, excess ICl was quenched by adding a saturated aqueous solution of $Na_2S_2O_3$ (50 mL). The

organic phase was separated, washed with brine (50 mL), dried over Na_2SO_4, and evaporated. The residual solid was recrystallized from DCM and MeOH to give the title compound (2.7 g, 87%) as white solid. ^1H NMR (250 MHz, CD_2Cl_2) δ 9.23 (d, J = 9.2 Hz, 2H), 8.96 (s, 2H), 8.73 (s, 2H), 8.60 (s, 2H), 8.37 (d, J = 8.8 Hz, 2H), 8.27 (d, J = 9.1 Hz, 2H), 8.03 (d, J = 8.3 Hz, 2H), 7.87 (d, J = 8.9 Hz, 2H), 7.79 (d, J = 8.2 Hz, 2H), 1.62 (s, 18H); ^{13}C NMR (63 MHz, CD_2Cl_2) δ 191.96, 152.23, 149.36, 137.33, 134.94, 134.44, 134.05, 132.72, 132.07, 130.68, 130.44, 130.31, 129.37, 128.13, 125.70, 123.45, 119.75, 113.98, 35.79, 31.56. HR MS (MALDI-TOF, positive): *m/z* Calcd for $C_{46}H_{36}I_2O_2$: 874.0805 $[M]^+$, found: 874.0745.

4,12-di-*tert*-butyldibenzo[*hi*,*st*]ovalene (DBOV **11**)

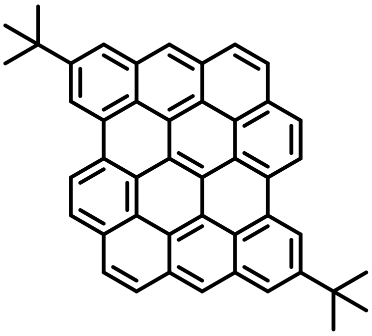

To a 3-L cylindrical quartz reactor containing 9,9'-di-*tert*-butyl-6,6'-diiodo-[5,5'-bichrysene]-3,3'-dicarbaldehyde (**S6**) (17.5 mg, 0.020 mmol) was added toluene (20 mL). Then the solution was degassed by bubbling with Ar for 20 min, and stirred at room temperature in a photoreactor for 2 h under irradiation by sixteen UV lamps (300 nm, 14 W) with stirring. After cooling down to a room temperature, the solvent was evaporated and the residue was filtered and wash with THF (20 mL) for several times, to give the title compound DBOV **11** as dark blue solid (10.2 mg, 87% yield). HR MS (MALDI-TOF, positive): *m/z* Calcd for $C_{46}H_{32}$: 584.2504 $[M]^+$, found: 584.2501. ^1H NMR and ^{13}C NMR spectra could not be recorded due to limited solubility and strong aggregation tendency. However, UV-vis and PL spectra as reported in Figure 2 in the main text agreed very well with those of previously reported DBOV derivatives,[11] evidencing the formation of the DBOV structure. UV-vis absorption spectra is also consistent with theoretically calculated result shown in Figure S23.

Imaging and Sensor Details

Coverslip cleaning

The coverslips were sonicated in 1% Micro 90 alkaline cleaning solution for 15 min. Then the coverslips were rinsed three times with Milli-Q water and finally dried with nitrogen flow. Afterward, those coverslips were cleaned by oxygen-plasma cleaner (250 W, 10 min).[12]

Coating of polystyrene film

The purification of polystyrene was carried out using an anti-solvent crystallization method.[13] The polystyrene solid was dissolved in anhydrous THF to obtain a clear solution. Then an equal volume of MeOH was added to the solution, and the resulting mixture was let stand until it became a clear solution again and all the crystals were precipitated. The solvents were subsequently removed and the precipitates were washed with MeOH, and then dried in a vacuum desiccator prior to the use.

100 μL of the solution of polystyrene purified above (4 mg/mL in toluene) was spin-coated on the cleaned coverslip. The coverslip was firstly spun at 2,000 rpm for 20 s and then at 4,000 rpm for 40 s. The sample was dried on a hot plate by heating at 90 °C for 10 min.

Preparation of samples for photostability measurements

10 μL of the nanographene solution (1 μM in toluene)/Alexa 647 solution (1 μM in H_2O) was spin-coated on the cleaned coverslip. The coverslip was firstly spun at 2,000 rpm for 20 s and then at 4,000 rpm for 40 s. The samples were dried in air for 1 day before the measurements.

Preparation of nanographene sample for 3D confocal imaging

10 μL of the nanographene solution (1 μM in toluene) was added on a cleaned gridded glass coverslip (ibidi Grid-50) and the measurements were performed after toluene has completely evaporated.

Preparation of nanographene samples for pH sensitive single-molecule blinking property measurements

10 μL of a solution of nanographene (10^{-12} M in toluene/ethanol=1/99) was spin-coated on the polystyrene coated coverslip. The coverslip was firstly spun at 2,000 rpm for 20 s and then

at 4,000 rpm for 40 s. The sample was dried on a hot plate in N_2 glove box by heating at 80 °C for 10 min.

Preparation of nanographene sample for super-resolution imaging

10 μL of the nanographene solution (0.1 nM in toluene) was added on a cleaned gridded glass coverslip (ibidi Grid-50) and the measurements were performed after toluene has completely evaporated.

Photostability characterization

Photostability measurements were performed with a home-build single molecule localization microscope.[14] The set up used a confocal microscope (Leica DM RBE) that served as the microscope body and was connected with a CMOS camera (Photometrics BSI). It has been equipped with a 532 nm laser with 2 emission filters (>580 nm/590–670 nm). Laser beams were focused on the back focal plane of the 100×/NA 1.40 objective (Olympus). To obtain laser intensity, the field of view was first tested with a commercial fluorescence slide. Next, the $1/e^2$ width of a line plot through the illuminated area was used to approximate the diameter of the circular illuminated area. This yielded an illumination area of about 1.27×10^{-4} cm^2. Laser intensity (25.59 W/cm^2) was obtained by dividing the laser power (3.25 mW was used, tested under the objective) by the area.

3D confocal imaging

3D confocal imaging was performed using the TCS SP5 confocal microscope (Leica). 561 nm laser was selected for excitation. The fluorescence emission was continuously adjusted between 400–800 nm and detected by 4 PMTs. 63.0 × 1.40 oil objective was used.

pH-sensitive single-molecule blinking characterization and super-resolution imaging

Both pH-sensitive single-molecule blinking property measurement and super-resolution imaging were performed using the SR GSD microscope (Leica). 532 nm (500 mW) laser was selected for fluorescence reactivation. For the 532 nm laser, the excitation filter (527–537 nm/400–410 nm), the dichroic beamsplitter (527–537 nm/400–410 nm), and the emission filter (550–650 nm/449–451 nm) were used. The objective lens HCX PL APO 160× 1.43 NA Oil CORR-TIRF was selected for single-molecule measurements and super-resolution imaging. The microscope was equipped with an EMCCD camera (iXonDU-897, Andor). The camera settings were 10 MHz at 14 bit and a pre-amplification of 5.1. For super-resolution imaging,

the camera exposure time was set to 30 ms and an EM gain of 100 was used. Please note here that the double bandwidth of the filters/beamsplitter were chosen for 405 nm back pumping and in our experiments mentioned in this work, such back pumping was not used.

Fluorescence sensing of proton

A solution of N-DBOV **10** or DBOV **11** (5.0×10^{-5} mol L^{-1}) was prepared in THF. The solutions of hydrochloric acid (HCl) were prepared in MeOH with a concentration of 3×10^{-3} M. The solution of N-DBOV **10** or DBOV **11** (2.7 mL) was placed in a quartz cell and the fluorescence spectrum was recorded. Then different amounts (50 μL, 100 μL, 150 μL, 200 μL, 250 μL and 300 μL) of the HCl solutions were added. Besides, a certain amount of extra THF solvent was added to the quartz cell to make the total solution volume constant (3 mL) so that the concentration of **10** or **11** stays the same in different measurements. Finally, fluorescence spectra with different intensity were recorded at room temperature exciting with the same wavelength of 580 nm.

Fluorescence intensity recovery of N-DBOV + H^+ with TEA

A THF solution of TEA was prepared (3.0×10^{-3} mol L^{-1}). A fluorescence quenched solution of N-DBOV **10** was placed in a quartz cell (2.7 mL) and TEA solution was gradually added to turn on the fluorescence. The fluorescence spectra and intensity were recorded at room temperature exciting at wavelength of 580 nm.

Fluorescence sensing of metal ions (Cu^{2+}, Fe^{2+} and Mg^{2+})

A Cu^{2+} stock solution (9.0×10^{-4} mol L^{-1}) was prepared by dissolving $Cu(ClO_4)_2{\cdot}6H_2O$ (33 mg) in THF (100 mL). A solution (2.7 mL) of N-DBOV **10** or DBOV **11** was placed in a quartz cell and fluorescence spectra were recorded immediately after the addition of a specified amount (0.2 eq, 0.4 eq, 0.6 eq, 0.8 eq, 1.0 eq, 1.2 eq, 1.4 eq, 1.6 eq and 1.8 eq for N-DBOV **10**; 0.2 eq, 0.4 eq, 0.6 eq, 0.8 eq, 1.0 eq, 1.2 eq and 1.4 eq for DBOV **11**) of the Cu^{2+} solution, upon excitation at 580 or 586 nm, respectively. A certain amount of extra THF solvent was added to the quartz cell to make the total solution volume constant (3 mL) so that the concentration of **10** or **11** stays the same in different measurements. Each fluorescence spectra of different detection limit was measured at least for three times to get the reliable value. The Fe^{2+} or Mg^{2+} stock solutions (9×10^{-5} mol/L) were prepared by dissolving $Fe(ClO_4)_2{\cdot}xH_2O$ or $Mg(ClO_4)_2$ in THF (100 mL), whereas other operations were the same that of the Cu^{2+} detection.

Supplementary Figures and Tables

NMR spectra

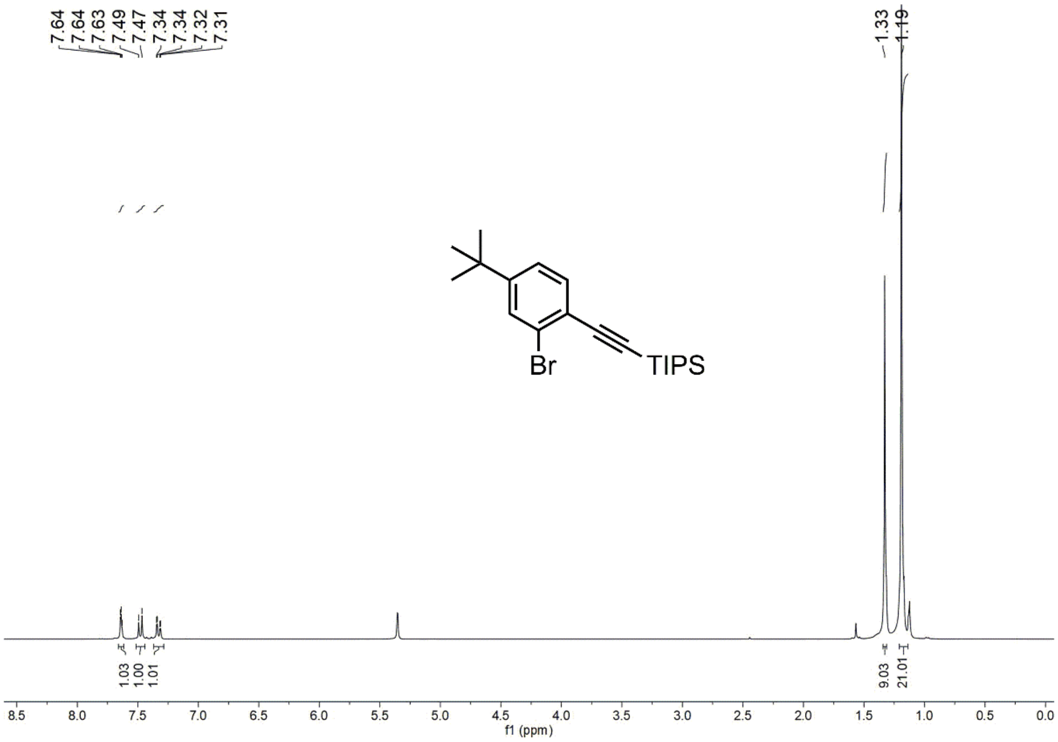

Figure S1. ^{1}H NMR spectrum of compound **2** measured in CD_2Cl_2 (300 MHz, 298 K).

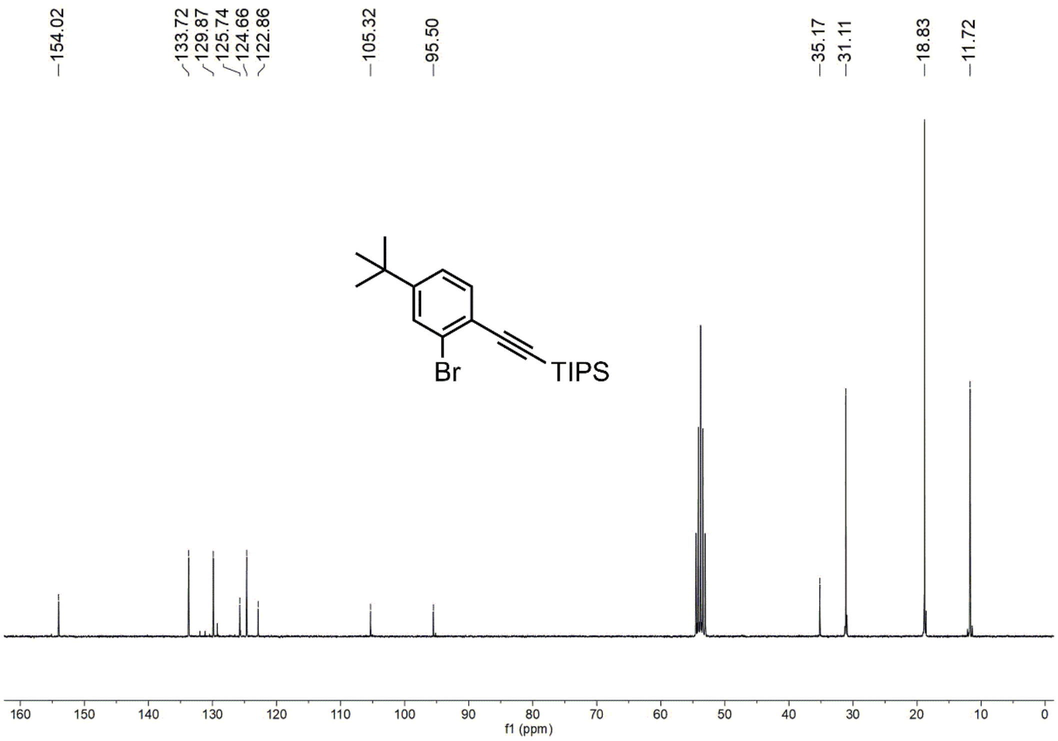

Figure S2. ^{13}C NMR spectrum of compound **2** measured in CD_2Cl_2 (75 MHz, 298 K).

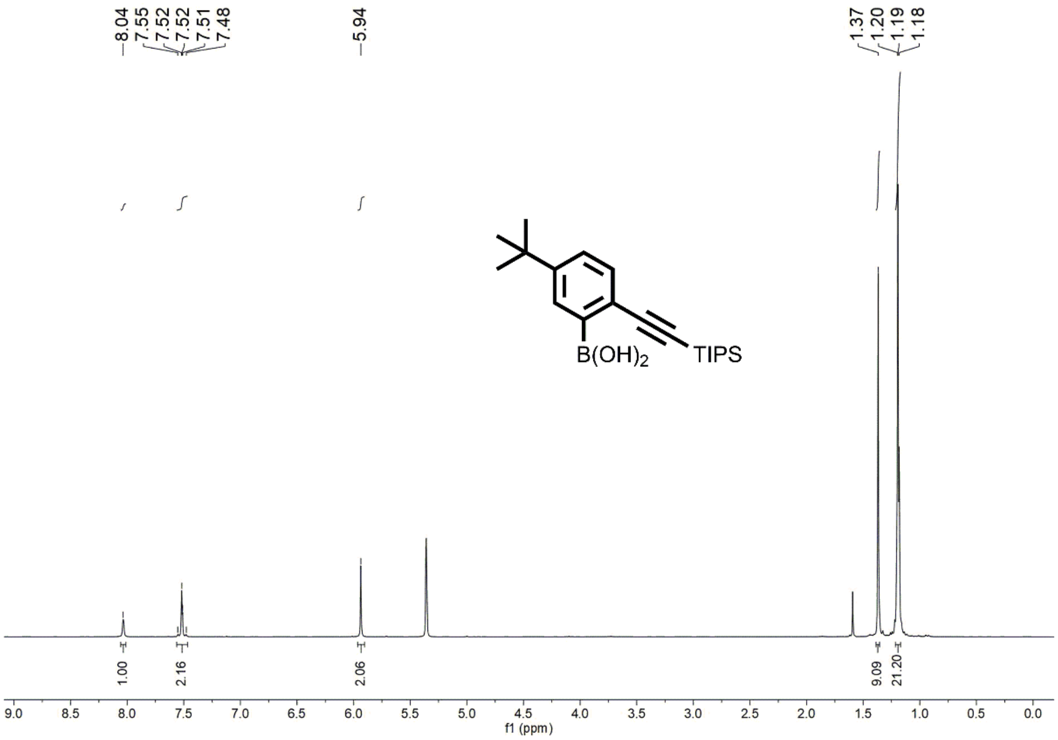

Figure S3. ^{1}H NMR spectrum of compound **3** measured in CD_2Cl_2 (250 MHz, 298 K).

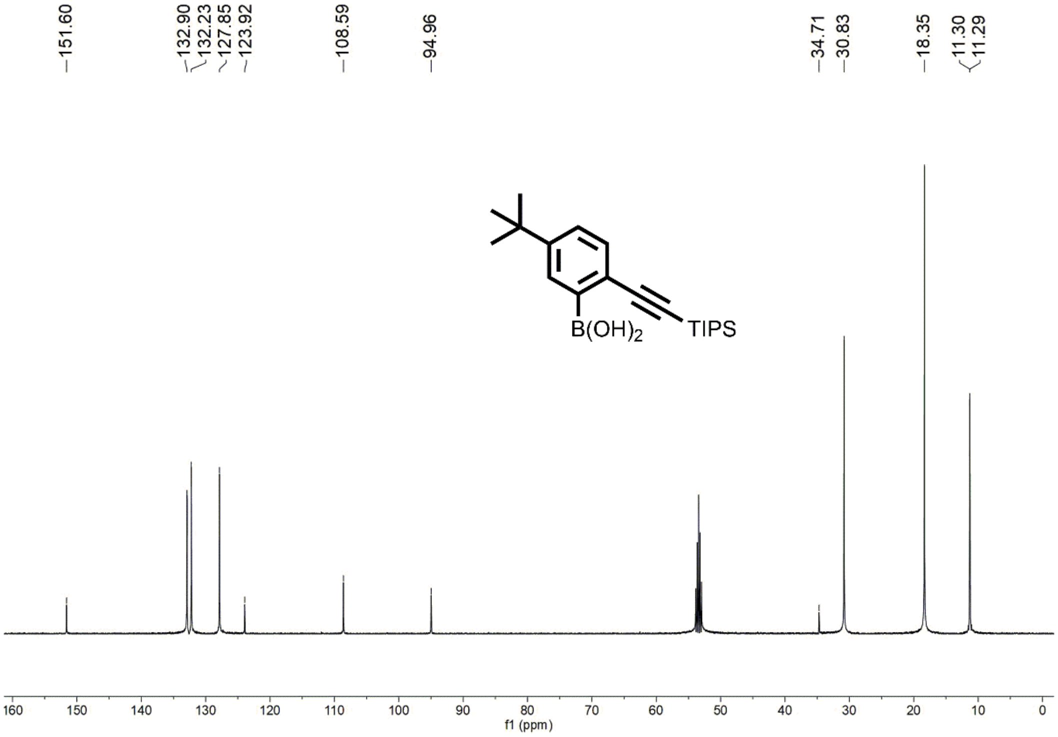

Figure S4. ^{13}C NMR spectrum of compound **3** measured in CD_2Cl_2 (125 MHz, 298 K).

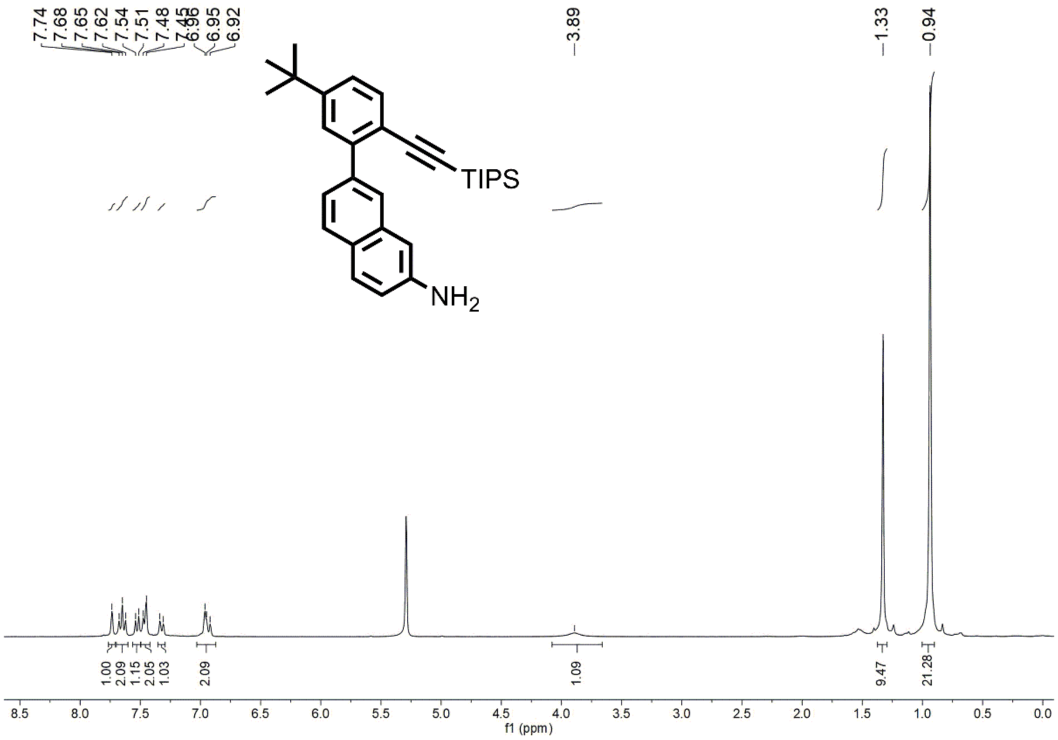

Figure S5. ^{1}H NMR spectrum of compound **5** measured in CD_2Cl_2 (300 MHz, 298 K).

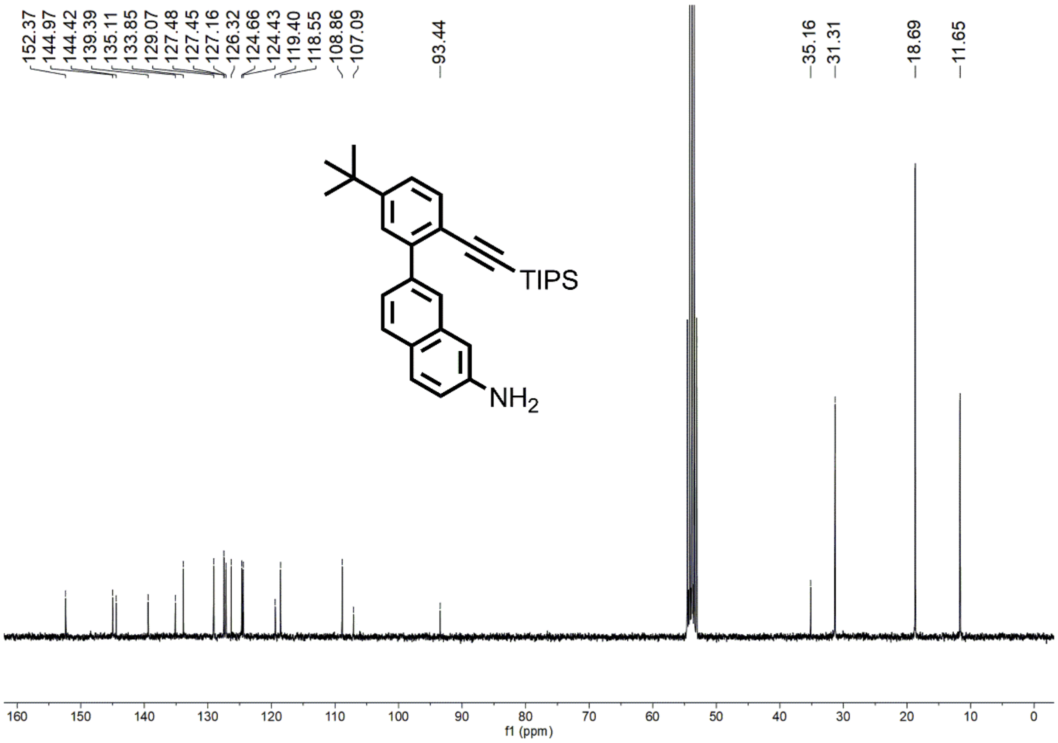

Figure S6. ^{13}C NMR spectrum of compound **5** measured in CD_2Cl_2 (75 MHz, 298 K).

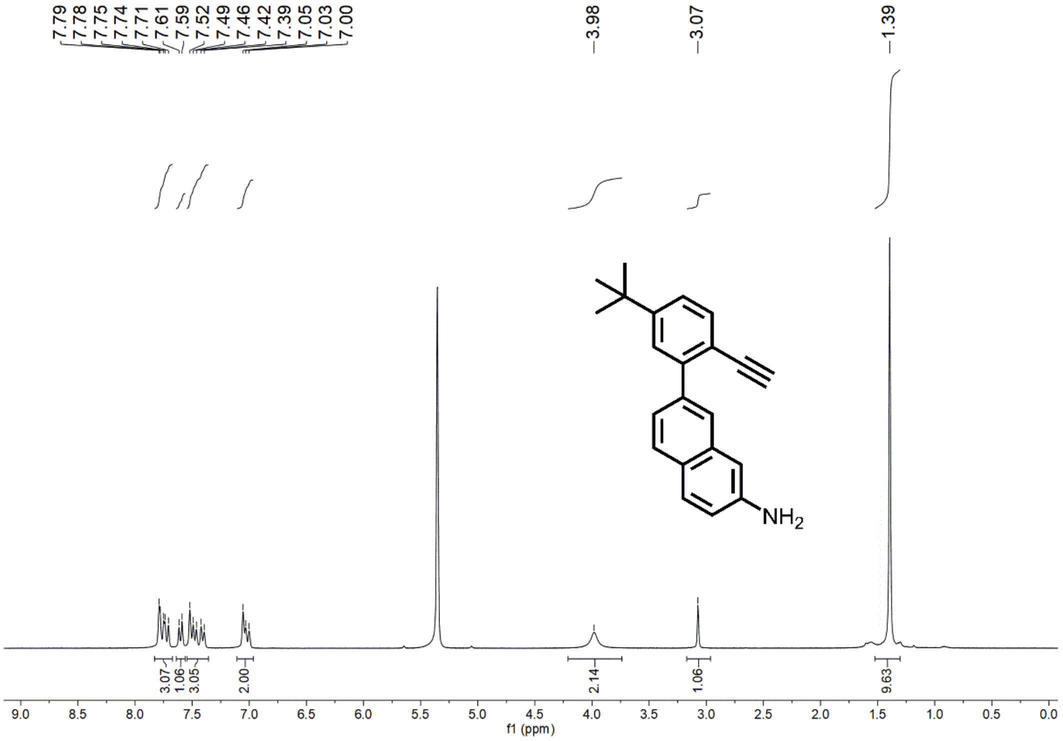

Figure S7. ^{1}H NMR spectrum of compound **6** measured in CD_2Cl_2 (300 MHz, 298 K).

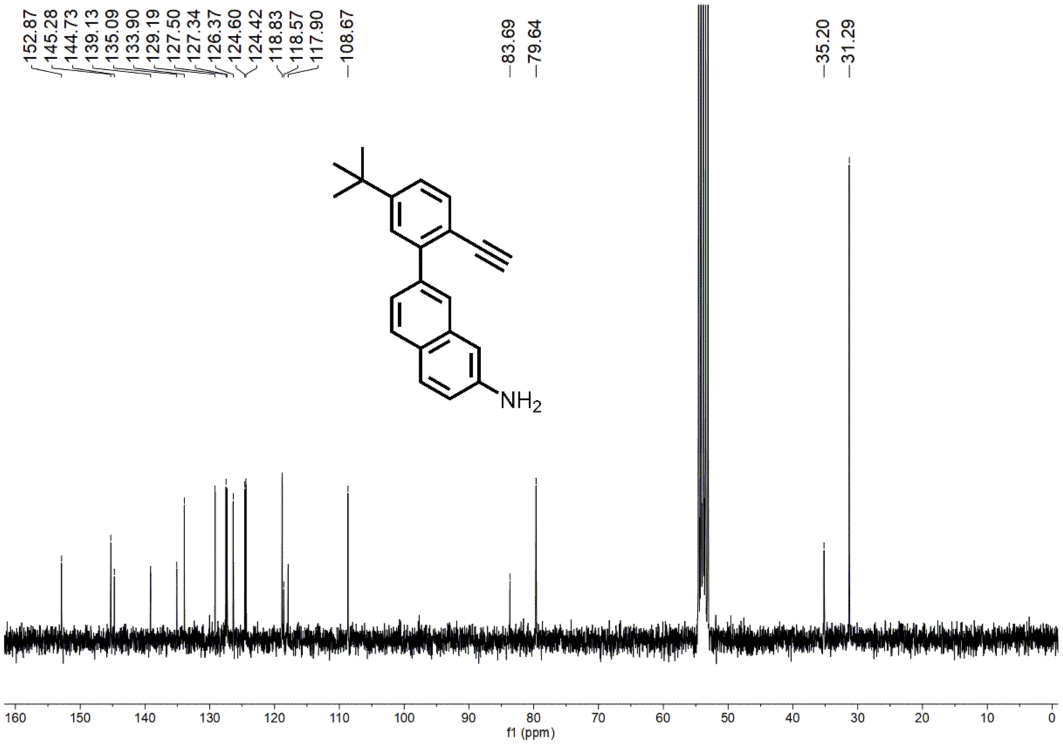

Figure S8. ^{13}C NMR spectrum of compound **6** measured in CD_2Cl_2 (75 MHz, 298 K).

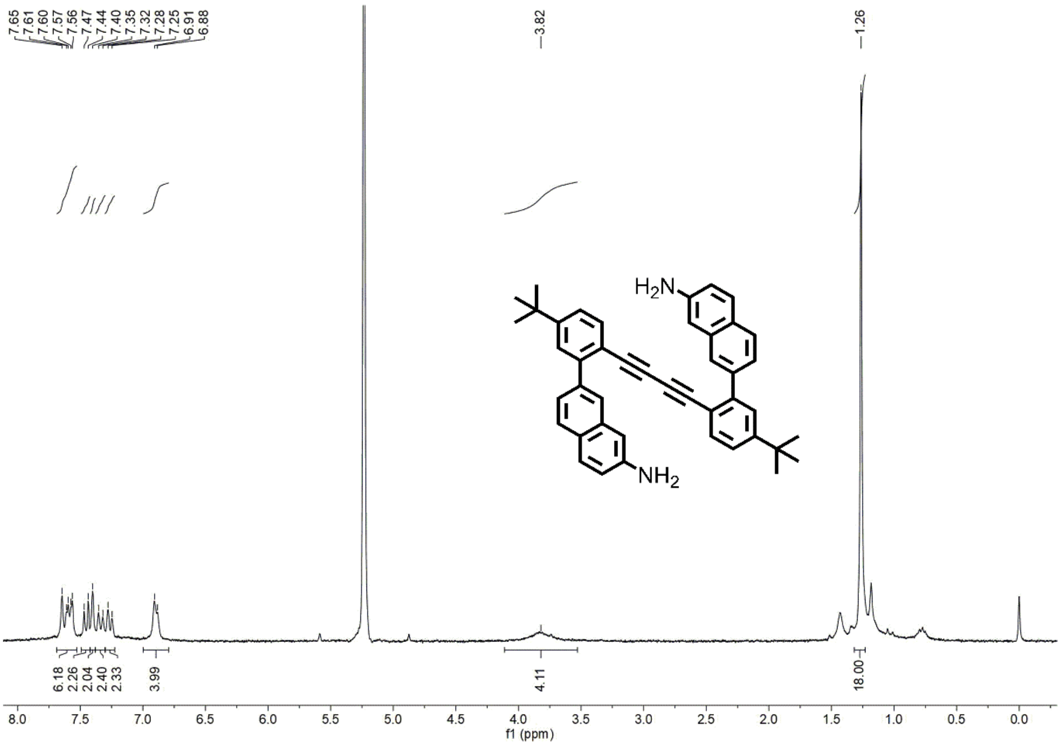

Figure S9. ^{1}H NMR spectrum of compound **7** measured in CD_2Cl_2 (300 MHz, 298 K).

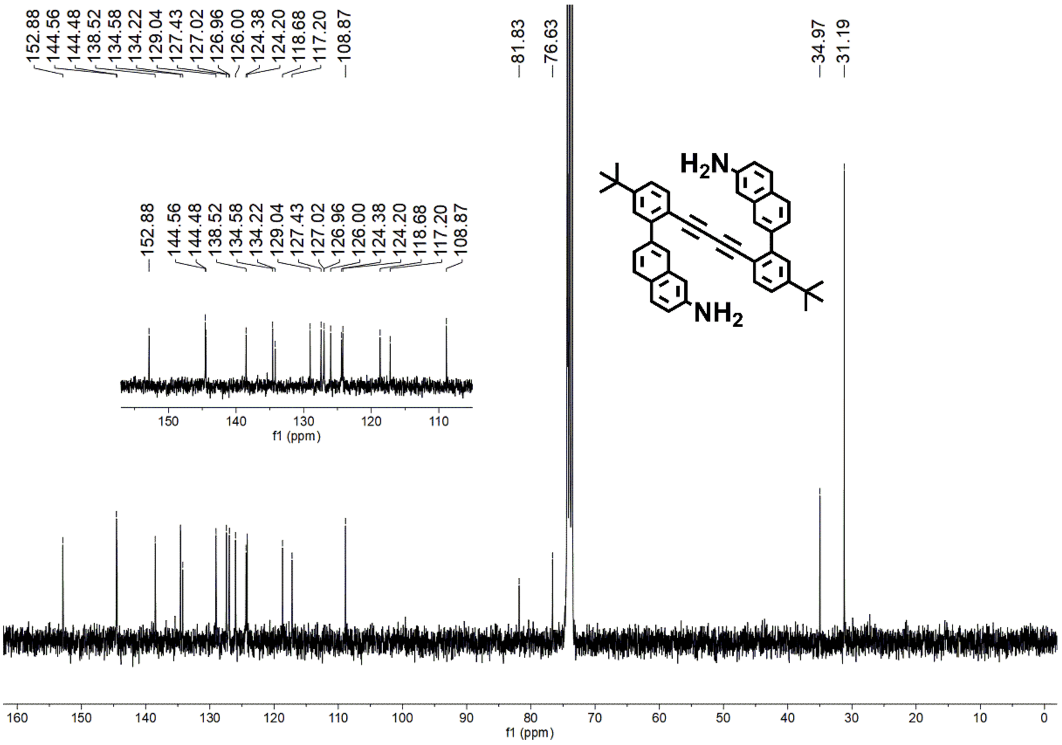

Figure S10. ^{13}C NMR spectrum of compound **7** measured in $C_2D_2Cl_4$ (75 MHz, 298 K).

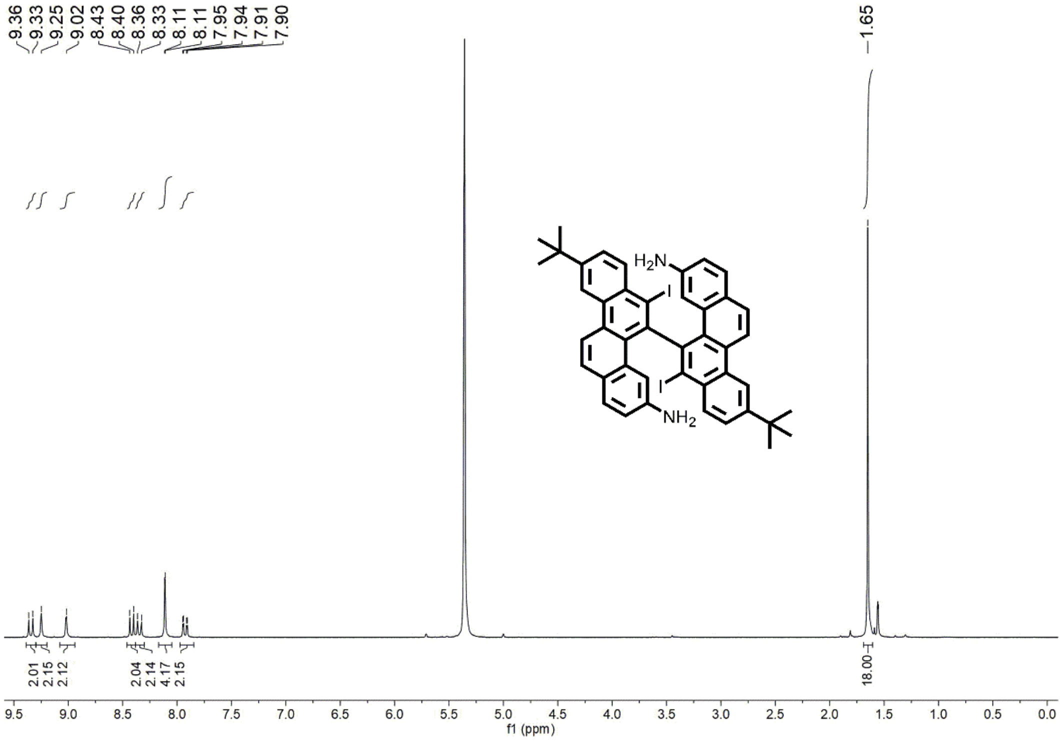

Figure S11. ^{1}H NMR spectrum of compound **8** measured in CD_2Cl_2 (250 MHz, 298 K).

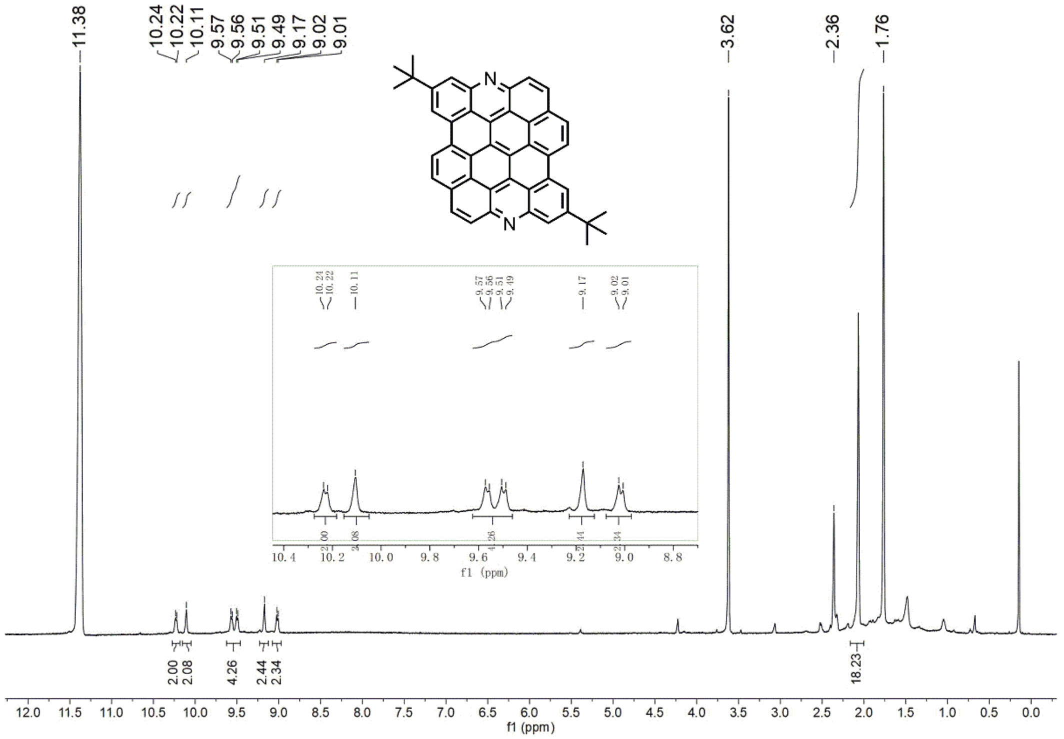

Figure S12. ^{1}H NMR spectrum of N-DBOV **10** measured in THF-d_8/ TFA-d_1 (250 MHz, 298 K).

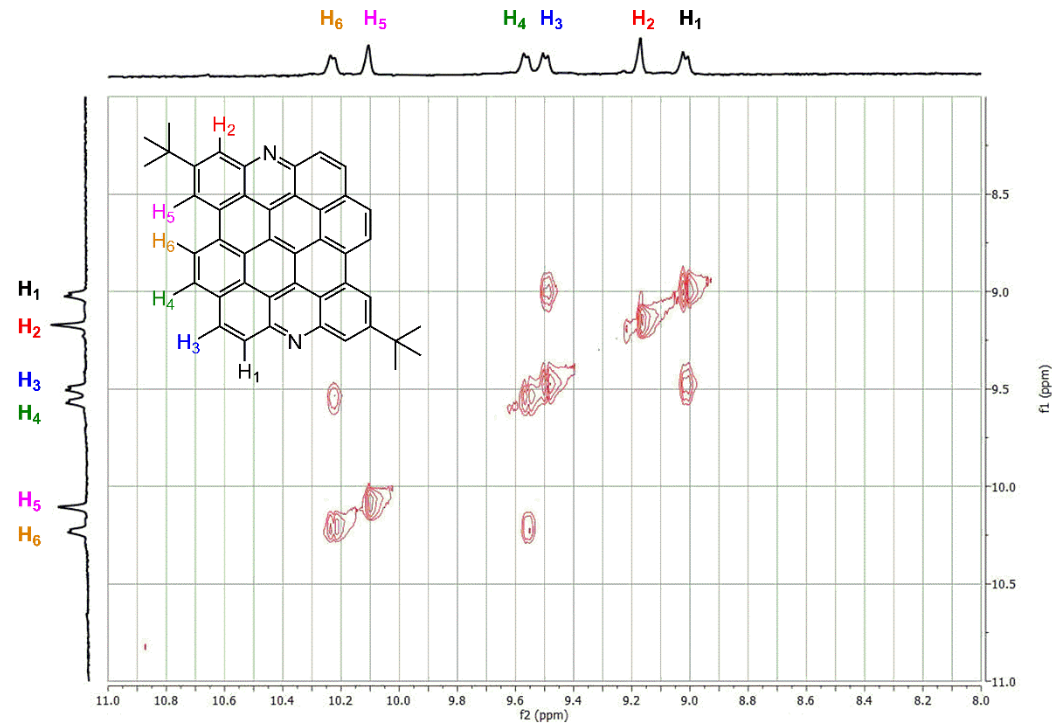

Figure S13. Aromatic region of ^{1}H,^{1}H-COSY spectrum of N-DBOV **10** (500 MHz, THF-d_8/ TFA-d_1) at 298K.

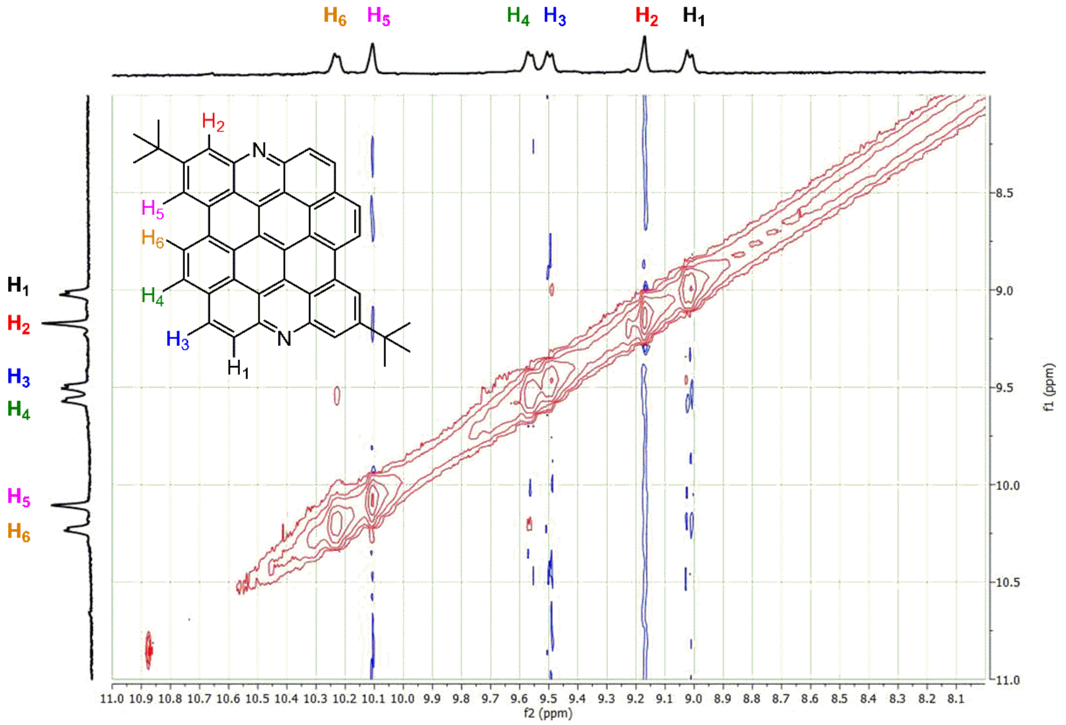

Figure S14. Aromatic region of ^{1}H,^{1}H-NOESY spectrum of N-DBOV **10** (500 MHz, THF-d_8/TFA-d_1) at 298 K.

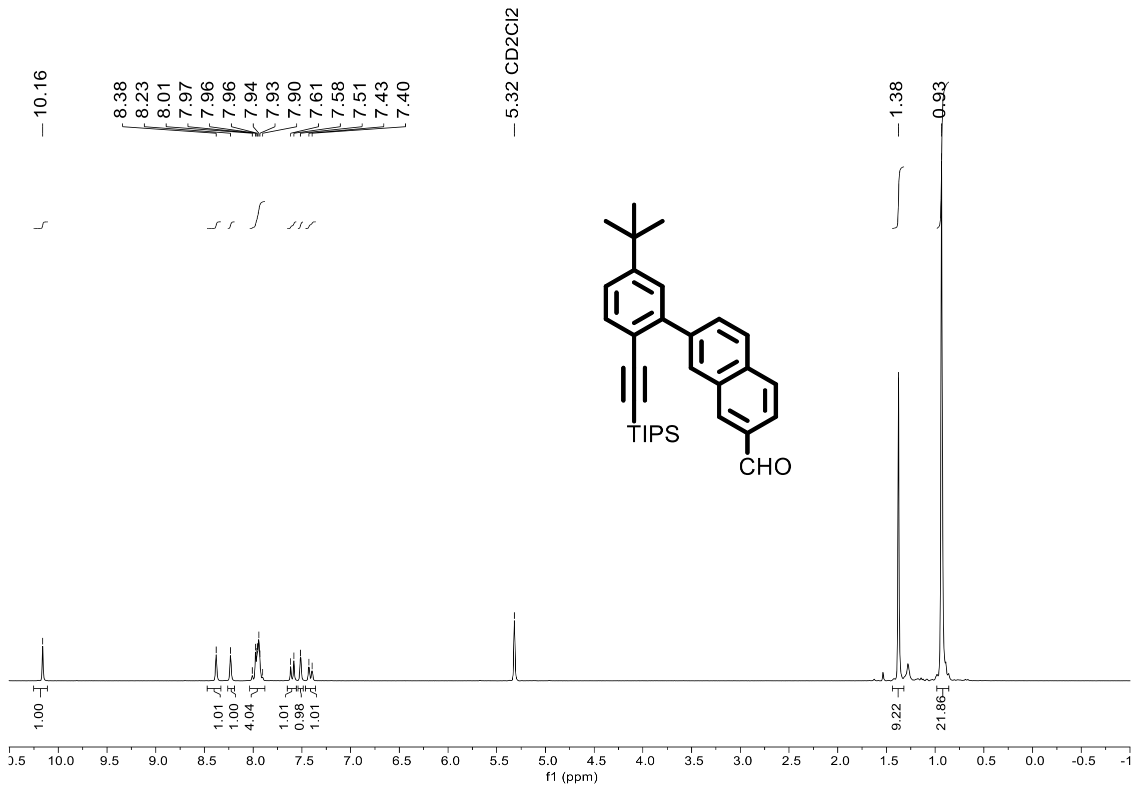

Figure S15. ^{1}H NMR spectrum of compound **S3** measured in CD_2Cl_2 (250 MHz, 298 K).

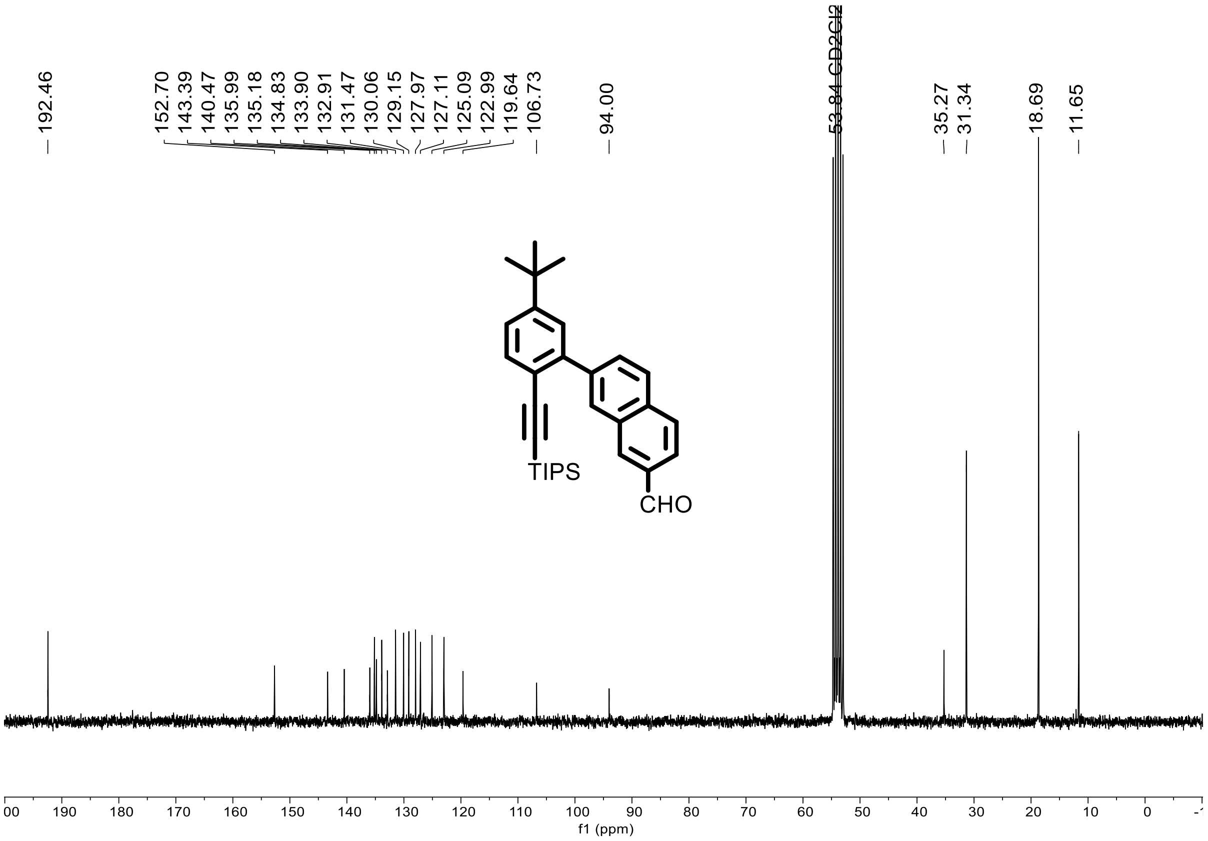

Figure S16. ^{13}C NMR spectrum of compound **S3** measured in CD_2Cl_2 (63 MHz, 298 K).

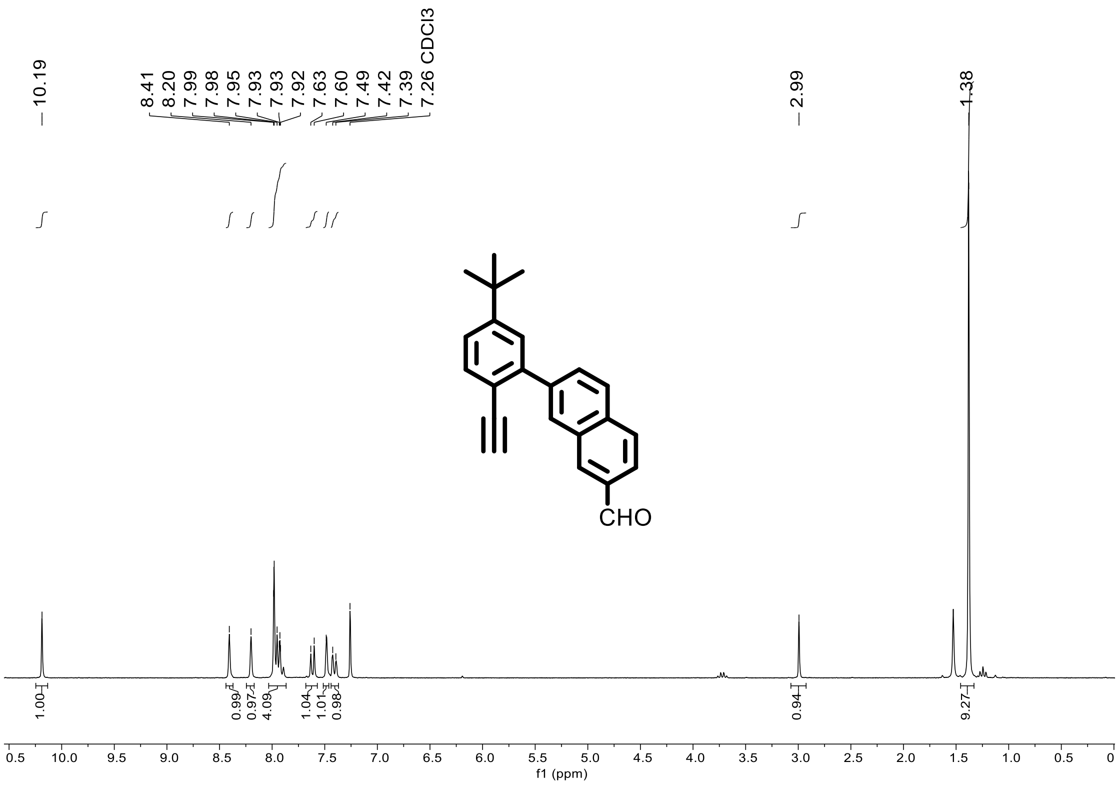

Figure S17. ^{1}H NMR spectrum of compound **S4** measured in $CDCl_3$ (250 MHz, 298 K).

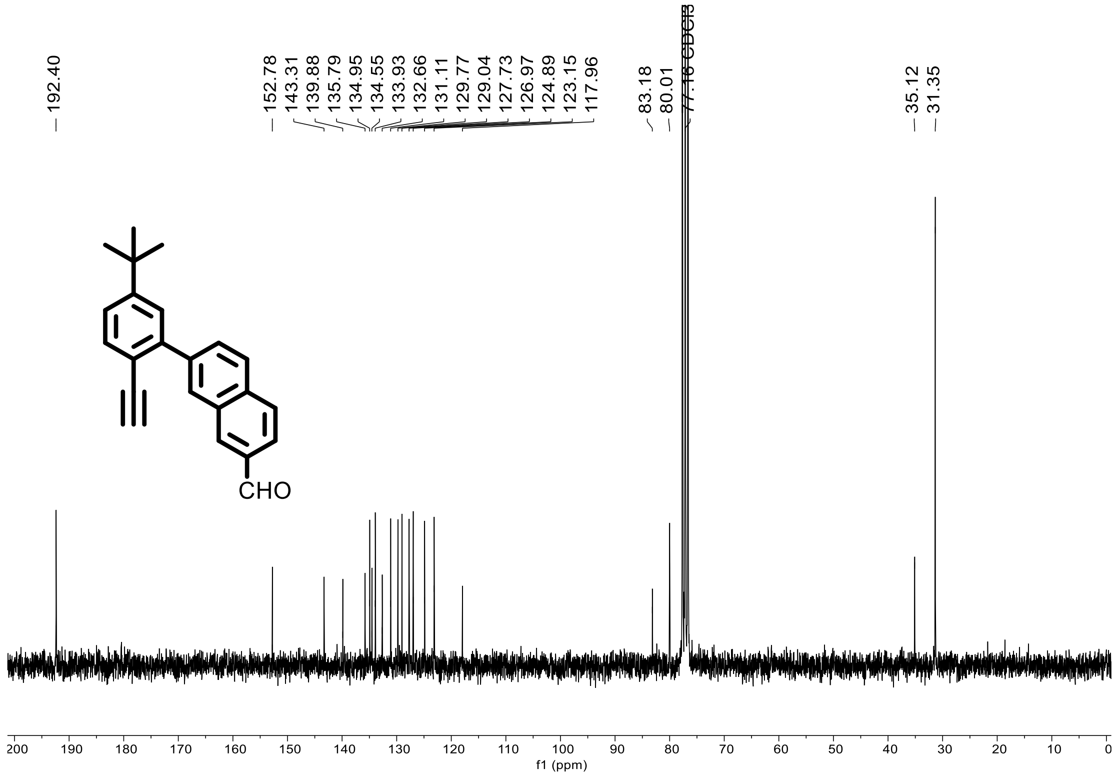

Figure S18. ^{13}C NMR spectrum of compound **S4** measured in $CDCl_3$ (63 MHz, 298 K).

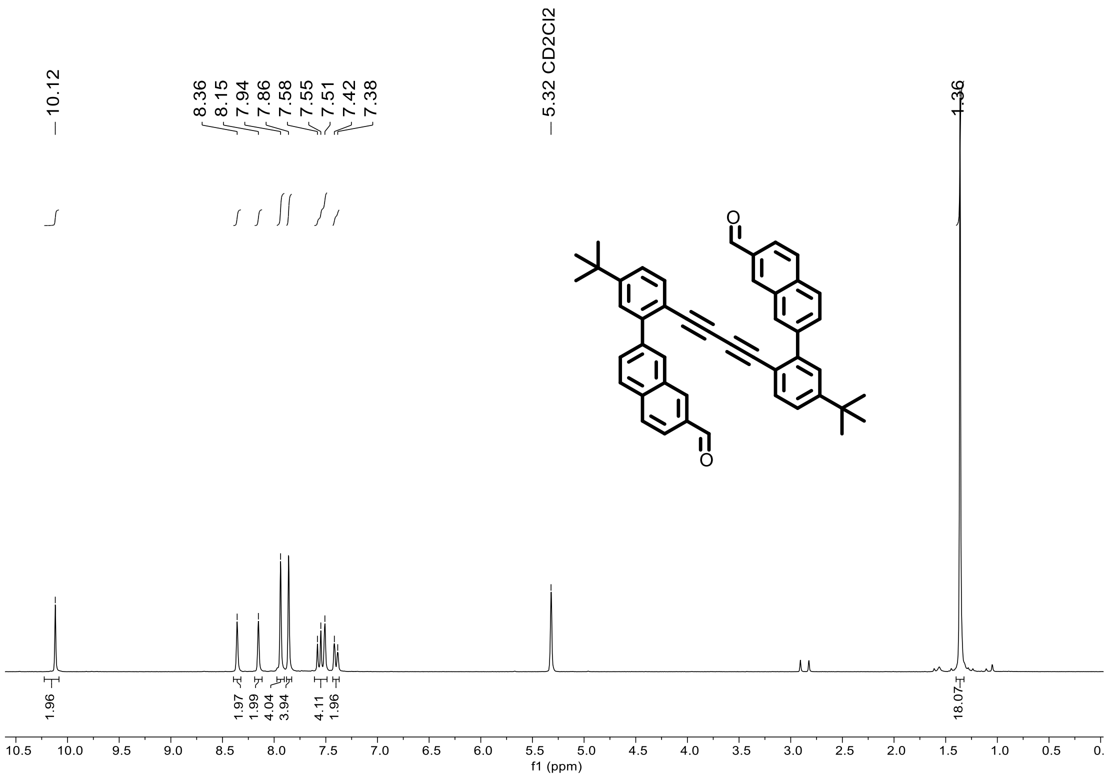

Figure S19. ^{1}H NMR spectrum of compound **S5** measured in CD_2Cl_2 (250 MHz, 298 K).

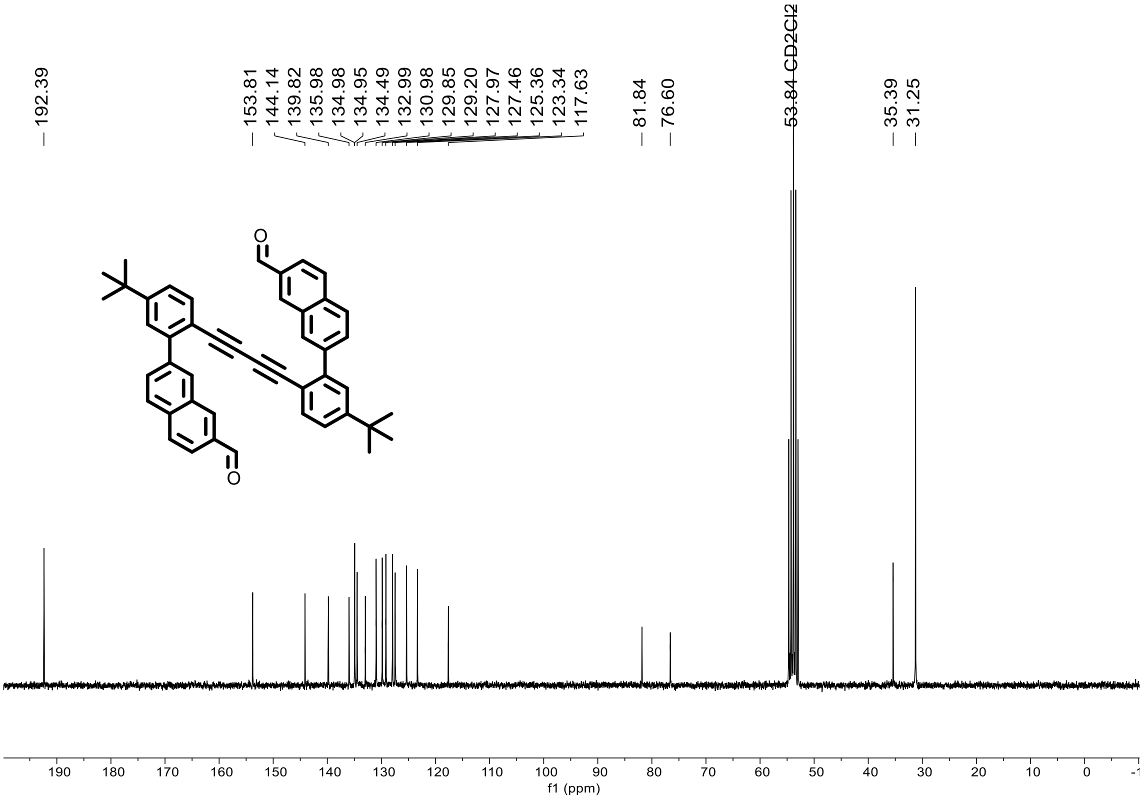

Figure S20. ^{13}C NMR spectrum of compound **S5** measured in CD_2Cl_2 (63 MHz, 298 K).

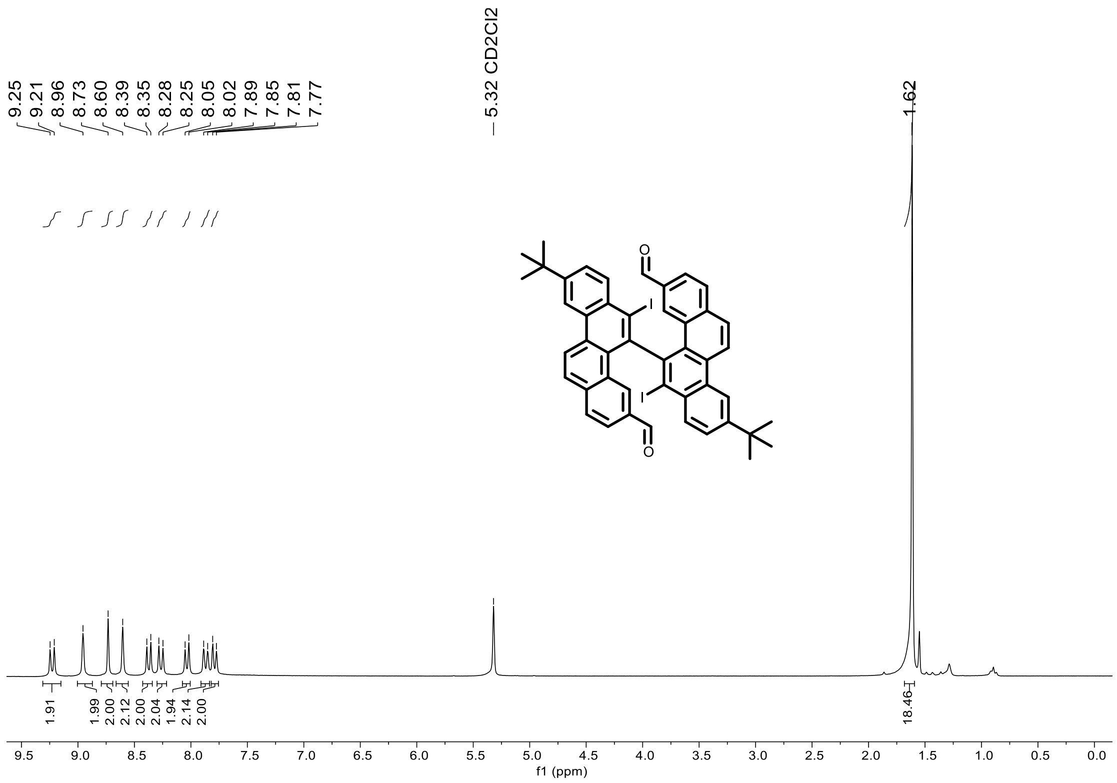

Figure S21. ^{1}H NMR spectrum of compound **S6** measured in CD_2Cl_2 (250 MHz, 298 K).

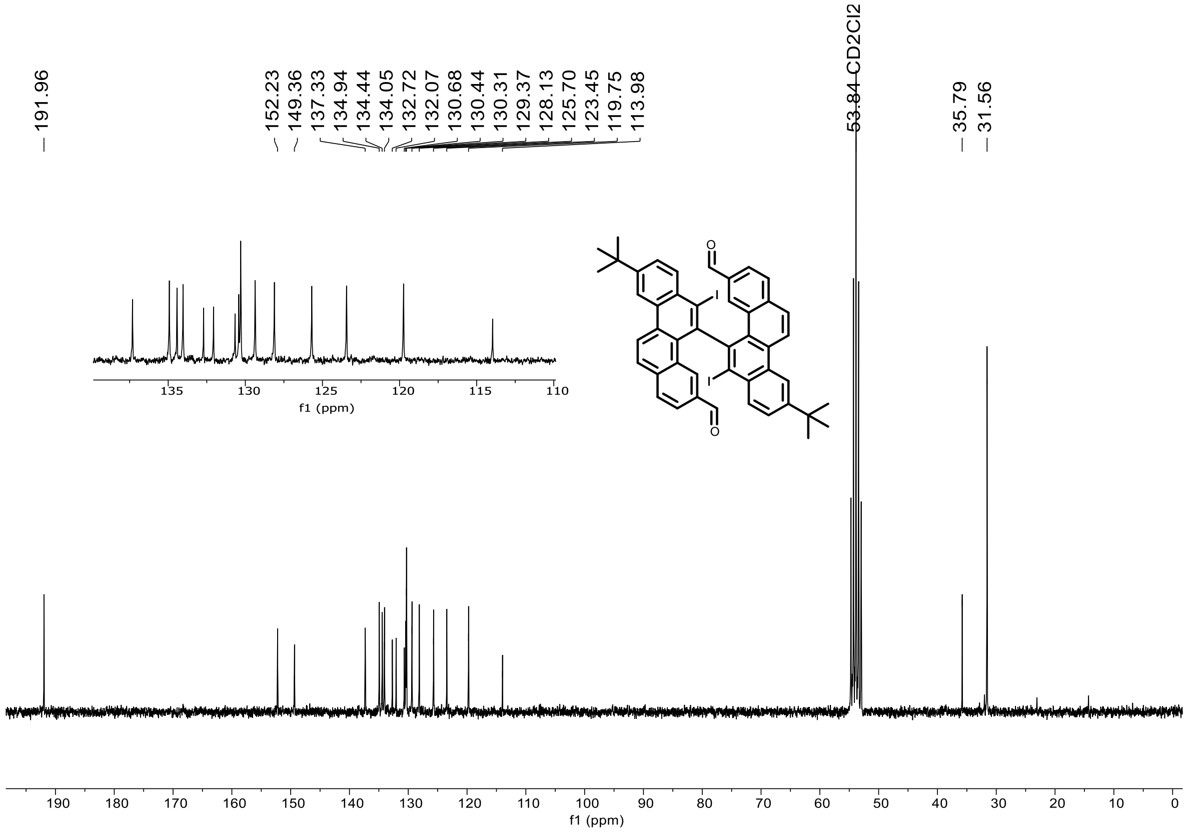

Figure S22. ^{13}C NMR spectrum of compound **S6** measured in CD_2Cl_2 (63 MHz, 298 K).

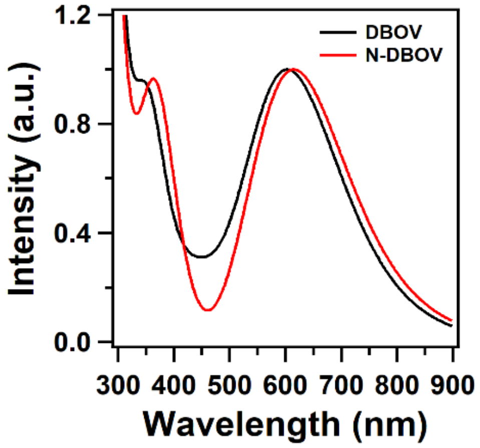

Figure S23. UV-vis spectra of N-DBOV **10** and DBOV **11** by TD-DFT method.

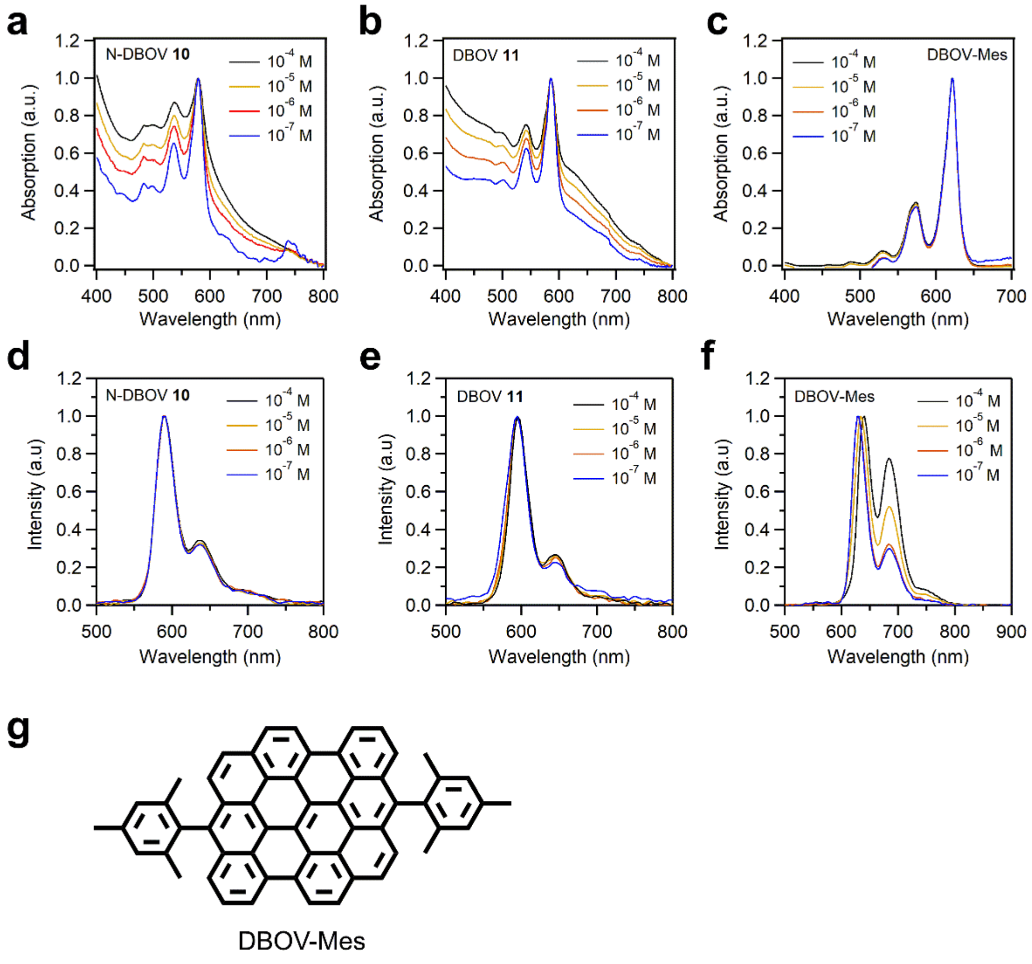

Figure S24. The concentration-dependent UV-vis and PL spectra of N-DBOV **10**, DBOV **11** and DBOV-Mes in THF solutions measured at room temperature. UV-vis absorption spectra of (a) N-DBOV **10**, (b) DBOV **11** and (c) DBOV-Mes; PL spectra of (d) N-DBOV **10**, (e) DBOV **11** and (f) DBOV-Mes; (g) chemical structure of DBOV-Mes.

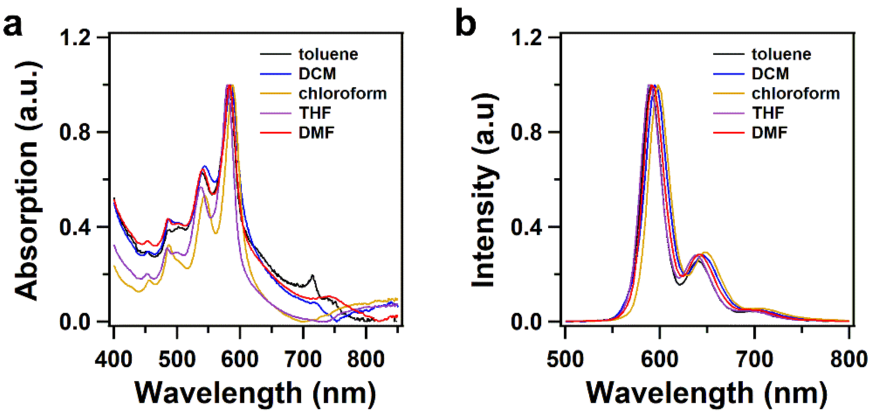

Figure S25. (a) Absorption and (b) emission spectra of N-DBOV **10** in different solvents. Concentration: 10 μM.

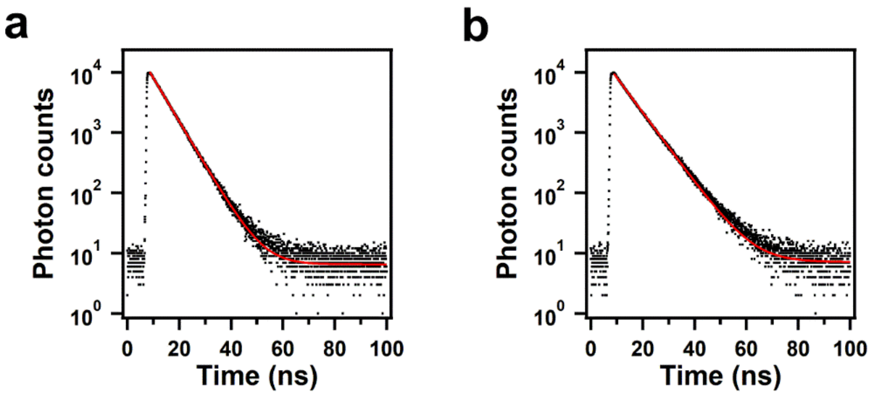

Figure S26. Fluorescence quantum lifetime decay curves for (a) N-DBOV **10** and (b) DBOV **11** in THF solutions and monoexponential fits. Concentration: 10 μM. The lifetime τ was determined to be 6.0 ns for N-DBOV **10** and 7.5 ns for DBOV **11**.

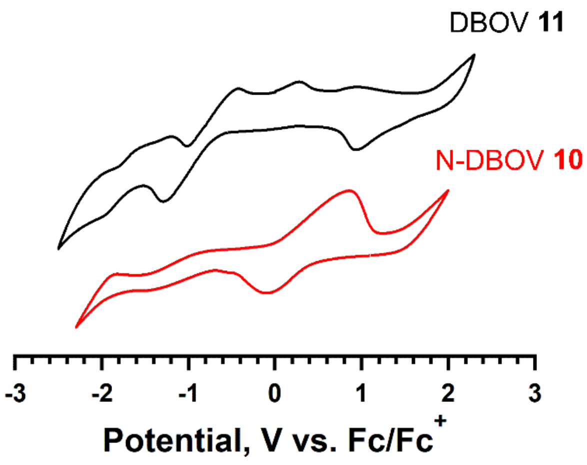

Figure S27. Cyclic voltammograms of N-DBOV **10** and DBOV **11** measured in 0.1 M tetra-*n*-butylammonium hexafluorophosphate in *o*-DCB.

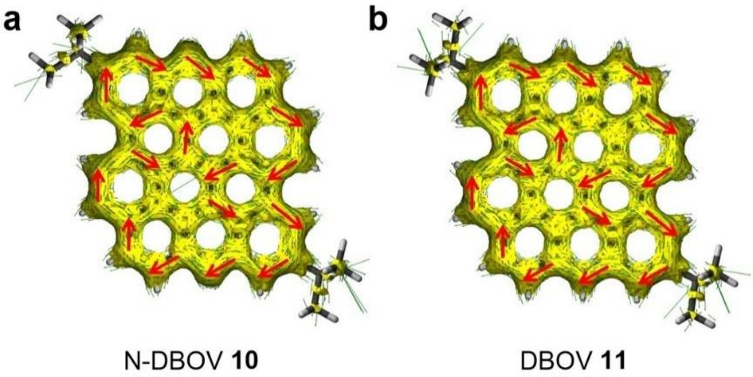

Figure S28. Calculated ACID (B3LYP/6-31G(d)) plots of N-DBOV **10** (left) and DBOV **11** (right) (isovalue = 0.05). Only contributions from π-electrons of the aromatic cores are considered. The magnetic field vector is perpendicular to the ring plane and points outward. Red arrows indicate directions of induced ring current.

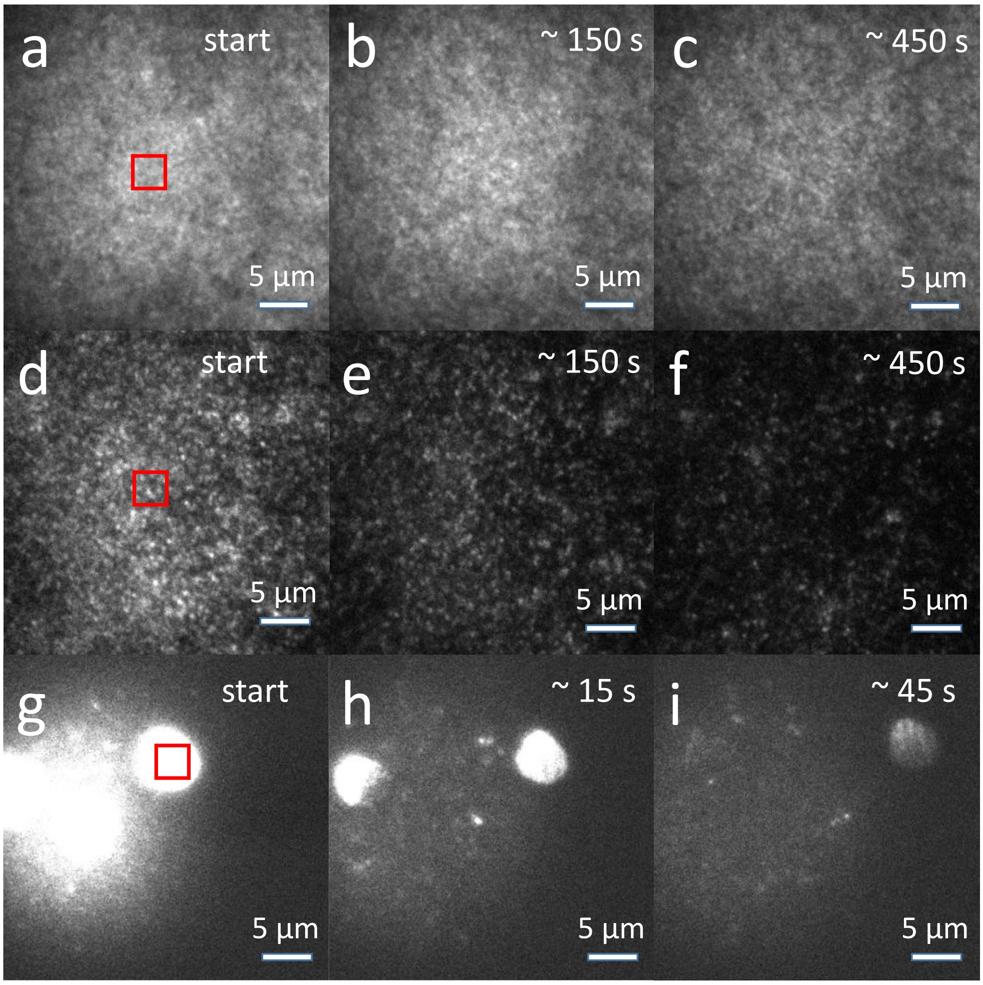

Figure S29, Photostability measurements for N-DBOV **10** (a-c), DBOV **11** (d-f) and Alexa 647 (g-i). The measurements of fluorescence retention in Fig. 4 are from the region of interest shown in (a, d, g) respectively.

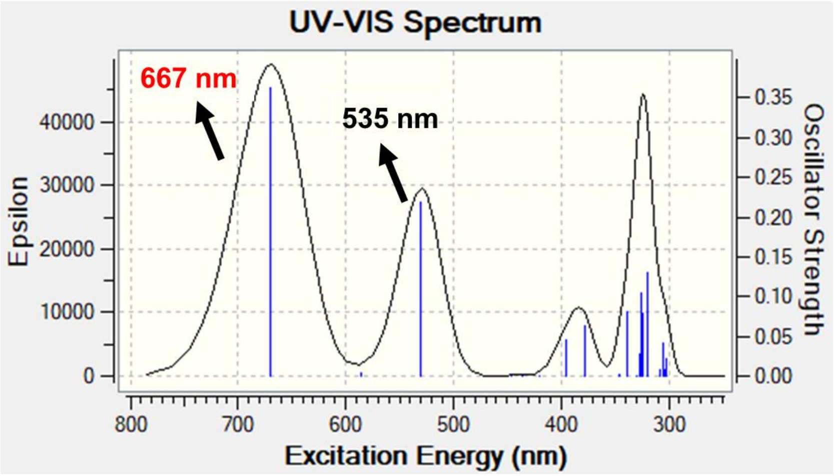

Figure S30. UV-vis absorption spectra of N-DBOV-$2H^+$ by TD-DFT method.

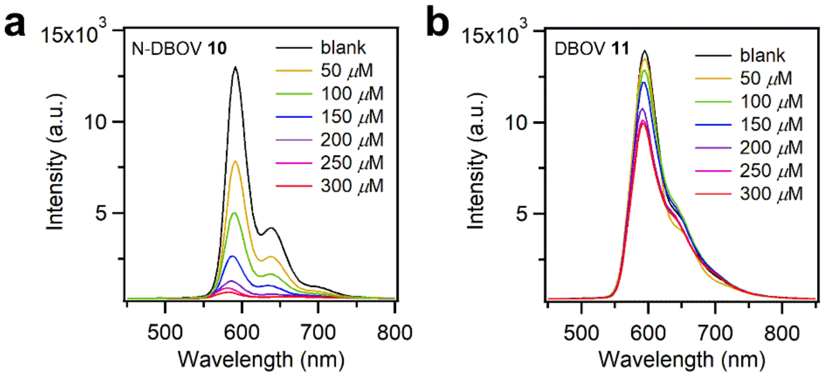

Figure S31. Changes in the fluorescence spectra of 5 × 10^{-5} M (a) N-DBOV **10** and (b) DBOV **11** in THF solution (3 mL) measured at room temperature upon successive addition of 3 × 10^{-3} M hydrochloric acid in MeOH (300 μL) and the legend shows the analytical concentration of HCl in the measured sample.

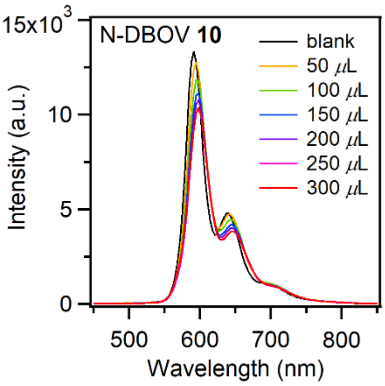

Figure 32. Changes in the fluorescence spectra of N-DBOV **10** in THF (5 × 10^{-5} M) at room temperature upon successive addition of pure MeOH.

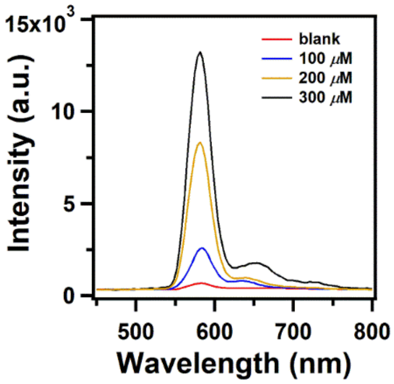

Figure S33. Fluorescence intensity recovery of N-DBOV **10** after the quenching upon protonation to N-DBOV-2H$^+$ (shown as blank with red line), via subsequent addition of a solution of triethylamine in THF (3×10^{-3} M).

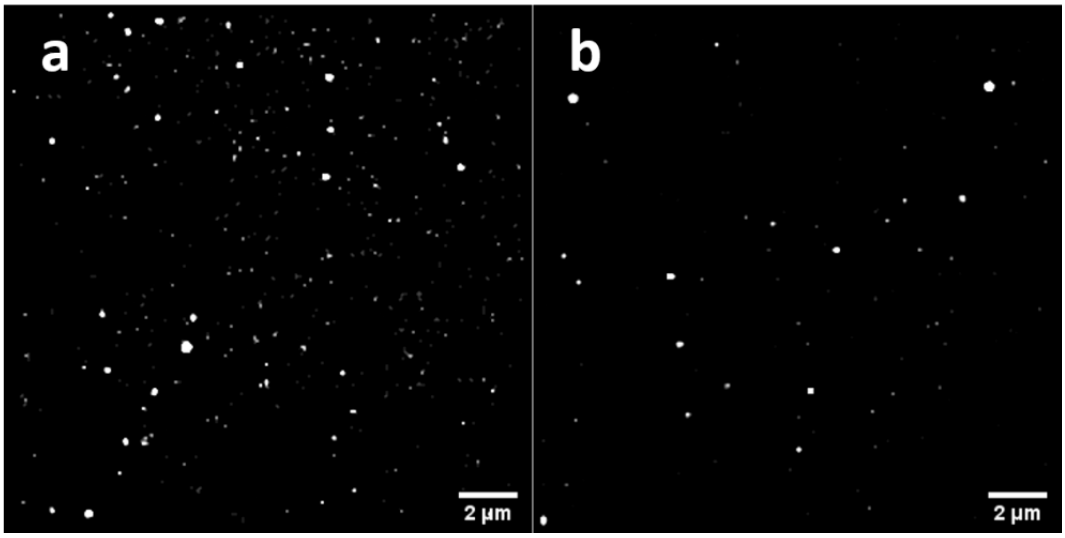

Figure S34. Reconstructed SMLM images measured in different pH solution, (a) H_2O (pH 7) and (b) 0.1 M HCl (pH 1).

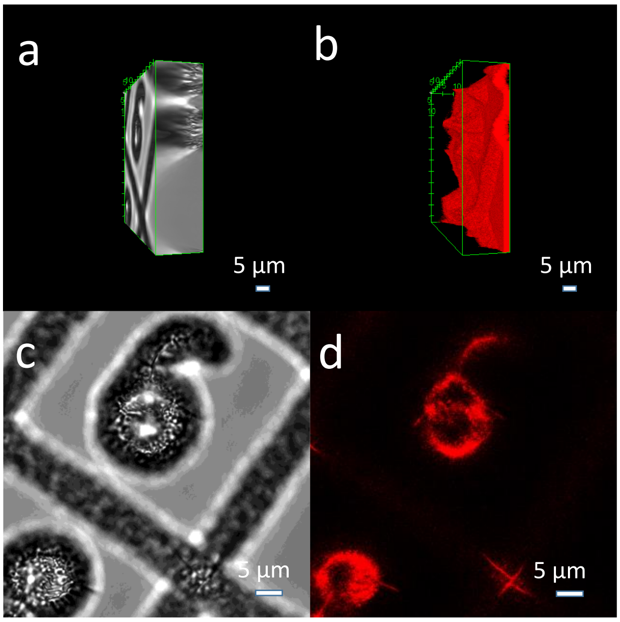

Figure S35. The repeated 3D confocal microscopy images of gridded structures in a glass substrate with N-DBOV **10** (at the same position). Reconstructions of (a) bright-field 3D image and (b) confocal fluorescence 3D image. (c) Bright-field image and (d) confocal fluorescence image, both at the same imaging depth as shown in Figure 7.

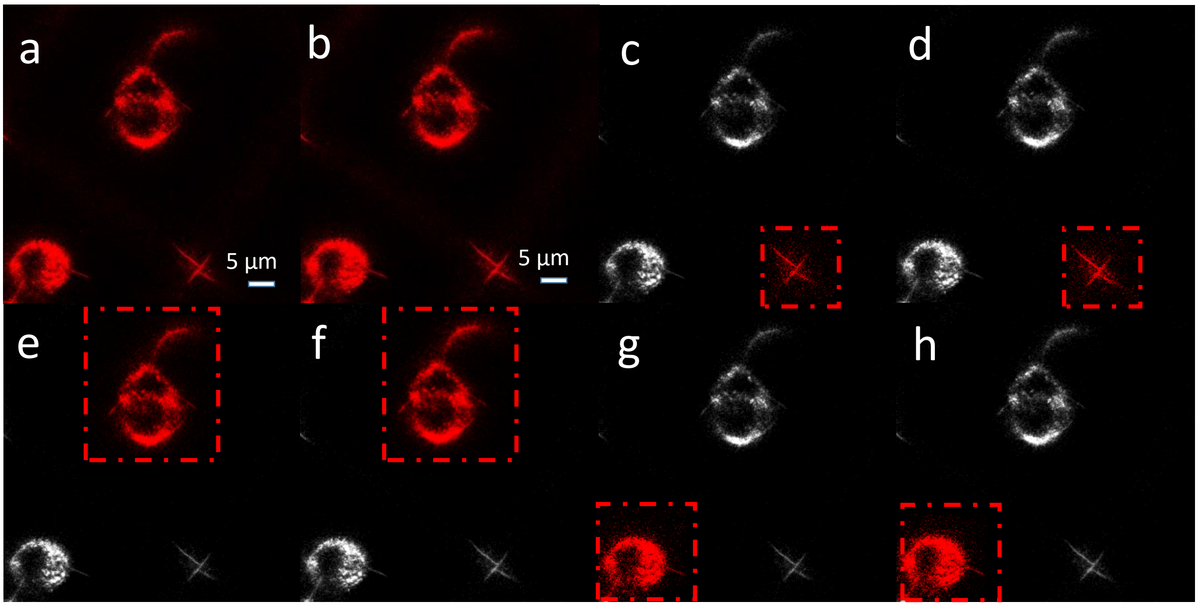

Figure S36. Comparison of the same frame of two 3D confocal imaging data shown in Figure 7 and Figure S35, respectively. (a, c, e, g: first test; b, d, f, h: second test).

For 3D confocal imaging, the total depth measured was 17.5 μm and step size was set to 0.13 μm. All the images were acquired with 2 times line average and one 3D imaging took about 8 minutes to complete. For the fluorescence intensity analysis of confocal images, we measured the gray value of different regions of interest in one frame. After cropping the region of interest, we adjusted the threshold to the default value, and the gray value obtained only comes from the fluorescent signal in the region of interest (excluding the background).

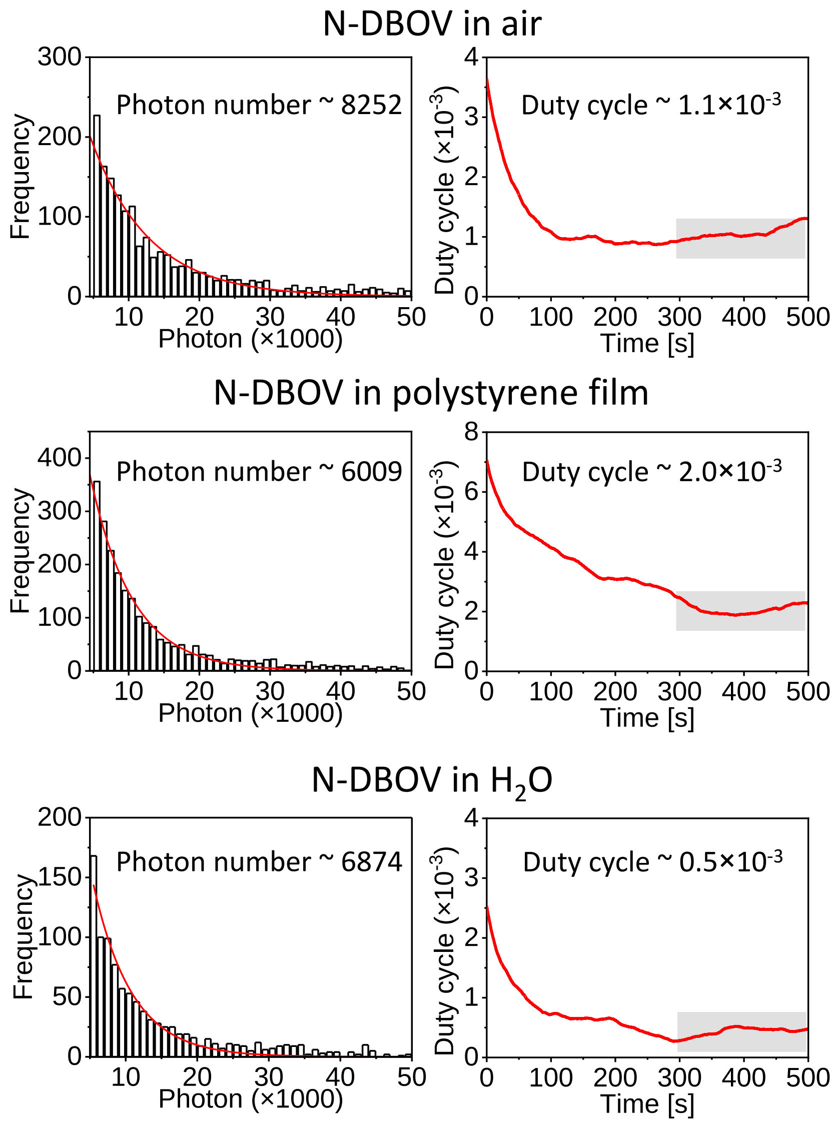

Figure S37. Blinking properties of N-DBOV **10** in air, embedded in polystyrene film, and in H_2O. Left: Histogram of detected photons per switching event and single-exponential fit. Right: On-off duty cycle (fraction of time a molecule resides in its fluorescent state).

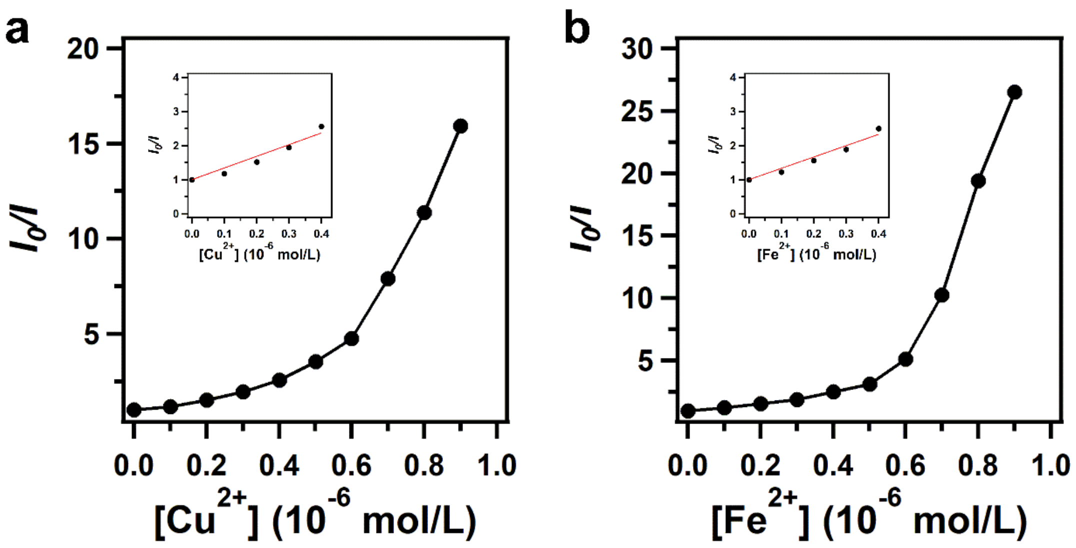

Figure S38. Stern–Volmer plots of N-DBOV **10** for Cu^{2+} and Fe^{2+} sensing. Inset: Stern–Volmer plots at lower concentration region.

Table S1. Characteristic electron transitions for N-DBOV **10** and DBOV **11** calculated using TD-DFT at the B3LYP/6-31G(d) level (Oscillator Strength > 0).

Compound	Excited State	Energy (eV)	Wavelength (nm)	Oscillator Strength	Description
N-DBOV **10**	1	2.0507	604.59	0.6105	H -> L 99.0%
	3	2.7937	443.8	0.1468	H-2 -> L 82.7%, H -> L+2 15.6%
	8	3.4759	356.7	0.4546	H -> L+2 76.8%, H-2 -> L 12.5%
DBOV **11**	1	2.0176	614.51	0.666	H -> L 99.3%
	3	2.8593	433.62	0.0011	H-2 -> L 49.6%, H -> L+2 49.3%
	7	3.3453	370.62	0.5397	H -> L+2 43.4%, H-2 -> L 42.2%, H -> L+5 5.5%, H-1 -> L+1 5.1%

Excited State Calculation

The non-radiative decay rate ($\boldsymbol{k_{nr}}$) of rigid nanographene molecules with similar backbones do not show significant differences,[15, 16] and similar electronic structures, such as frontier molecular orbital (FMO) and excitation mode, can be observed in N-DBOV **10** with different degrees of protonation. Therefore, we mainly focused on the difference in the radiation decay rate ($\boldsymbol{k_r}$).

The lifetime $\boldsymbol{\tau_r}$ (in seconds) is calculated by eq. (1)[17, 18]

$$\tau_r = \frac{2}{3f\tilde{v}} \qquad (1)$$

where $\tilde{v}$ is the transition energy in cm^{-1}, and the oscillator strengths f are computed with the length gauge. Both two parameters are calculated with TD-DFT.

The radiation decay rate $\boldsymbol{k_r}$ is calculated by eq. (2)

$$k_r = \frac{1}{\tau_r} \qquad (2)$$

The estimated results of the $\boldsymbol{k_r}$ of N-DBOV **10** with different degrees of protonation are summarized in Table S3, indicating red shift of the emission wavelength and the decrease in oscillator strengths upon the protonation. The estimated $\boldsymbol{k_r}$ (1.82×10^8 s^{-1}) of N-DBOV **10** agreed with the experimental result of 1.23×10^8 s^{-1}, which was estimated from the PLQY and fluorescence lifetime, using eq. (2) and the equation ($\Phi_f = \boldsymbol{k_r}/(\boldsymbol{k_r}+\boldsymbol{k_{nr}})$). These results further support our conclusion that decreased PLQY of N-DBOV **10** with different degrees of protonation comes from decreased radiation decay rate.

Table S2. Calculation results of emission wavelength, oscillator strength and radiation decay rate of N-DBOV **10** with different degrees of protonation.

**Molecules**	***λ_{em}/nm***	***Oscillator Strength***	***k_r/s^{-1}***
N-DBOV **10**	621.01	1.0504	1.82×10^8
N-DBOV-H$^+$	658.59	0.9248	1.42×10^8
N-DBOV-2H$^+$	776.85	0.6124	6.77×10^7

Table S3. Calculated gray value of the fluorescent part of whole frame and other selected regions of interest in **Figure S36**.

	Area	Mean gray value
a	80.941	39.123
b	93.583	38.978
c	3.294	27.775
d	4.293	28.115
e	38.667	36.270
f	41.025	38.062
g	24.727	52.818
h	29.683	51.040

Appendix

Cartesian coordinates

DBOV 11 ground state(S0)

C	5.84828481	0.07896809	-0.00001379
C	4.88969136	-0.96154843	-0.00002296
C	3.51482365	-0.73578271	-0.00003062
C	3.04696511	0.61519201	-0.00002015
C	4.00009162	1.68239093	-0.00001153
C	5.38602277	1.38303417	-0.00001006
C	1.65605588	0.91368919	-0.00001458
C	1.22058818	2.25922360	-0.00000363
C	2.18743963	3.32576898	-0.00000028
C	3.54004694	3.01811804	-0.00000356
C	-0.17383098	2.56366354	0.00000461
C	-0.59772418	3.92430545	0.00001181
C	0.38866131	4.96996709	0.00001272
C	1.71684195	4.68605349	0.00000763
C	2.52956765	-1.82366695	-0.00004796
C	1.13979351	-1.51176700	-0.00003665
C	0.68855315	-0.14918328	-0.00001857
C	-0.68855315	0.14918328	-0.00000737
C	-1.13979352	1.51176700	0.00000585
C	-2.52956765	1.82366695	0.00001728
C	-2.90749592	3.17865496	0.00001813
C	-1.97266560	4.20023206	0.00001616
C	2.90749592	-3.17865496	-0.00007698
C	1.97266560	-4.20023206	-0.00008692
C	0.59772418	-3.92430545	-0.00006963
C	0.17383098	-2.56366354	-0.00004603
C	-1.22058818	-2.25922360	-0.00003162
C	-1.65605588	-0.91368919	-0.00001177
C	-3.04696511	-0.61519201	0.00000536
C	-3.51482365	0.73578271	0.00002632
C	-0.38866131	-4.96996709	-0.00007663
C	-1.71684195	-4.68605348	-0.00006032
C	-2.18743963	-3.32576898	-0.00003672
C	-3.54004694	-3.01811804	-0.00001760
C	-4.00009162	-1.68239093	0.00000550
C	-5.38602277	-1.38303417	0.00002992
C	-5.84828481	-0.07896809	0.00005687
C	-4.88969136	0.96154843	0.00005551
H	5.25267889	-1.98169688	-0.00002148
H	6.07733776	2.21810256	-0.00000456
H	0.04426250	6.00069998	0.00001817
H	2.45374969	5.48460293	0.00000939
H	-3.95730886	3.44689505	0.00001755
H	-2.30466072	5.23500375	0.00001736
H	3.95730886	-3.44689505	-0.00009505
H	2.30466072	-5.23500375	-0.00010908
H	-0.04426250	-6.00069998	-0.00009484
H	-2.45374969	-5.48460293	-0.00006478
H	-6.07733776	-2.21810256	0.00002817
H	-5.25267889	1.98169688	0.00008105
H	-4.27013442	-3.82387094	-0.00001947
H	4.27013442	3.82387094	0.00000092
C	7.34606519	-0.27871618	-0.00000673
C	7.67809253	-1.10887222	1.26279425
C	7.67811291	-1.10883521	-1.26282725
C	8.24479062	0.97209007	0.00001855
H	7.45996821	-0.54072483	2.17281465
H	7.10352611	-2.03874687	1.30511522
H	8.74102309	-1.37475641	1.27414823
H	7.45997197	-0.54067362	-2.17283467
H	8.74105045	-1.37469069	-1.27418828
H	7.10357236	-2.03872510	-1.30516771
H	9.29684510	0.66972869	0.00002716
H	8.07992291	1.59205051	-0.88716463
H	8.07990296	1.59202897	0.88721288
C	-7.34606519	0.27871618	0.00008981
C	-7.67807644	1.10884784	1.26291105
C	-7.67812899	1.10885959	-1.26271044
C	-8.24479062	-0.97209008	0.00010241
H	-7.45994053	0.54068288	2.17291770
H	-7.10350949	2.03872167	1.30524265
H	-8.74100684	1.37473181	1.27428371
H	-7.45999964	0.54071558	-2.17273163
H	-8.74106669	1.37471528	-1.27405280
H	-7.10358903	2.03875030	-1.30504027
H	-9.29684510	-0.66972869	0.00013013
H	-8.07993412	-1.59203348	-0.88709476
H	-8.07989174	-1.59204600	0.88728275

N-DBOV 10 ground state(S0)

C	-5.81480350	0.00240005	0.00001199
C	-4.86578624	1.04967574	-0.00001090
C	-3.48829320	0.82040523	-0.00000927
C	-3.03790686	-0.53224809	0.00001701
C	-3.97404542	-1.61301748	0.00004099
C	-5.35403099	-1.30930125	0.00003777
C	-1.66085682	-0.84844098	0.00002034
C	-1.26869203	-2.19771886	0.00004661
C	-2.29374121	-3.20754173	0.00006929
N	-3.59452132	-2.92318615	0.00006659
C	0.11042268	-2.55100427	0.00005057
C	0.48410243	-3.92212193	0.00007716
C	-0.55336299	-4.92234415	0.00009964
C	-1.87299863	-4.58877711	0.00009608
C	-2.48220183	1.89421936	-0.00003314
C	-1.09858767	1.55461138	-0.00002934
C	-0.67669386	0.18639398	-0.00000269
C	0.68352716	-0.16095797	0.00000133
C	1.10473918	-1.52910349	0.00002790
C	2.48841454	-1.86978798	0.00003174
C	2.82178177	-3.23714776	0.00005812
C	1.85305297	-4.23327120	0.00008016
C	-2.81615676	3.26247537	-0.00006001

C	-1.84817946	4.25832008	-0.00008221
C	-0.47835506	3.94759660	-0.00007894
C	-0.10466577	2.57680592	-0.00005213
C	1.27478741	2.22390817	-0.00004800
C	1.66791669	0.87439022	-0.00002165
C	3.04418963	0.55957481	-0.00001806
C	3.49569672	-0.79579082	0.00000845
C	0.55855240	4.94770762	-0.00010139
C	1.87855881	4.61400288	-0.00009751
C	2.29853527	3.23319073	-0.00007048
N	3.60053485	2.94767641	-0.00006729
C	3.97917039	1.63926553	-0.00004157
C	5.36480715	1.33887717	-0.00003769
C	5.82249975	0.03092842	-0.00001160
C	4.86954978	-1.01983161	0.00001091
C	-7.33332752	0.27457620	0.00000984
C	-7.66744183	1.77850437	-0.00001637
C	-7.96551177	-0.35816987	-1.26237691
C	-7.96550727	-0.35812471	1.26242139
C	7.32169356	-0.32280144	-0.00000696
C	8.21578430	0.93117393	-0.00003908
C	7.65522768	-1.15198336	1.26314544
C	7.65522049	-1.15204354	-1.26312175
H	-5.22436640	2.06970176	-0.00003045
H	-6.03569235	-2.15280190	0.00005654
H	-0.25517467	-5.96760947	0.00011992
H	-2.65289315	-5.34286477	0.00011306
H	3.86372646	-3.53685184	0.00006161
H	2.15921032	-5.27599855	0.00010020
H	-3.85812113	3.56189240	-0.00006381
H	-2.15448386	5.30101224	-0.00010256
H	0.26043147	5.99298680	-0.00012186
H	2.65844564	5.36810133	-0.00011440
H	6.03973172	2.18517363	-0.00005574
H	5.23640952	-2.03936735	0.00003067
H	-8.75381807	1.91158792	-0.00001706
H	-7.27549014	2.28556579	0.88790754
H	-7.27549289	2.28553430	-0.88795950
H	-9.04678488	-0.18124764	-1.27503717
H	-7.80379922	-1.43901365	-1.29996075
H	-7.53987932	0.07507800	-2.17336794
H	-7.53987244	0.07515642	2.17339547
H	-9.04678049	-0.18120289	1.27507854
H	-7.80379388	-1.43896702	1.30004387
H	9.26861672	0.63165592	-0.00003249
H	8.04835812	1.55074889	-0.88671633
H	8.04835939	1.55079317	0.88660750
H	8.71916087	-1.41324104	1.27465141
H	7.08517636	-2.08479369	1.30545611
H	7.43495384	-0.58452215	2.17301630
H	7.43494321	-0.58462501	-2.17301838
H	8.71915325	-1.41330357	-1.27462040
H	7.08516720	-2.08485482	-1.30538543

N-DBOV_H$^+$ ground state(S0)

C	-5.83009300	-0.01170300	0.00001200
C	-4.88236600	1.03454300	-0.00001200
C	-3.49905200	0.81103100	-0.00001000
C	-3.04147300	-0.53528700	0.00001700
C	-3.97409700	-1.61643300	0.00004100
C	-5.35703600	-1.32145300	0.00003800
C	-1.66587100	-0.84812800	0.00002000
C	-1.26898800	-2.19690100	0.00004700
C	-2.29146900	-3.21069200	0.00007000
N	-3.59114400	-2.92173900	0.00006700
C	0.10792500	-2.54984100	0.00005100
C	0.47737100	-3.92240500	0.00007800
C	-0.55740700	-4.92559700	0.00010100
C	-1.87683100	-4.59246900	0.00009700
C	-2.50500900	1.88407100	-0.00003400
C	-1.11680800	1.55069800	-0.00003000
C	-0.68673000	0.18826300	-0.00000300
C	0.68236500	-0.15780500	0.00000100
C	1.10249100	-1.52854800	0.00002800
C	2.48036300	-1.87383000	0.00003200
C	2.81418900	-3.24357200	0.00005900
C	1.84702900	-4.23789100	0.00008100
C	-2.85213700	3.25638700	-0.00006100
C	-1.89584600	4.25245100	-0.00008300
C	-0.51637400	3.94633400	-0.00008000
C	-0.13166800	2.57939700	-0.00005300
C	1.24753100	2.23293100	-0.00004900
C	1.65855900	0.87329600	-0.00002200
C	3.03997400	0.54755300	-0.00001800
C	3.48893600	-0.80873300	0.00000900
C	0.49880600	4.94537200	-0.00010300
C	1.83419800	4.62251000	-0.00009900
C	2.22628100	3.25979700	-0.00007200
N	3.54107200	2.89976200	-0.00006700
C	3.99910900	1.59327900	-0.00004100
C	5.36912400	1.31756000	-0.00003800
C	5.82041200	-0.00366200	-0.00001200
C	4.86567900	-1.04075000	0.00001100
C	-7.34842900	0.25313900	0.00001000
C	-7.68457400	1.75635500	-0.00002000
C	-7.97366800	-0.38471800	-1.26385000
C	-7.97366100	-0.38466700	1.26389900
C	7.31831500	-0.35620800	-0.00000500
C	8.21475700	0.89632000	-0.00002800
C	7.64456000	-1.18690100	1.26513200
C	7.64456100	-1.18694700	-1.26511300
H	-5.24805200	2.05198400	-0.00003200
H	-6.03333200	-2.16890300	0.00005700
H	-0.25788200	-5.96952500	0.00012200
H	-2.65737700	-5.34518400	0.00011500
H	3.85487800	-3.54562400	0.00006300
H	2.15182700	-5.28007900	0.00010200
H	-3.89544600	3.54706400	-0.00006500
H	-2.20571000	5.29341900	-0.00010400
H	0.20253700	5.99017700	-0.00012300
H	2.59172600	5.40031400	-0.00011600
H	6.07084900	2.14346800	-0.00005600
H	5.22641400	-2.06090000	0.00003200
H	-8.77046300	1.88537500	-0.00002200
H	-7.29790700	2.26525100	0.88952100
H	-7.29790700	2.26521600	-0.88958000

H	-9.05442900	-0.21142000	-1.27487400
H	-7.81199900	-1.46577600	-1.30015300
H	-7.55202800	0.04941300	-2.17609300
H	-7.55201700	0.04950200	2.17612300
H	-9.05442300	-0.21137000	1.27492100
H	-7.81199000	-1.46572300	1.30024500
H	9.26492700	0.59202100	-0.00002400
H	8.05657000	1.51513800	-0.88957800
H	8.05657300	1.51516800	0.88950200
H	8.70806600	-1.44495900	1.27551800
H	7.07856200	-2.12195500	1.30336400
H	7.42443900	-0.62249100	2.17670000
H	7.42443900	-0.62257000	-2.17670100
H	8.70806700	-1.44500300	-1.27548900
H	7.07856500	-2.12200300	-1.30331100
H	4.23534900	3.63695800	-0.00008300

N-DBOV_2H$^+$ ground state(S0)

C	-5.83185500	0.02023600	0.00001200
C	-4.87249600	1.05735400	-0.00000900
C	-3.49546800	0.82346500	-0.00000800
C	-3.04222400	-0.52666500	0.00001500
C	-4.00275200	-1.57393700	0.00003500
C	-5.37314800	-1.29892600	0.00003400
C	-1.66387500	-0.85293200	0.00001700
C	-1.25161600	-2.20437700	0.00004000
C	-2.23553300	-3.23738400	0.00006000
N	-3.54258700	-2.87644700	0.00005700
C	0.12684500	-2.55280600	0.00004300
C	0.50651400	-3.92090100	0.00006500
C	-0.51411200	-4.92442600	0.00008500
C	-1.84589900	-4.60377300	0.00008200
C	-2.49042000	1.89332200	-0.00003000
C	-1.10746000	1.55119000	-0.00002600
C	-0.68006500	0.18578000	-0.00000300
C	0.68683300	-0.16174200	0.00000100
C	1.11389600	-1.52717000	0.00002400
C	2.49701100	-1.87030500	0.00002700
C	2.84082100	-3.23643900	0.00004900
C	1.87837500	-4.23456900	0.00006700
C	-2.83485700	3.26018700	-0.00005400
C	-1.87316000	4.25835200	-0.00007400
C	-0.50068200	3.94510900	-0.00007000
C	-0.12093900	2.57720200	-0.00004600
C	1.25769400	2.22915100	-0.00004300
C	1.67061300	0.87769000	-0.00001900
C	3.04852600	0.55252900	-0.00001600
C	3.50311100	-0.80017300	0.00000800
C	0.51969000	4.94881700	-0.00009000
C	1.85163500	4.62835300	-0.00008700
C	2.24073900	3.26200400	-0.00006300
N	3.54833100	2.89975100	-0.00005900
C	4.00703500	1.59888200	-0.00003600
C	5.38293100	1.32719300	-0.00003200
C	5.83903900	0.01240700	-0.00000800
C	4.87654100	-1.02910200	0.00001100
C	-7.34466100	0.29961400	0.00001000
C	-7.66366500	1.80654300	-0.00002700
C	-7.97291400	-0.33467900	-1.26619700
C	-7.97290700	-0.33461500	1.26625200
C	7.33377900	-0.34155300	-0.00000300
C	8.22815200	0.91202700	-0.00005200
C	7.65401500	-1.17412000	1.26693800
C	7.65400200	-1.17421100	-1.26688700
H	-5.23349300	2.07545400	-0.00002500
H	-6.07808600	-2.12414000	0.00004900
H	-0.21646300	-5.96860900	0.00010200
H	-2.60366200	-5.38099800	0.00009700
H	3.88191300	-3.53418800	0.00005100
H	2.18720600	-5.27537800	0.00008400
H	-3.87607100	3.55737300	-0.00005800
H	-2.18225800	5.29907000	-0.00009200
H	0.22183700	5.99292900	-0.00010900
H	2.60939700	5.40558400	-0.00010300
H	6.08334500	2.15439100	-0.00004800
H	5.24388700	-2.04674800	0.00002900
H	-8.74770000	1.94418700	-0.00002200
H	-7.27572700	2.31163000	0.89087000
H	-7.27574200	2.31158300	-0.89095900
H	-9.04930900	-0.14161200	-1.27710100
H	-7.83585600	-1.41966000	-1.29904700
H	-7.54517700	0.08997700	-2.17960700
H	-7.54516300	0.09008600	2.17963800
H	-9.04930200	-0.14154400	1.27715400
H	-7.83585200	-1.41959400	1.29915500
H	9.27759900	0.60759800	-0.00003900
H	8.07279200	1.52987300	-0.89085300
H	8.07279000	1.52994500	0.89069800
H	8.71682700	-1.43197300	1.27490000
H	7.09111100	-2.11151300	1.30422100
H	7.43702200	-0.61071000	2.17958000
H	7.43700400	-0.61086500	-2.17956700
H	8.71681300	-1.43206900	-1.27483800
H	7.09109300	-2.11160500	-1.30409800
H	4.24436600	3.63868900	-0.00007300
H	-4.23789000	-3.61590200	0.00007100

N-DBOV 10 excited state(S1)

C	-5.79902270	0.00314821	0.00001877
C	-4.86972504	1.04598781	-0.00001192
C	-3.47530737	0.81102593	-0.00001386
C	-3.02602832	-0.53263618	0.00001894
C	-3.95405609	-1.61869878	0.00005191
C	-5.33134585	-1.31451006	0.00005046
C	-1.64809164	-0.84082451	0.00002138
C	-1.24836156	-2.19996040	0.00005550
C	-2.26141425	-3.20280520	0.00008586
N	-3.57194304	-2.92160393	0.00008429
C	0.12587486	-2.54576530	0.00005934
C	0.50726191	-3.90756164	0.00009290
C	-0.51261299	-4.90749672	0.00012292
C	-1.83820305	-4.57567217	0.00011991
C	-2.48938139	1.87457371	-0.00004709
C	-1.11117553	1.54534883	-0.00004323
C	-0.68720453	0.18445182	-0.00000875
C	0.69323553	-0.15720395	-0.00000438

C	1.11728195	-1.51914340	0.00002986
C	2.49384323	-1.84902247	0.00003435
C	2.84682511	-3.23229053	0.00006662
C	1.89450918	-4.21817022	0.00009478
C	-2.84182647	3.25879116	-0.00008408
C	-1.88968008	4.24423753	-0.00011356
C	-0.50191394	3.93387602	-0.00010879
C	-0.12064691	2.57236019	-0.00007371
C	1.25404047	2.22693447	-0.00006851
C	1.65384460	0.86851564	-0.00003395
C	3.03220028	0.56152645	-0.00002879
C	3.48224075	-0.78436496	0.00000617
C	0.51757123	4.93373333	-0.00013830
C	1.84348647	4.60189264	-0.00013298
C	2.26607181	3.22936555	-0.00009738
N	3.57751436	2.94713611	-0.00009192
C	3.95876833	1.64610508	-0.00005755
C	5.34287112	1.34601459	-0.00004965
C	5.80623030	0.03397120	-0.00001505
C	4.87101185	-1.01295003	0.00001286
C	-7.31064476	0.25501645	0.00002494
C	-7.65276272	1.74828905	0.00004739
C	-7.92872670	-0.38343043	-1.25559503
C	-7.92871934	-0.38346705	1.25562965
C	7.30022113	-0.30668166	0.00000381
C	8.17700656	0.94955749	0.00003945
C	7.63357253	-1.12928416	1.25628750
C	7.63362217	-1.12924526	-1.25629197
H	-5.22888943	2.06645630	-0.00003495
H	-6.01925315	-2.15509475	0.00007754
H	-0.21227639	-5.95169869	0.00014860
H	-2.61120167	-5.33724570	0.00014320
H	3.89139042	-3.52107613	0.00006773
H	2.19629759	-5.26169306	0.00011893
H	-3.88616004	3.54817940	-0.00009028
H	-2.19147687	5.28776042	-0.00014149
H	0.21726320	5.97793091	-0.00016522
H	2.61639151	5.36356681	-0.00015534
H	6.02211330	2.19082224	-0.00007329
H	5.23986678	-2.03263511	0.00004468
H	-8.74042136	1.86961090	0.00002707
H	-7.26056787	2.25305710	0.88934453
H	-7.26052935	2.25309235	-0.88921294
H	-9.01062387	-0.21209743	-1.26668956
H	-7.75893589	-1.46390009	-1.28582053
H	-7.50149116	0.05432737	-2.16370375
H	-7.50148799	0.05427334	2.16374878
H	-9.01061842	-0.21214655	1.26672896
H	-7.75891536	-1.46393557	1.28582828
H	9.23108615	0.65509822	0.00002519
H	8.00061892	1.56471568	-0.88841374
H	8.00062950	1.56465897	0.88853385
H	8.70094410	-1.37533963	1.26798221
H	7.07271910	-2.06837715	1.28875758
H	7.40111698	-0.56234672	2.16364590
H	7.40118551	-0.56228691	-2.16364218
H	8.70099808	-1.37528299	-1.26796089
H	7.07278710	-2.06834741	-1.28880603

N-DBOV_H$^+$ excited state(S1)

C	-5.81369461	-0.01310180	0.00001508
C	-4.88264884	1.03124294	-0.00000172
C	-3.48830992	0.80493580	-0.00000128
C	-3.03444805	-0.53531333	0.00001810
C	-3.96074424	-1.62158874	0.00003513
C	-5.34154531	-1.32688342	0.00003240
C	-1.65672419	-0.84042647	0.00001981
C	-1.25853856	-2.19498031	0.00003834
C	-2.26956197	-3.20009023	0.00005466
N	-3.57762853	-2.92176715	0.00005280
C	0.11392929	-2.53927078	0.00003906
C	0.49215952	-3.90263510	0.00005603
C	-0.52370218	-4.90508012	0.00007303
C	-1.85074361	-4.57392298	0.00007260
C	-2.50436995	1.87326494	-0.00002072
C	-1.12685249	1.54677919	-0.00001841
C	-0.69622269	0.18832303	0.00000211
C	0.68433225	-0.15141586	0.00000349
C	1.10343930	-1.51604029	0.00002169
C	2.48078373	-1.85490049	0.00002129
C	2.83339656	-3.23615199	0.00003700
C	1.87784331	-4.21903899	0.00005418
C	-2.86211735	3.25391421	-0.00004400
C	-1.91590281	4.24500084	-0.00006343
C	-0.52735682	3.93746452	-0.00006110
C	-0.14192610	2.57754499	-0.00003846
C	1.23378287	2.23340881	-0.00003587
C	1.64625623	0.87253818	-0.00001507
C	3.02840607	0.55016808	-0.00001402
C	3.47346924	-0.79503745	0.00000481
C	0.48421531	4.93926355	-0.00008280
C	1.81736671	4.61548598	-0.00008053
C	2.20764724	3.25395723	-0.00005514
N	3.52591834	2.89323967	-0.00004910
C	3.97838496	1.59576761	-0.00003083
C	5.35194799	1.31516913	-0.00002899
C	5.80444227	-0.00419651	-0.00000884
C	4.85871294	-1.03779371	0.00000733
C	-7.32501409	0.23593233	0.00000738
C	-7.66782237	1.72886928	-0.00008353
C	-7.93946207	-0.40508449	-1.25628879
C	-7.93945381	-0.40492851	1.25638731
C	7.29450893	-0.35377353	-0.00000545
C	8.17998979	0.89619567	-0.00006072
C	7.61773092	-1.17886196	1.25779249
C	7.61771325	-1.17896172	-1.25774223
H	-5.24649171	2.05015768	-0.00001308
H	-6.02366521	-2.17185759	0.00004202
H	-0.22219074	-5.94833434	0.00008533
H	-2.62408231	-5.33469644	0.00008496
H	3.87600931	-3.52920245	0.00003419
H	2.17605577	-5.26304412	0.00006590
H	-3.90743902	3.53938930	-0.00004819
H	-2.22183082	5.28664668	-0.00008182
H	0.18515178	5.98290322	-0.00010189
H	2.57736288	5.39105154	-0.00010085
H	6.05207277	2.14312325	-0.00004336

H	5.21734247	-2.06005053	0.00002201
H	-8.75541618	1.84792490	-0.00005738
H	-7.27757653	2.23431607	0.88971869
H	-7.27763400	2.23419542	-0.88997936
H	-9.02131156	-0.23577674	-1.26645721
H	-7.76886612	-1.48552054	-1.28598513
H	-7.51380469	0.03332444	-2.16466184
H	-7.51377966	0.03358303	2.16470293
H	-9.02130097	-0.23560544	1.26654917
H	-7.76887278	-1.48536317	1.28621054
H	9.23061968	0.59176001	-0.00004049
H	8.01240481	1.51189734	-0.88998240
H	8.01239470	1.51198249	0.88980021
H	8.68321159	-1.43067388	1.26854683
H	7.05246521	-2.11518906	1.28889712
H	7.38888484	-0.61079009	2.16515174
H	7.38885626	-0.61096124	-2.16514346
H	8.68319317	-1.43077691	-1.26849068
H	7.05244479	-2.11529005	-1.28876520
H	4.21798314	3.63608416	-0.00005357

N-DBOV_2H$^+$ excited state(S1)

C	-5.82890900	0.02250600	0.00003900
C	-4.88476900	1.05502800	0.00000600
C	-3.48833200	0.81582400	-0.00001100
C	-3.03473000	-0.53191800	0.00001200
C	-3.98674500	-1.58111100	0.00004600
C	-5.36088600	-1.30124600	0.00005900
C	-1.65107300	-0.84898400	0.00000600
C	-1.23767700	-2.21125700	0.00002800
C	-2.21339700	-3.23715700	0.00005700
N	-3.53130800	-2.88251300	0.00006400
C	0.13883900	-2.55195300	0.00002400
C	0.52683500	-3.92165900	0.00004300
C	-0.48466400	-4.92623500	0.00006800
C	-1.82109200	-4.60293900	0.00007500
C	-2.50145800	1.87931300	-0.00005100
C	-1.11554200	1.54534700	-0.00005000
C	-0.68815900	0.18021700	-0.00002100
C	0.69493600	-0.15623300	-0.00001900
C	1.12234800	-1.52184800	0.00000300
C	2.50599300	-1.85594700	0.00000600
C	2.86368800	-3.23667100	0.00001800
C	1.91287300	-4.23105600	0.00003700
C	-2.85935100	3.26134900	-0.00009300
C	-1.90913900	4.25514700	-0.00012500
C	-0.52237200	3.94550400	-0.00011500
C	-0.13365500	2.57553800	-0.00007900
C	1.24344600	2.23561000	-0.00006800
C	1.65762200	0.87408500	-0.00003800
C	3.04130700	0.55838900	-0.00002600
C	3.49508500	-0.79020800	-0.00000200
C	0.48832000	4.95044800	-0.00014500
C	1.82492600	4.62742200	-0.00013100
C	2.21802300	3.26097500	-0.00008600
N	3.53594200	2.90714300	-0.00006000
C	3.99279600	1.60757700	-0.00003200
C	5.37180000	1.33270800	-0.00001000
C	5.83549600	0.01408500	0.00001600
C	4.88600600	-1.02298000	0.00001900
C	-7.34498600	0.28310800	0.00005200
C	-7.67967400	1.78636100	0.00002300
C	-7.96863700	-0.35548000	-1.26515300
C	-7.96860700	-0.35542400	1.26530100
C	7.33496800	-0.32915700	0.00004100
C	8.22253500	0.92944800	0.00004700
C	7.66524900	-1.15724000	1.26584400
C	7.66528500	-1.15725300	-1.26574500
H	-5.23721800	2.07576200	-0.00000300
H	-6.06336100	-2.12796200	0.00008400
H	-0.18623000	-5.96992000	0.00008000
H	-2.57868600	-5.38131000	0.00009500
H	3.90754200	-3.52537300	0.00001300
H	2.22119200	-5.27188000	0.00004700
H	-3.90313400	3.54977100	-0.00010400
H	-2.21705100	5.29604200	-0.00015700
H	0.18964700	5.99401500	-0.00017800
H	2.58212500	5.40619800	-0.00016100
H	6.06789500	2.16288700	-0.00001500
H	5.24532500	-2.04335500	0.00004100
H	-8.76694600	1.91303900	0.00003400
H	-7.29034300	2.29366300	0.88999500
H	-7.29036600	2.29362300	-0.88998300
H	-9.05012000	-0.18129100	-1.27042700
H	-7.80796200	-1.43800600	-1.30660100
H	-7.54873200	0.08464900	-2.17625100
H	-7.54868300	0.08474800	2.17636900
H	-9.05009000	-0.18123700	1.27059100
H	-7.80792800	-1.43794700	1.30679400
H	9.27471900	0.62790500	0.00006700
H	8.05907100	1.54801000	-0.88951400
H	8.05904100	1.54802200	0.88959500
H	8.73162200	-1.40822000	1.27338700
H	7.10397200	-2.09652700	1.30522200
H	7.44258700	-0.59020200	2.17660700
H	7.44265000	-0.59022300	-2.17651900
H	8.73165900	-1.40823300	-1.27325500
H	7.10400900	-2.09653900	-1.30512900
H	4.22515000	3.65292100	-0.00004700
H	-4.22092900	-3.62763500	0.00008400

Reference

(1) Sens, R.; Drexhage, K. H. Fluorescence quantum yield of oxazine and carbazine laser dyes. *J. Lumin.* **1981**, *24-25*, 709-712.

(2) Gaussian 16, Revision A.03, Frisch, M. J.; Trucks, G. W.; Schlegel, H. B.; Scuseria, G. E.; Robb, M. A.; Cheeseman, J. R.; Scalmani, G.; Barone, V.; Petersson, G. A.; Nakatsuji, H.; Li, X.; Caricato, M.; Marenich, A. V.; Bloino, J.; Janesko, B. G.; Gomperts, R.; Mennucci, B.; Hratchian, H. P.; Ortiz, J. V.; Izmaylov, A. F.; Sonnenberg, J. L.; Williams-Young, D.; Ding, F.; Lipparini, F.; Egidi, F.; Goings, J.; Peng, B.; Petrone, A.; Henderson, T.; Ranasinghe, D.; Zakrzewski, V. G.; Gao, J.; Rega, N.; Zheng, G.; Liang, W.; Hada, M.; Ehara, M.; Toyota, K.; Fukuda, R.; Hasegawa, J.; Ishida, M.; Nakajima, T.; Honda, Y.; Kitao, O.; Nakai, H.; Vreven, T.; Throssell, K.; Montgomery, Jr., J. A.; Peralta, J. E.; Ogliaro, F.; Bearpark, M. J.; Heyd, J. J.; Brothers, E. N.; Kudin, K. N.; Staroverov, V. N.; Keith, T. A.; Kobayashi, R.; Normand, J.; Raghavachari, K.; Rendell, A. P.; Burant, J. C.; Iyengar, S. S.; Tomasi, J.; Cossi, M.; Millam, J. M.; Klene, M.; Adamo, C.; Cammi, R.; Ochterski, J. W.; Martin, R. L.; Morokuma, K.; Farkas, O.; Foresman, J. B.; Fox, D. J. Gaussian, Inc., Wallingford, CT, **2016**.

(3) Geuenich, D.; Hess, K.; Köhler, F.; Herges, R., Anisotropy of the induced current density (ACID), a general method to quantify and visualize electronic delocalization. *Chem. Rev.* **2005,** *105* (10), 3758-3772.

(4) Lu, T.; Chen, F., Multiwfn: a multifunctional wavefunction analyzer. *J. Comput. Chem.* **2012,** *33* (5), 580-592.

(5) Feixas, F.; Matito, E.; Poater, J.; Solà, M., Wiley Interdiscip. *Rev.: Comput. Mol. Sci.* **2013,** *3*, 105-122.

(6) Popelier, P.; Aicken, F.; O'Brien, S., Atoms in molecules. *Chemical Modelling: Applications and Theory* **2000,** *1*, 143-198.

(7) Zhao, Y.; Schultz, N. E.; Truhlar, D. G., Design of density functionals by combining the method of constraint satisfaction with parametrization for thermochemistry, thermochemical kinetics, and noncovalent interactions. *J. Chem. Theory and Comput.* **2006,** *2* (2), 364-382.

(8) Francl, M.; Pietro, W.; Hehre, W.; Binkley, J.; Gordon, M.; DeFrees, D.; Pople, J., Nonlinear optical properties of organic crystals with hydrogen-bonded molecular units: The case of urea. *J. Chem. Phys.* **1982,** *77*, 3654.

(9) Paternò, G. M.; Chen, Q.; Wang, X. Y.; Liu, J.; Motti, S. G.; Petrozza, A.; Feng, X.; Lanzani, G.; Müllen, K.; Narita, A., Synthesis of dibenzo [*hi*, *st*] ovalene and its amplified

spontaneous emission in a polystyrene matrix. *Angew. Chem. Int. Ed.* **2017,** *56* (24), 6753-6757.

(10) Chen, Q.; Thoms, S.; Stöttinger, S.; Schollmeyer, D.; Müllen, K.; Narita, A.; Basché, T., Dibenzo*[hi,st]*ovalene as highly luminescent nanographene: efficient synthesis via photochemical cyclodehydroiodination, optoelectronic properties, and single-molecule spectroscopy. *J. Am. Chem. Soc.* **2019**, *141* (41), 16439-16449.

(11) Chen, Q.; Wang, D.; Baumgarten, M.; Schollmeyer, D.; Müllen, K.; Narita, A., Regioselective bromination and functionalization of dibenzo[*hi*,*st*]ovalene as highly luminescent nanographene with zigzag Edges. *Chem. Asian J.* **2019**, *14* (10), 1703-1707.

(12) Liu, X.; Chen, S. Y.; Chen, Q.; Yao, X.; Gelleri, M.; Ritz, S.; Kumar, S.; Cremer, C.; Landfester, K.; Mullen, K.; Parekh, S. H.; Narita, A.; Bonn, M., Nanographenes: ultrastable, switchable, and bright probes for super-resolution microscopy. *Angew. Chem. Int. Ed*. **2020**, *59* (1), 496-502.

(13) Tanaka, T.; Yamagami, T.; Nogami, T.; Minami, H.; Okubo, M., Preparation of hemispherical polystyrene particles utilizing the solvent evaporation method in aqueous dispersed systems. *Polym. J.* **2012,** *44* (11), 1112-1116.

(14) Neumann, Jan. Development and application of advanced fluorescence microscopy techniques for the investigation of inflammatory processes. Diss. Universität Mainz, **2018**.

(15) Kohn, A. W.; Lin, Z.; Van Voorhis, T., Toward prediction of nonradiative decay pathways in organic compounds I: the case of naphthalene quantum yields. *J. Phys. Chem. C* **2019,** *123* (25), 15394-15402.

(16) Fernández-García, J. M.; Evans, P. J.; Medina Rivero, S.; Fernández, I.; García-Fresnadillo, D.; Perles, J.; Casado, J.; Martín, N., π-Extended corannulene-based nanographenes: selective formation of negative curvature. *J. Am. Chem. Soc.* **2018**, *140* (49), 17188-17196.

(17) Einstein, A.; Zur Theorie der Strahlung, M. d., Physikalische gesellschaft zuerich. *Mitteilungen* **1916,** *18*, 47.

(18) Cao, Z.; Zhang, Q., Computational analyses of singlet–singlet and singlet–triplet transitions in mononuclear gold-capped carbon-rich conjugated complexes. *J. Comput. Chem.* **2005,** *26* (12), 1214-1221.
